# The FGGY Carbohydrate Kinase Family: Insights into the Evolution of Functional Specificities

**DOI:** 10.1371/journal.pcbi.1002318

**Published:** 2011-12-22

**Authors:** Ying Zhang, Olga Zagnitko, Irina Rodionova, Andrei Osterman, Adam Godzik

**Affiliations:** 1 Graduate School of Biomedical Sciences, Sanford-Burnham Medical Research Institute, La Jolla, California, United States of America; 2 Program on Bioinformatics and Systems Biology, Sanford-Burnham Medical Research Institute, La Jolla, California, United States of America; 3 Fellowship for Interpretation of Genomes, Burr Ridge, Illinois, United States of America; The Centre for Research and Technology, Hellas, Greece

## Abstract

Function diversification in large protein families is a major mechanism driving expansion of cellular networks, providing organisms with new metabolic capabilities and thus adding to their evolutionary success. However, our understanding of the evolutionary mechanisms of functional diversity in such families is very limited, which, among many other reasons, is due to the lack of functionally well-characterized sets of proteins. Here, using the FGGY carbohydrate kinase family as an example, we built a confidently annotated reference set (CARS) of proteins by propagating experimentally verified functional assignments to a limited number of homologous proteins that are supported by their genomic and functional contexts. Then, we analyzed, on both the phylogenetic and the molecular levels, the evolution of different functional specificities in this family. The results show that the different functions (substrate specificities) encoded by FGGY kinases have emerged only once in the evolutionary history following an apparently simple divergent evolutionary model. At the same time, on the molecular level, one isofunctional group (L-ribulokinase, AraB) evolved at least two independent solutions that employed distinct specificity-determining residues for the recognition of a same substrate (L-ribulose). Our analysis provides a detailed model of the evolution of the FGGY kinase family. It also shows that only combined molecular and phylogenetic approaches can help reconstruct a full picture of functional diversifications in such diverse families.

## Author Summary

The protein universe is under constant expansion and is reshaping through multiple duplication, gene losses, lateral gene transfers, and speciation events. Large and functionally heterogeneous protein families that evolve through these processes contain conserved motifs and structural scaffolds, yet their individual members often perform diverse functions. For this reason, the exact functional annotation for their individual members is difficult without detailed analysis of the family. In our study, we performed such a detailed analysis of a particularly heterogeneous FGGY kinase family through the integration of several computational approaches. The combination of phylogenetic and molecular approaches allowed us to precisely assign function to hundreds of proteins, thus reconstructing carbohydrate utilization pathways in almost 200 bacterial species. This analysis also showed that different molecular mechanisms could evolve within a group of isofunctional proteins. Moreover, based on our experience with this specific protein family of FGGY kinases, we believe that our approach can be generally adapted for the analyses of other protein families and that the accumulation of evolutionary models for various families would lead to a better understanding of the protein universe.

## Introduction

The large and functionally heterogeneous protein families that we see today result from long evolutionary processes with multiple duplications, gene losses, lateral gene transfers, and speciation events. The gene duplications usually leads to functional diversification within the family, for example, through the emergence of new catalytic mechanisms while preserving a common catalytic step as in the enolase superfamily [1], [2]. Even more common is the diversification of substrate preferences with the overall conservation of a catalytic mechanism [3] as in various amidohydrolases [4] and kinases [5]. It is generally agreed that new functional specificities emerge as a result of gene duplication and subsequent specialization, while they usually remain unchanged during speciation events [6]. In phylogenetic terms, functions tend to differ between paralogs and be conserved between orthologs, but the complex evolutionary history of most protein families, which includes also gene losses and lateral gene transfers, limits the application of purely phylogenetic approaches in interpreting function divergence. At the same time, other mechanisms, including convergent evolution of the same functions, are also possible. Among plausible evolutionary scenarios, a *simple divergent* model assumes the emergence of distinct functional specificities following duplication. In this scenario the same function is never invented twice, although it might become a subject of multiple gene losses and horizontal transfer events leading to mosaic phylogenetic distribution. *Mixed models* include instances of *convergent evolution* in which the same functional specificity is reinvented in distinct groups of species through lineage-specific expansions and specialization events. For example, the latter model was inferred for the evolution of some receptors in the innate immune system [7]. An extreme case of *convergent evolution of essentially identical functions from non-homologous solutions* is well documented in literature (for a recent review, see [8]). It is tempting to speculate that the same functional specificity would more readily reemerge (be reinvented) within the same family than between non-homologous families. Yet, whether such a phenomenon is indeed characteristic of functionally heterogeneous protein families remains an open question.

**Figure 1:**
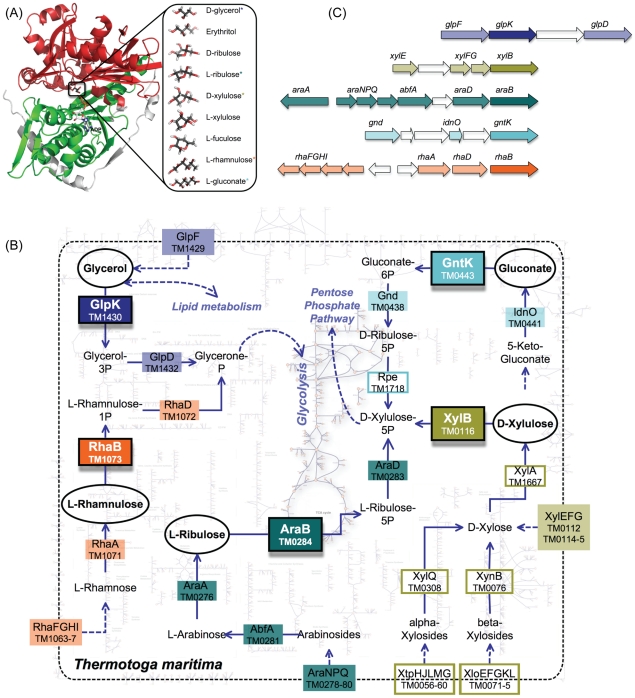
The three-dimensional structure, functional contexts, and genomic contexts of FGGY kinases. (A) The three-dimensional structure of a FGGY kinase (left) and a spectrum of substrates that may be utilized by this family (right). The FGGY_N and FGGY_C domains are colored red and green, respectively, shown in the example of a glycerol kinase (GlpK) from *E. coli* (PDB 1GLA). The co-product ADP was projected into the binding site from the structure of another FGGY protein (PDB 2UYT). The substrates that are utilized by *T. maritima* were marked with a star (*) with corresponding colors to the pathways in Figure 1B. (B) Overview of the metabolic pathways that employ FGGY kinases, shown in the example of a hyperthermophilic bacterium, *Thermotoga maritima*. The enzyme names and their encoding genes in the *T. maritima* genome are shown using respective background colors (as the shade or the frame) that indicate individual pathways, where the shaded boxes indicate genes in the genomic context of FGGY genes (shown in Figure 1C). The five FGGY kinases encoded in the *T. maritime* genome and their respective substrates are highlighted using black frames. Abbreviations: GlpF—glycerol uptake facilitator protein; GlpK—glycerol kinase; GlpD—glycerol-3-phosphate dehydrogenase; AraNPQ—alpha-arabinosides ABC transport system; AbfA—alpha-N-arabinofuranosidase; AraA—L-arabinose isomerase; AraB—L-ribulokinase; AraD—L-ribulose-5-phosphate-4-epimerase; RhaFGHI—rhamnose oligosaccharide ABC transporter; RhaA—L-rhamnose isomerase; RhaB—rhamnulokinase; RhaD—rhamnulose-1-phosphate aldolase; XtpHJLMG—Xylan oligosaccharide ABC transporter; XloEFGKL—Xylose oligosaccharides ABC transporter; XylEFG—Xylose ABC transporter; XynB—Beta-xylosidase; XylQ—alpha-xylosidase; XylA—Xylose isomerase; XylB—xylulose kinase; IdnO—gluconate 5-dehydrogenase; GntK—gluconokinase; Gnd—6-phosphogluconate dehydrogenase; Rpe—ribulose-phosphate-3-epimerase. (C) The genomic context of FGGY kinases in *T. maritima*. Each arrow indicates a gene in the *T. maritima* genome, and their relative positions indicate the distance between different genes.

Two major constraints that limit our ability to effectively address this question are the insufficient knowledge of the actual functions within such families and the limited accuracy of their evolutionary models. Indeed, experimental data about functional specificities are typically available for only a handful of representative proteins, and the homology-based annotation, available for other members of the family, is often imprecise (general class annotation such as *carbohydrate kinase*) or simply incorrect (misannotation) [9]. Likewise, the existing methods of evolutionary reconstruction based on sequence information alone often fail to disambiguate divergent branches on phylogenetic trees [10]. In this study we attempted to overcome both limitations by applying a combination of several complementary bioinformatic techniques. We tested this approach on a large protein family of FGGY carbohydrate kinases, which displays extensive variations in functional specificity: proteins in this family carry out ATP-dependent phosphorylation on one out of at least nine distinct sugar substrates (see Table 1 and the right panel of Figure 1A).

**Table 1:** The list of nine functions observed for the proteins in the FGGY kinase family.

Function	Abbr	EC number	Substrate	Product	Reference_proteins (Uniprot ID)	Cluster_IDs	References (PMID)	Structures (PDB ID)
L-ribulokinase	AraB	2.7.1.16	L-ribulose	L-Ribulose-5P	P94524, C4B4W2, P08204, P06188, Q9WYC0*	AraB_Clust11, AraB_Clust222, AraB_Clust228, AraB_Clust94	9084180, 19346355, 11747300, 2989100	3QDK
Erythritol kinase	EryA	2.7.1.27	Erythritol	D-Erythritol-4P	Q9ZB32	EryA_Clust85	12639570	
L-fuculokinase	FucK	2.7.1.51	L-fuculose	L-Fuculose-1P	P11553, Q04I07	FucK_Clust100, FucK_Clust124	3005235, 12618474	
Glycerol kinase	Glpk	2.7.1.30	D-Glycerol	D-Glycerol-1P	P18157, O34154, P0A6F3, P44400, Q9WX53, O66131, Q9NJP9, Q9X1E4*	GlpK_Clust115, GlpK_Clust22, GlpK_Clust265, GlpK_Clust309, GlpK_Clust76	2127799, 2545516, 9162046, 2826434, 11388799, 9972264, 9540790, 11154065	1BO5, 1BOT, 1BU6, 1BWF, 1GLA, 1GLB, 1GLC, 1GLD, 1GLE, 1GLF, 1GLJ, 1GLL, 1R59, 1XUP, 2DPN, 2W40, 2W41, 2ZF5, 3D7E, 3EZW, 3FLC, 3G25, 3GE1, 3H3N, 3H3O, 3H45, 3H46
Gluconokinase	GntK	2.7.1.12	D-gluconate	6P-D-Gluconate	P12011, Q9WYS4*	GntK_Clust13, GntK_Clust218	3011959	3GBT, 3LL3
L-xylulose kinase	LyxK	2.7.1.53	L-xylulose	L-Xylulose-5P	P37677	LyxK_Clust48	11741871, 7961955	
D-ribulokinase	RbtK	2.7.1.47	D-ribulose	D-Ribulose-5P	O52716	RbtK_Clust25	9639934	
Rhamnulo-kinase	RhaB	2.7.1.5	L-rhamnulose	L-Rhamnulose-1P	P32171, P27030, Q9X0G2*	RhaB_Clust137, RhaB_Clust95	2558952, 8396120, 1657713	2CGJ, 2CGK, 2CGL, 2UYT
Xylulose kinase	XylB	2.7.1.17	D-xylulose	D-Xylulose-5P	Q4JHR4, P09099, P29444, P35850, P21939, Q9P938, P27156, Q9WXX1*	XylB_Clust152, XylB_Clust252, XylB_Clust29, XylB_Clust342, XylB_Clust66, XylB_Clust87, XylB_Clust9	16834601, 6320721, 1324398, 9835554, 1660563, 11872473, 1657868	3I8B, 2ITM, 2NLX, 3IFR

**Titles of columns:** Function—the functional specificity of proteins; Abbr—the abbreviations of function; EC number—the Enzyme Commission number; Substrate/Product—the name of the substrates/products; Reference_proteins—the Uniprot accession numbers of the annotations supported by literature (functions determined in our laboratory are marked by *); Cluster_IDs—the numbering of the 30% sequence clusters in CARS; References—PubMed identification numbers of the reference publications; Structures—identifications of three-dimensional structures in the Protein Data Bank.

The choice of the FGGY family for this analysis was supported by several considerations. The broad cross-genome distribution of this family is illustrated by the identification of over 4,000 members in the NCBI Non-Redundant sequence database. A remarkable functional diversification of this family (mentioned above) was also emphasized in a recent review [8]. Three-dimensional structures of at least 44 members of this family have been solved, providing a solid overview of the structural divergence in this family. Known substrates of FGGY kinases include several distinct sugars ranging from trioses to heptoses. This family also contains a divergent subfamily functioning in quorum sensing, which phosphorylates AI-2, a bacterial signaling molecule derived from 4,5-dihydroxy-2,3-pentanedione (DPD) [11], [12]. The functional plasticity of FGGY kinases plays an important role in evolutionary diversity and adaptability of bacterial carbohydrate utilization machinery. Many bacterial genomes contain several representatives of the FGGY family with distinct specificities involved in catabolic pathways of different carbohydrates. For example, the *E. coli* and *B. subtilis* genomes each contain six FGGY kinases. Biological functions and biochemical substrate preferences of individual representatives of each specificity type were experimentally characterized, mostly for model species. For instance, in a recent study, substrate specificities of five FGGY kinases from the hyperthermophilic bacterium *Thermotoga maritima* were predicted and experimentally characterized (Rodionova *et al.*, unpublished) as an extension of our genome-scale reconstruction of a *T. maritima* metabolic network [13]. The metabolic network are used here to illustrate the diversity of FGGY kinases in the genomic and functional context of carbohydrate metabolism (Figures 1B, 1C). Notably, the divergent nature of three out of five *T. maritima* FGGY kinases would not have allowed a confident homology-based assignment of their substrate specificity (biological function). Indeed, in most public databases their annotations were typically limited by a general class function (e.g., “sugar kinase of FGGY family”). Likewise, incomplete and often incorrect functional assignments are widespread in public archives for thousands of FGGY kinases that are present in genomes beyond a handful of model organisms and their close relatives. This consideration was an additional motivation for the present analysis, which takes advantage of the well-defined biochemistry of carbohydrate utilization pathways and their known tendency to form conserved operons and regulons (Figures 1B, 1C) to efficiently use genomic and functional context as an important additional evidence for the functional assignment of associated enzymes. The application of such an approach (as recently illustrated [14]) in combination with literature information for the accurate functional classification of FGGY kinases was a key factor in building an extensive reference dataset, which enabled an evolutionary analysis reported in this study.

## Results

The comparative structural analysis of FGGY kinases reveals a number of highly conserved topological elements. All described members of this enzyme family are composed of two homologous actin-like ATPase domains. The two domains are named FGGY_N and FGGY_C, respectively (Figure 1A, left panel), using nomenclatures in the Pfam database [15], [16]. A catalytic cleft is formed by the interface between these two domains, where the sugar substrate and ATP co-substrate bind. Extensive structural and functional studies have been carried out on many members of the FGGY family, among them the glycerol kinase (EC:2.7.1.30, GlpK) and rhamnulokinase (EC:2.7.1.5, RhaB) from *E. coli* (Table 1). Analysis of experimentally determined three-dimensional structures have shown that the sugar substrate binds deeply within the catalytic cleft, forming interactions mainly with the N-terminus domain, whereas ATP binds near the opening, contacting both N- and C-terminus domains (Figure 1A, left panel). The binding of the sugar substrate drives a conformational change in which the two domains close to prevent solvent from entering the catalytic cleft [17], [18], [19]. Although some proteins appear to contain a single FGGY_N or FGGY_C domain, our analysis included only those conforming to the canonical two-domain architecture.

We have developed an FGGY kinase reference set starting from 31 representative enzymes with experimentally assigned substrate preferences and biological functions (including those verified by us using biochemical experiments; see Table 1). Although we focused our analyses on bacterial species, 3 out of the 31 representative enzymes are from eukaryotes, including 1 glycerol kinase (GlpK) from *Trypanosoma brucei* and 2 xylulose kinases (XylB) from *Pichia stipitis* and *Candida sp.* Xu316, respectively. We kept them in our dataset just to show that our analysis could potentially be extended to eukaryotic proteins. An expansion of this reference set to 446 proteins from a collection of fully sequenced bacterial genomes was based on two simultaneous requirements: (i) no less than 30% sequence identity to one of the reference proteins and (ii) conserved genomic (operons and regulons) and functional (pathways and subsystems) context [20], [21]. The latter type of requirement is rarely used in protein family analysis, as it is not easily amenable to automation. The use of SEED subsystems [22] allowed us to streamline identification of genomic neighbors of candidate FGGY kinases, as well as the presence or absence of certain “signature enzymes” of catabolic pathways, helping support or refute a considered functional assignment (see Supplemental Table S2 for the identified genomic and functional context with relevant functions). Although the 30% sequence identity threshold is lower than typically used for homology-based functional assignments, in our analysis it was strongly supported by the observed consistency with genomic and functional context. In further analysis we refer to this set of 446 FGGY kinases as a confidently annotated reference set (CARS). It is important to emphasize that our approach focused on the biological function of CARS enzymes, using their participation in specific biological pathways (Figure 1) as a key evidence for the functional assignment. In general, the relationship between biological function and biochemical substrate preferences of enzymes may be quite complex. However, our computational analysis and some experimental data argue in favor of rather good overall agreement between these characteristics in the FGGY kinase family (see below).

**Figure 2:**
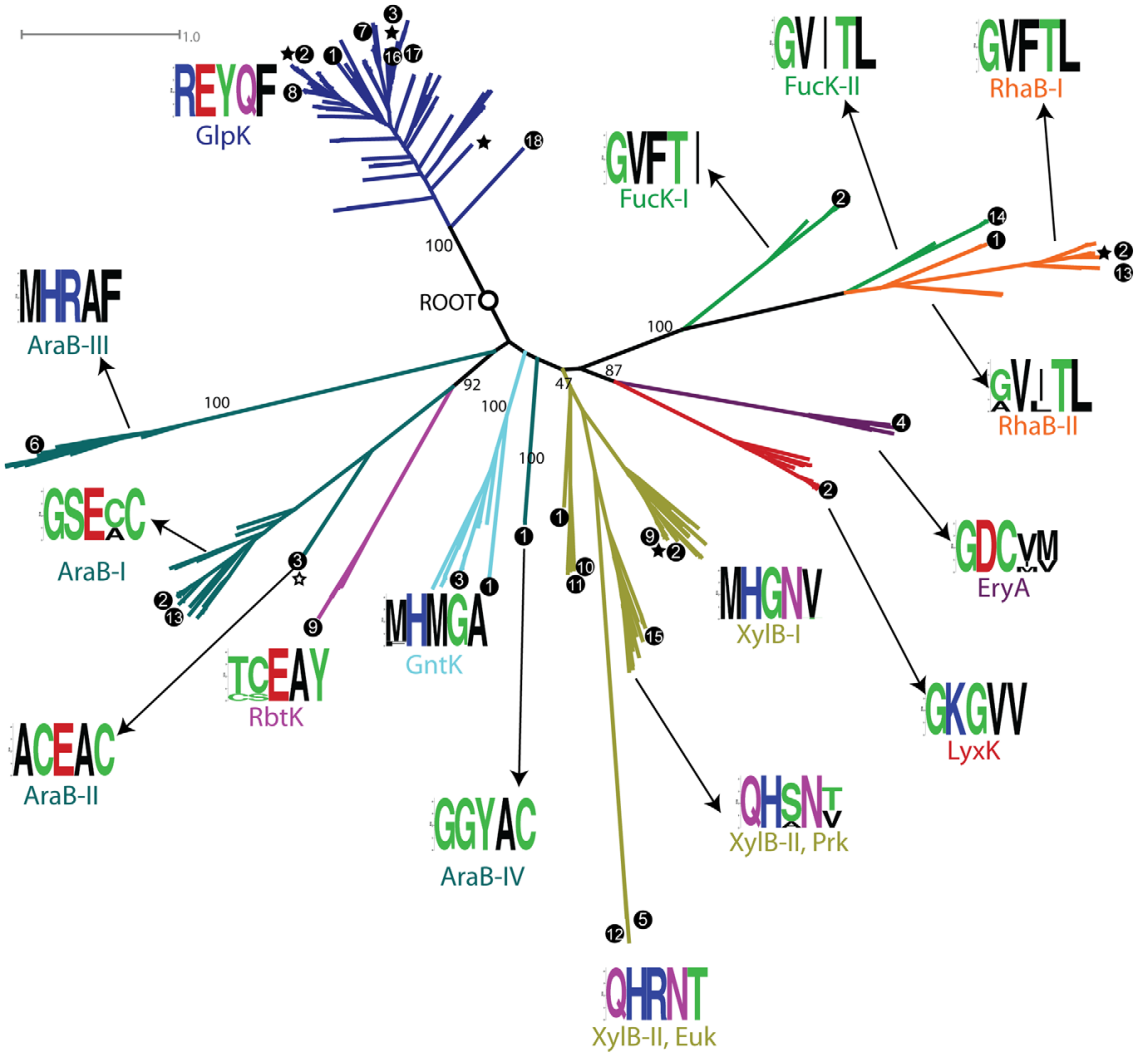
Phylogenetic tree of proteins from the annotated protein set CARS (a subset of the entire FGGY kinase family). The root was determined by using UvrC proteins (not shown) as an out-group, and the deep splits with a bootstrapping value higher than 40 are marked with numbers indicating their bootstrapping values. The branches are colored based on their functional specificities, and the color scheme is consistent with that used in Figure 1B. Some isofunctional groups were further divided into subgroups based on their positions in the tree, which is reflected in their labels. The amino acid distribution of specificity-determining positions (SDPs) are shown as logo characters produced by the Weblogo program [31]. The branches with three-dimensional structural information are marked with a star, and the branches supported by literature information are marked with a black dot. The numbers in the black dots indicate the different species from which FGGY proteins were identified: 1—*Thermotoga maritima*; 2—*Escherichia coli*; 3—*Bacillus subtilis*; 4—*Brucella abortus*; 5—*Candida* sp. Xu316; 6—*Corynebacterium glutamicum*; 7—*Enterococcus faecalis*; 8—*Haemophilus influenzae*; 9—*Klebsiella pneumoniae*; 10—*Lactobacillus brevis*; 11—*Lactobacillus pentosus*; 12—*Pichia stipitis*; 13—*Salmonella typhimurium*; 14—*Streptococcus pneumoniae*; 15—*Streptomyces rubiginosus*; 16—*Thermus aquaticus*; 17—*Thermus thermophilus*; 18—*Trypanosoma brucei*.

The confidently annotated reference set (CARS) of FGGY proteins included 9 distinct substrate specificities forming 25 clusters based on 30% sequence similarity clustering (Table 1). Some of the functions, such as glycerol kinase (GlpK), xylulose kinase (XylB), and L-ribulokinase (AraB), span multiple clusters. To identify cases of possible convergent evolution of substrate specificities, we built a phylogenetic tree of all the CARS proteins (Figure 2). The leaves of the tree represent individual proteins in the reference set (Supplemental Table S1), and their leading branches were colored according to the functions of the leaves. As depicted in Figure 2, most proteins form tight clusters with uniform function. This favors the divergent model, suggesting a common ancestor for all enzymes of the same substrate specificity. For example, the largest group in our dataset, the glycerol kinase (GlpK, colored blue in Figure 2) group, although coming from five different sequence clusters, forms a single large branch in the phylogenetic tree, pointing to its ancestral nature and its single origin. Some outliers, however, have been observed for a few other FGGY kinase functions. The L-ribulokinase (AraB, colored dark cyan in Figure 2) group appears to split into distinct branches interspersed by D-ribulokinase (RbtK) and gluconokinase (GntK) branches. This observation, in principle, opens a possibility of a mixed model with elements of convergent or parallel evolution of substrate specificities in some sub-branches.

To explore such ambiguous cases and elucidate the molecular-level evolutionary history of the FGGY family, we used an additional approach aimed at identifying signature amino acid residue positions that are responsible for the recognition of a specific substrate. Ideally, these so-called specificity-determining (or signature) positions (SDPs) should indicate columns in the multiple sequence alignment of a family that are conserved within an isofunctional subgroup of proteins while different between subgroups with distinct specificities. Many tools have been developed for the prediction of SDPs, using a variety of techniques that take into account the sequence, structural, and phylogenetic information of a protein family [23], [24], [25], [26], [27], [28]. The SDPpred algorithm [25], which we adopted for the purpose of our analysis, is based solely on the statistical analysis of a multiple sequence alignment. It determines a ranking of alignment columns based on the assumption that proteins in the same group use a similar molecular mechanism, i.e., conserved amino acid residues, to carry out specific functions. In our analysis we combined the predicted ranking of SDPs with protein structural information to define signature residues (see Materials and Methods for details). Since some functional subgroups in our dataset span multiple low-identity sequence-based clusters, we first applied SDPpred to a collection of the largest clusters from each specific group and then mapped the predicted ranking of SDPs into all other clusters through a “master” multiple sequence alignment to allow the comparison of molecular mechanisms among different clusters within an isofunctional group (Supplemental Figure S1). Of the three-dimensional structures in the Protein Data Bank [29], 44 structures representing 17 distinct proteins were mapped to the FGGY family based on database search using Hidden Markov Models provided by Pfam database version 24.0 [30]. As a result, five SDPs were selected, from which signature residues were defined for each CARS protein, and a signature that reflects the amino acid distribution in the five SDPs was determined for each compact branch on the phylogenetic tree. Below, we will show detailed analyses of these positions and their amino acid distributions.

**Figure 3:**
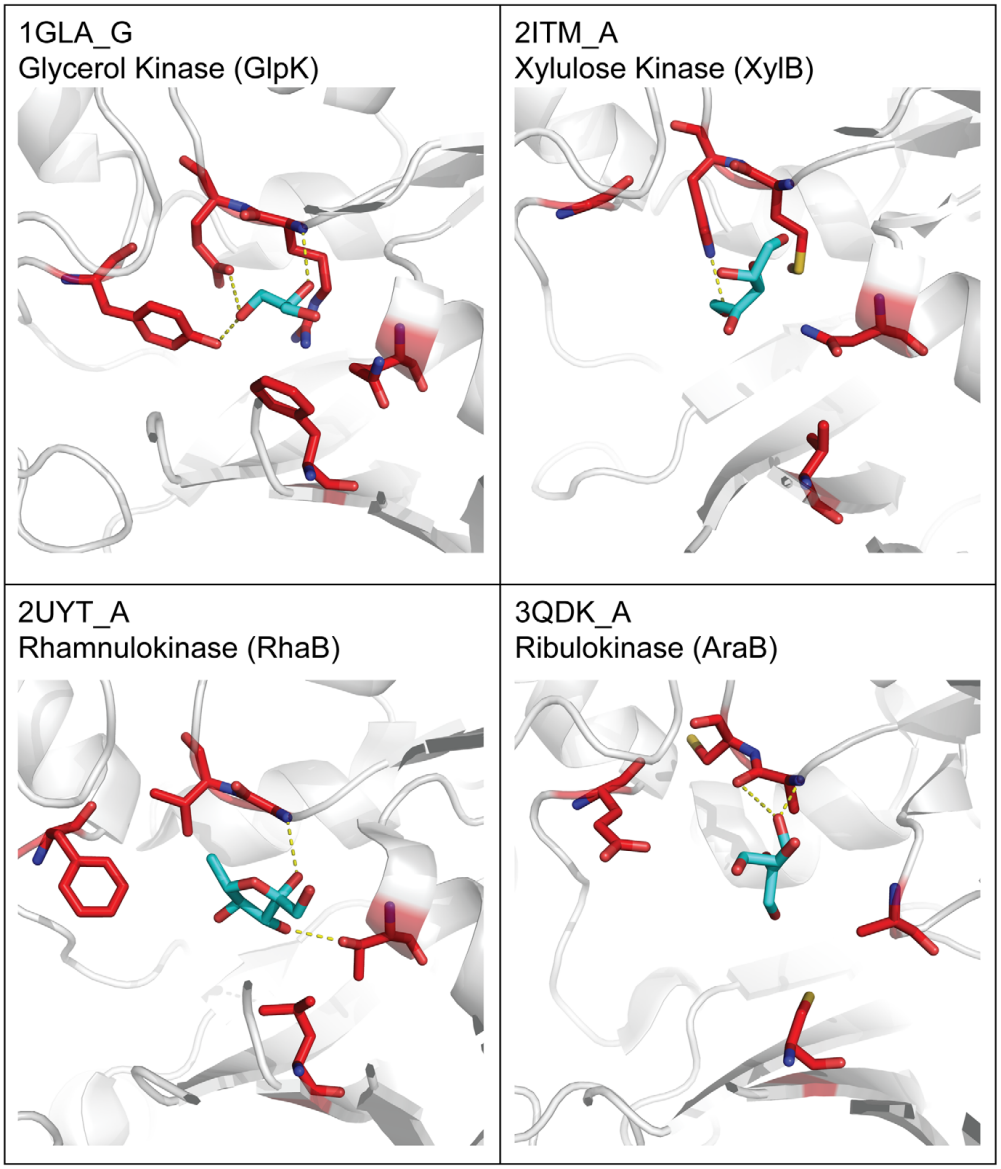
Position of signature residues in the three-dimensional structures of FGGY kinases. Labels in each cell show the protein and chain identification numbers from the Protein Data Bank [29], as well as the protein function. The protein backbone is shown in white cartoon representation, the signature residues are marked with red sticks, and the co-crystallized substrates are marked as cyan sticks.

**Figure 4:**
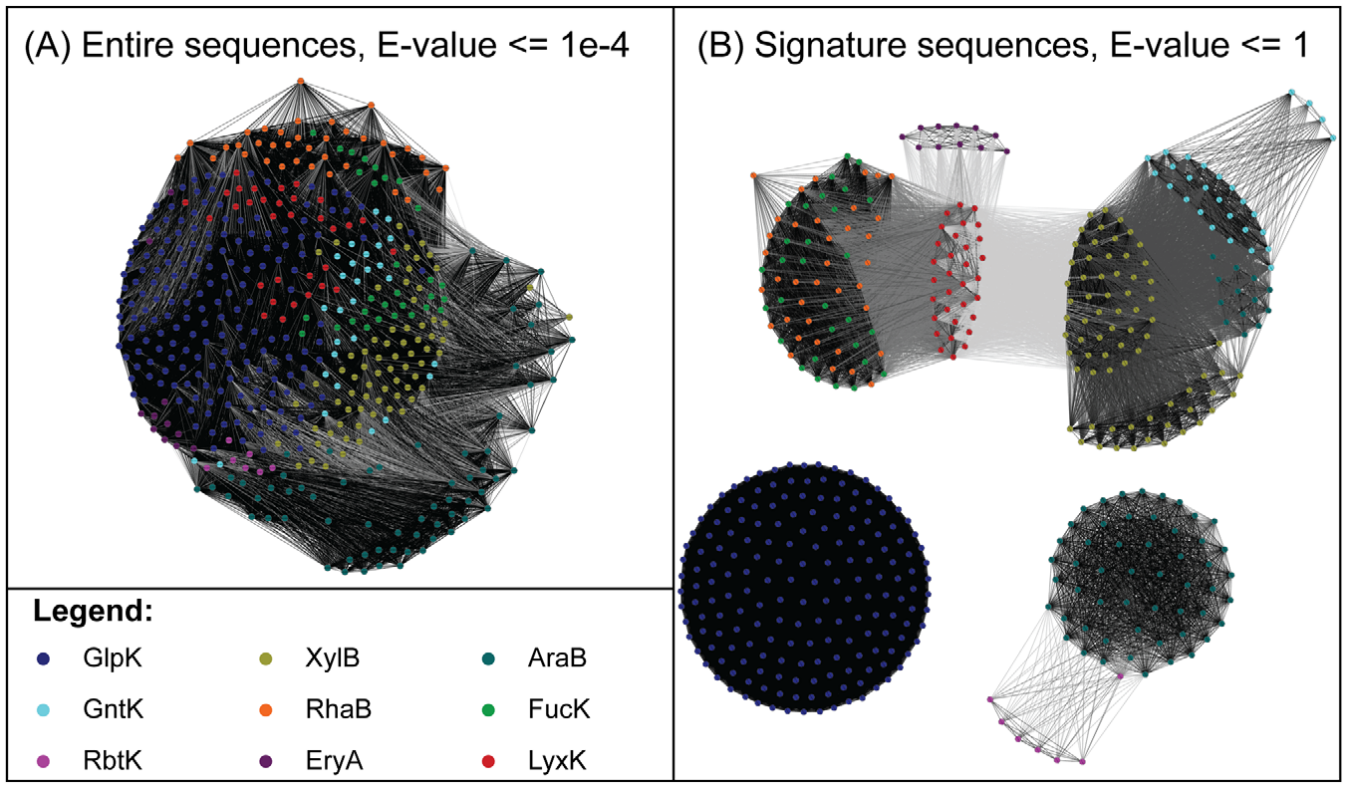
Protein similarity networks reconstructed based on entire sequences (A), as well as signature sequences (B), for all FGGY proteins in CARS. Each node in the network represents a single protein in the annotated dataset, and each edge represents a BLAST alignment with an E-value better then a given threshold indicated in the graph. The nodes are colored according to their functions (see Legend—color scheme is the same as in Figure 2). The edges are colored in a gray scale: the darker the color is, the more significant the similarity is. The nodes were arranged using the yFiles organic layout provided with Cytoscape version 2.7 [47].

The five selected SDPs, while distant from each other in sequence, are located in the vicinity of the active site holding approximately the same positions in three-dimensional structures of representative FGGY kinases from distinct isofunctional groups (Figure 3). Side chains of the signature residues in these positions tend to point toward the center of substrate-binding sites, forming interactions with the substrates. For example, among the five signature residues of glycerol kinase (GlpK), four (Arg83, Glu84, Tyr135, and Asp245) form hydrogen bonds with the hydroxyl groups of glycerol and one (Phe270) is in van der Waals contact with the carbon backbone of glycerol [15]. An additional validation of the predicted signature positions was provided by the observation that the SDP-derived signature sequences (a concatenated sequence of signature residues) would allow functional classification of FGGY kinases, splitting the entire FGGY protein space into tightly clustered and largely mono-functional groups. This is illustrated by the protein similarity networks (PSNs) reconstructed based on the sequence signatures (Figure 4). Furthermore, a comparison of the PSN built on sequence signatures with the PSN built on the entire sequences, under various thresholds of sequence alignment E-values (Supplemental Text S3), showed that the functional information encoded in the entire sequences is preserved in the five signature positions, adding to the functional relevance of signature residues. These observations strongly argue that the residues in the identified SDPs play important roles in substrate recognition and the determination of functional specificities.

The amino acid distributions on SDPs form a signature of each specific isofunctional group (and their subgroups) and are shown in Figure 2 as consensus logos made with the Weblogo [31] software. In addition, the SDP signatures can also be represented as position weight matrices, and they were compared using a hierarchical clustering approach based on the similarity of signature pairs (Supplemental Text S2). The SDP signature of glycerol kinases (GlpKs), in addition to their tight branching in the phylogenetic tree, confirmed that they all came from the same origin. Although members of the GlpK group have largely variable global sequences forming five distinct clusters of less than 30% sequence identity, among which only one cluster was selected for sequence-based SDP prediction and others had their SDPs mapped from a “master” multiple sequence alignment of all CARS proteins, their signature residues are extremely well conserved and identical among different clusters. In the middle of the spectrum, the xylulose kinase (XylB) group was divided into three branches in the phylogenetic tree: XylB-I contains four clusters and is the most dominant form of the xylulose kinases, while XylB-II, which contains three clusters, was divided into prokaryotic branches (XylB-II, Prk) and eukaryotic branches (XylB-II, Euk). The signature motifs of all three clusters are very similar (Supplemental Figure S3), with the prokaryotic branches of XylB-II having the most variable amino acid distributions acting as a transition between XylB-I and the eukaryotic branches of XylB-II. This is also reflected in the relative positions of XylB-I and the two XylB-II branches in the phylogenetic tree. In a more extreme case, the L-ribulokinase (AraB) group has the least conserved signature residues. This group was divided into four branches—AraB-I, -II, -III, and -IV—on the phylogenetic tree. Indeed, they all have distinct signature motifs, of which three (AraB-I, -II, and -IV) are more similar than the fourth (AraB-III) (Supplemental Figure S3). Although the signature of AraB-III is more distant from other L-ribulokinases (AraBs), this branch is closer to AraB-I and -II on the phylogenetic tree, whereas AraB-IV, whose signature is closer to AraB-I and -II (Supplemental Figure S3), is more distant from other L-ribulokinases (AraBs) on the tree. This observation favors the simple divergent model in the evolution of L-ribulokinases (AraBs), in which a common ancestor of all L-ribulokinases (AraBs) diverged within the same isofunctional group, followed by the emergence of at least two distinct biochemical mechanisms for the implementation of the AraB function. The D-ribulokinase (RbtK) and gluconokinase (GntK) branches, which independently emerged from AraB-II and AraB-III, carry signatures that are similar to these two subgroups, respectively, indicating that they adopted the different signature residues and evolved from the two subgroups of L-ribulokinase (AraB). An additional case of interest was the L-fuculokinase (FucK) and rhamnulokinase (RhaB) groups. They carry very similar signatures (Supplemental Figure S3) and form a poorly resolved single cluster on the phylogenetic tree (shown as green and orange in Figure 2), suggesting these two functions not only are evolutionarily related, but also have employed similar biochemical mechanisms.

**Figure 5:**
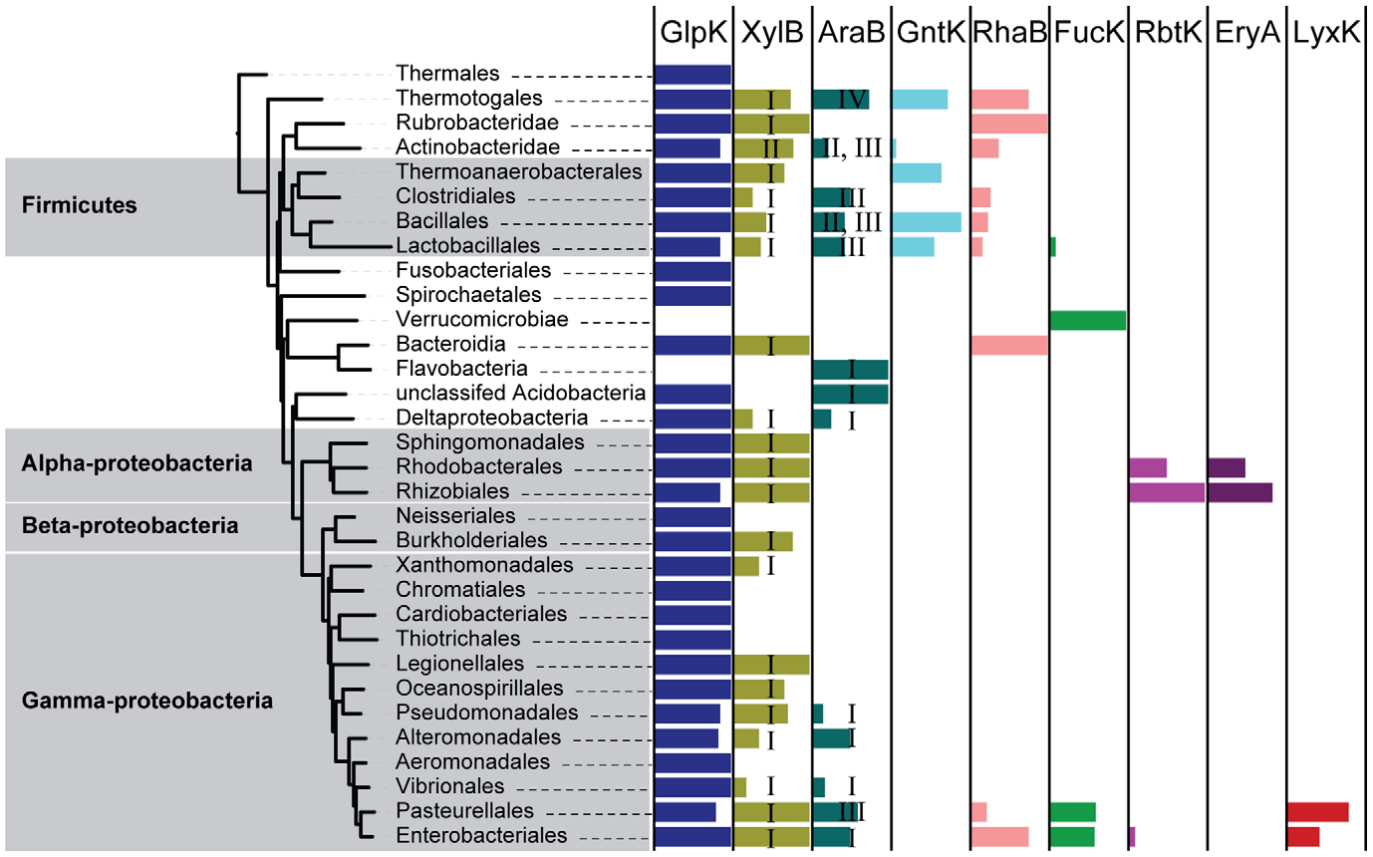
Distribution of FGGY kinase functions in a species tree extracted from a published tree of bacterial species [**32**]. Branches in the species tree were collapsed to show the higher taxonomic level. The colored rectangle bars (color scheme is the same as in Figure 2) on the right shows the functional distributions of each taxonomic group. The width of the bar indicates the proportion of species within a taxonomic group containing a specific function. The XylB and AraB groups are numbered according to the divisions in the protein tree to show their evolutionary patterns. The original layout was made with the Web interface of iTol [49] and was proportionally scaled and manually labeled. All rectangle bars were zoomed in proportionally so that they could be highlighted in the graph.

In addition to assisting the evolutionary analysis, identification of SDPs helped in accurate propagation of functional assignments. Thus, we used a combination of context-based and signature-based analyses (see Materials and Methods for details) to expand annotations of all 9 specificities over the entire set of 191 completely sequenced bacterial genomes comprising the original CARS. As a result, functional assignments were made for 785 additional proteins from these complete genomes based on the consensus between signature residues and the genomic context of newly annotated FGGY kinases. This expansion allowed us to analyze the taxonomic distribution of FGGY kinase functions, illustrated here by a projection over a species tree (a subtree derived from [32], see Materials and Methods for more details) (Figure 5). Comparative analyses of the protein and species trees (as in Figures 2 and 5) are commonly used for detailed evolutionary reconstructions of functionally heterogeneous protein families [33], [34], [35].

The obtained results showed that glycerol kinase (GlpK) is the most dominant isofunctional group that exists in nearly all species. As another extreme, D-ribulokinase (RbtK) and erythritol kinase (EryA) appear exclusively in Alphaproteobacteria, whereas L-xylulose kinase (LyxK) exists only in a few groups of Gammaproteobacteria. The taxonomic distributions of the various subgroups of L-ribulokinase (AraB-I, -II, -III, -IV) and xylulose kinases (XylB-I, -II) were indicated with roman numerals on the species tree. In the case of AraB, while the most abundant group, AraB-I, is spread broadly among various phyla, AraB-III and AraB-IV are contained within the two lineages of Firmicutes and Thermotogales, respectively. Similarly, XylB-I is the dominant case, whereas XylB-II is confined only in Actinobacteridae. Notably, the FGGY kinase family experienced several instances of lineage-specific expansion, for example, in Pasteurellales and Enterobacteriales, leading to an extensive repertoire of six FGGY kinases including a “newborn” L-xylulose kinase (LyxK) function. Although the analysis of lateral gene transfer events was beyond the scope of this study, it apparently played a substantial role (together with massive lineage-specific gene losses) in shaping up the observed mosaic distribution of several FGGY kinase functions. For example, the AraB-III subgroup was shared by species out of the Firmicutes lineage (Actinobacteridae and Pasteurellales), while the AraB-II subgroup appeared in some Firmicutes.

## Discussion

A majority of proteins in the protein universe belong to a relatively small number of large protein families [6]. In evolution, large protein families have expanded through duplications and subsequent specializations within single genomes, leading to the emergence of new (but usually similar) functions within the same family. However, little is known about how different functions emerge in protein families and how the emergence of such functions happens on the molecular level. Here, in the example of the FGGY carbohydrate kinase family, we assessed contributions of divergent and convergent evolution of functions in the history of this family. Such analyses could not have been done even a few years ago because of the lack of experimental data, three-dimensional structural information, and sufficiently large sets of functionally assigned protein sequences from a wide variety of organisms. In order to reconstruct the evolutionary history of FGGY kinases, we built a reference set of proteins with accurate annotations of biological functions (CARS) based on experimental data and context-based functional predictions. We then expanded it by combining predictions based on genomic and functional context analysis with predictions based on signature residues. This allowed us to study the evolution of substrate specificity in the FGGY kinase family on both phylogenetic and molecular levels, combining a protein phylogenetic tree, a species tree, and signature residues.

To determine specificity-determining positions (SDPs), we used a modified integrative approach combining the sequence-based algorithms [25], [26], [36] with the structure-based determination of functionally important residues [15] using three-dimensional structures of representative FGGY kinases co-crystallized with their natural substrates. The consensus of both helped eliminate false-positive predictions and narrowed our search to positions that are essential for molecular binding rather than for general catalysis (Supplemental Figure S2). The identified SDPs were highly useful in the evolutionary analysis of FGGY kinases and provided consistent functional assignments for both the CARS proteins (Figure 4B) and the new proteins in a number of complete genomes. They also gave remarkable insights into the structural basis and biochemical mechanism of specific substrate recognition in the FGGY family. The molecular signatures of SDPs, however, have certain limitations in discriminating some functional groups. For example, the signatures of the L-fuculokinase (FucK) and rhamnulokinase (RhaB) groups are very similar to each other (Supplemental Figure S3), and these two functional groups form a poorly resolved single branch in the phylogenetic tree (Figure 2). In this case it is tempting to speculate that the observed similarity at their overall sequence and signature levels reflects the chemical similarity of the stereoisomeric substrates, L-fuculose and L-rhamnulose, but at the same time we should keep in mind that this does not hold true for all stereoisomers. For instance, the similarity between SDP signatures of D- and L-xylulose kinases, or D- and L-ribulokinases, is less obvious.

The construction of protein phylogenetic trees is a common approach for the evolutionary study of protein families. In the case of the FGGY family, phylogenetic analyses revealed a single origin for eight out of nine studied isofunctional groups. However, different L-ribulokinase (AraB) branches were seen on the phylogenetic tree, suggesting two possible evolutionary scenarios: (i) these branches represent decedents of distinct ancestor proteins or (ii) these branches represent an early divergence of a common ancestor. The former would support the mixed model, in which the same function was “invented” independently more than once. The latter scenario would support a simple divergent model. A likely solution of the AraB conundrum was obtained by combining the location of different branches on the phylogenetic tree with the molecular-level analysis of signature residues. The signatures of AraB-III and GntK contain successive methionine and histidine residues at the first two positions, which also appeared in the XylB-I cluster, suggesting a divergent evolution event that created the GntK function from AraB-III. The same is true for RbtK, which emerged from the AraB-II branch following the divergence of their molecular binding sites. Therefore, the latter evolutionary scenario (the simple divergent model) is favored, and it is quite likely that the D-ribulokinase (RbtK), gluconokinase (GntK), and L-ribulokinase (AraB) functions all emerged from a common ancestor but followed distinct molecular-level solutions. The evolution of multiple molecular solutions for the L-ribulokinase function reflected the plasticity of enzyme active sites as described by Todd *et al.* [37].

**Figure 6:**
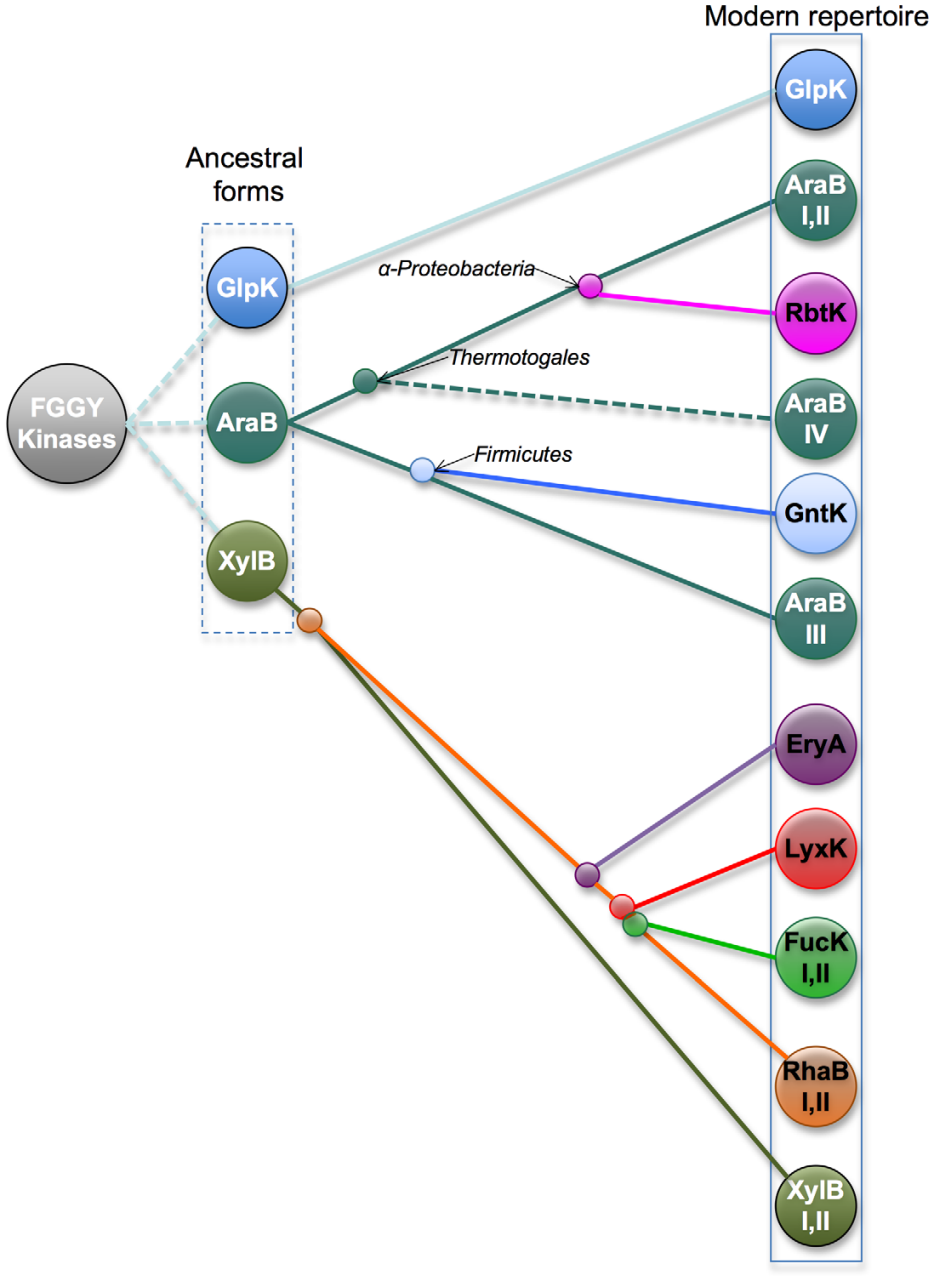
The proposed evolutionary model of the FGGY kinase family.

The combined analyses of a protein phylogenetic tree and a species tree allowed a complete evolutionary reconstruction of the FGGY kinase family (Figure 6). The glycerol kinase (GlpK), L-ribulokinase (AraB), and xylulose kinase (XylB) groups are the most dominant isofunctional groups that exist in the majority of bacterial species. As inferred by both the protein and the species trees, they are probably the ancestral forms of FGGY kinases in bacteria. While GlpK remained unchanged in its functional specificity and molecular mechanism, AraB and XylB diverged to form distinct biochemical mechanisms within the same function or to form new functional groups. In the case of AraB, at least two distinct solutions have emerged for the recognition of L-ribulose substrate with the splitting of Thermotogales and Firmicutes species. Additionally, an ancestral AraB apparently gave rise to two distinct functions, gluconokinase (GntK) and D-ribulokinase (RbtK). Four distinct groups of diverse specificities appear to have evolved within the XylB branch, and among them is a relatively recent divergence of rhamnulokinase and L-fucolokinase functions. The GlpK group stayed remarkably unchanged during evolution, exhibiting a high level of conservation of signature residues compared to other isofunctional groups. This observation may be rationalized by considering at least two types of constraints. First of all, GlpK plays a unique role, which extends beyond carbohydrate catabolism and links to the lipid metabolism, in the central metabolic network of all bacterial species (Figure 1B). This is consistent with the ancestral origin and broad conservation of GlpK across the species tree (Figure 5). Second, glycerol is the smallest (three-carbon) in the entire panel of substrates of FGGY kinases (Figure 1A, right panel), which may provide another set of constraints on active site variations while preserving optimal affinity and specificity.

The functional assignments in our dataset were based on the reconstruction of genomic and functional context and reflect the biological functions of proteins. The signature residues, on the other hand, should in principle reflect the biochemical specificities of proteins regardless of their biological context. The fact that we can extract compact biochemical signatures from the isofunctional groups annotated through biological-context analyses suggests that there is good agreement and relatively little promiscuity between biological function and biochemical specificity. In fact, experimental data available so far suggest a typically narrow specificity of FGGY kinases to the preferred physiologically relevant substrate. Our experiments on all five *T. maritima* FGGY kinases showed non-overlapping substrate specificity profiles, in each tested case showing over tenfold preference for the respective physiological substrate (Rodionova *et al.*, unpublished). Therefore, members of the FGGY family are characterized by their specific functions that are mediated by a small number of specificity-determining residues. A notable exception is the rhamnulokinase (RhaB) and L-fucolokinase (FucK) functions. Our computational analyses on the protein phylogenetic tree and signature residues suggested that they might have mixed specificities to both substrates (L-rhamnulose and L-fucolose), and biochemical experiments also confirmed that an *E. coli* rhamnulokinase has a spectrum of potential substrates that includes L-rhamnulose, L-fuculose, and L-xylulose [38], [39]. This biochemical promiscuity can also be explained by our evolutionary model, in which the functions of L-fuculokinase (FucK), L-xylulose kinase (LyxK), and erythritol kinase (EryA) emerged relatively recently from rhamnulokinase (RhaB).

Finally, our study provides a workflow that can be efficiently used for the functional and evolutionary analysis of large and functionally heterogeneous protein families. Based on our experience with the specific protein family of FGGY kinases, we believe that this approach can be generally adapted for the analyses of other protein families. Specifically, this workflow can be useful in building an initial set of high-quality annotations to allow the application of other high-throughput approaches for the identification and analyses of isofunctional subfamilies [33], [40], [41].

## Materials and Methods

### Preparation of the annotated protein set

The confidently annotated reference set (CARS) of proteins was created through expanding a literature-based reference set of 31 proteins (Table 1) based on sequence similarity and comparative genomic approaches. Specifically, we required that the annotated proteins should have at least 30% sequence similarity to a reference protein and that the annotation should be supported by the genomic and functional context [20]. The 30% sequence clustering was achieved using the UCLUST method implemented in the USEARCH package [42]. We used the SEED annotation and analysis tool [22] to collect information about the genomic and functional context of proteins. A subsystem was built for the nine reported functions of FGGY kinases and their adjacent functions in the respective metabolic pathways, and then all the complete genomes in the RAST server [43] were mapped to the subsystem to check for the functional annotation of FGGY kinases and their neighbors in the genomes. The functional assignment of a specific protein is confirmed and included into CARS only when it has genomic neighbors that perform relevant functions in a metabolic pathway.

### Prediction of signature residues

The signature residues in our analyses were identified based on two criteria. First, the residues should be significantly conserved within a subgroup of proteins of identical substrate specificity, and they should be distinct among different subgroups. Second, the residues should locate within a certain range of a co-crystallized substrate in the three-dimensional structure. The first criterion was achieved using the SDPpred server [25], which requires as input a multiple sequence alignment (MSA) and a grouping of proteins based on their specificity. In this case, the MSA of all CARS proteins was built with the MUSCLE program [44], and we used a modified procedure to better accommodate the algorithm of SDPpred (Supplemental Text S1). The result of SDPpred is a list of rankings for each individual alignment position, and the ranking indicates the significance of the position in distinguishing different isofunctional groups. The second criterion was achieved by calculating the average distance from a residue position to the substrate. The residues were mapped from structures to the MSA so that an average distance could be calculated for each alignment position. The average distances of the residues to the substrates were plotted against the SDPpred ranking of residue positions to show the overall trend of these two parameters (Supplemental Figure S2). Five positions were selected from the MSA using the following threshold: the ranking should be better than the global minimum of a Bernoulli estimator, and the average distance to co-crystallized, functionally relevant ligands in the structured proteins should be no more than 4 Å. The false-positive predictions using the sequence-based SDPpred algorithm alone were indicated with filled black dots, and those using structure-based information alone were shown in a dashed square in the upper left of Figure S2. The consensus of both helped to eliminate these false-positive predictions.

### Reconstruction of protein similarity networks

The protein similarity networks (PSNs) were built using the method described in [45], and the same approach was used to build PSNs for both signatures and entire sequences. First, alignments were obtained for each pair of sequences using the “blast2seq” program from the NCBI toolkit [46] with a parameter of word-size equal to 1. The program returns an E-value for each pairwise alignment, indicating its significance. Second, a threshold is chosen for the selection of sequence pairs that are significantly similar (with an E-value better than the cutoff value). Finally, a network is built based on the pairwise E-values and the selected threshold. Each node in the network indicates a protein in CARS, and each edge in the network indicates that the pair of nodes linked by this edge has an alignment with an E-value more significant than the selected threshold. The network was visualized using Cytoscape software version 2.7 [47], and the nodes in the network were arranged using the yFiles organic layout method.

### Similarity and hierarchical clustering of signatures

Position weight matrices (PWMs) were built for the annotated protein set on each 30% sequence cluster. The matrices each have 20 rows and 5 columns, indicating the distribution of 20 amino acids on the 5 signature positions. Pairs of signatures (in the form of PWMs) were compared based on the correlation coefficients calculated with the corr2 function in MATLAB. Based on the correlation coefficients, signatures of different protein clusters were grouped using hierarchical clustering implemented in the hclust function in the R software package. More details on how to evaluate the similarity of PWMs and how to perform hierarchical clustering of signatures are in the Supplemental Text S2.

### Phylogenetic trees

A protein phylogenetic tree (Figure 2) was built on the CARS proteins with the FastTree program [48] using default parameters. The root (marked as a black circle) of the protein tree was determined using the endonuclease subunit of excinuclease ABC (UvrC) as an out-group. The bootstrapping values on the tree were computed with the consensus of 100 random trees using the “Fast Tree-Comparison Tools” provided with the FastTree program (scaled so that 100 is the maximum), and the random trees were calculated with the “-n” option of the FastTree program [48] over a list of bootstrapped sequences generated from the original sequence alignment using the SEQBOOT program in the PHYLIP package.

The species tree was extracted from a published tree of bacterial species built on a concatenated alignment of a number of marker genes [32]. When extracting the species tree, we only considered species whose genomes were completely sequenced, and we only used this tree to determine location of a species, but not specific strains. In Figure 5, branches were collapsed to show an overview of the more general taxonomic level, and this general taxonomic annotation was shown as leaves of the tree. A complete expansion of all species and their functional predictions in the species tree is listed in Supplemental Table S3. The functional distribution view in Figure 5 was prepared with the support of the iTol interface [49]. The width of the colored bars is proportional to the ratio between the number of species that contain a protein of given function and the total number of species examined in a taxonomic group.

### Full genome survey of FGGY kinases in selected bacterial species

In order to remove biases given by false-negative annotations in the species tree analyses, we expanded the training set (only for the species tree analysis) through full genomic surveys of all species in the annotated set. All the predicted FGGY functions (Supplemental Table S3) were based on genomic and functional context prediction, as well as the identity of signature residues. Functional assignments were made when consistencies were reached between the two criteria and were added to the functional spectrum views in Figure 5.

## Supporting Information

Figure S1 **Sequence-based prediction of specificity-determining positions (SDPs).** When the level of sequence similarity within isofunctional groups is low, a sequence-based protein clustering is needed to identify a list of conserved isofunctional clusters for the application of standard SDP prediction algorithms. The predicted SDPs can then be mapped to the rest of the proteins using a master alignment of the entire family. The amino acid distributions among different clusters within the same isofunctional group can then be compared to identify same or distinct chemical mechanisms. F1 and F2 indicate two different isofunctional groups within a family. C1, C2, C3, and C4 are conserved isofunctional clusters, among which C1 and C3 were selected in SDP prediction to represent F1 and F2, respectively.
(TIF)

Figure S2 **Selection of SDPs in a multiple sequence alignment (MSA) of FGGY kinases combining sequence and structural information.** The SDPpred ranking of MSA positions were plotted against their average distances from co-crystallized, functionally relevant ligands in three-dimensional structures. Each data point represents an alignment position. The open circles are all positions in the MSA that have an SDPpred ranking and an average distance value. The filled black dots indicate MSA positions with an SDPpred ranking higher than the global minimum. The red circles indicate the five signature residue positions with and SDPpred ranking higher than the global minimum and the average distance no more than 4 Å.
(TIF)

Figure S3 **Comparison of SDP signatures among clusters of isofunctional proteins.** (A) A heat map based on the correlation coefficients of the signature position weight matrices. The heat map is symmetric, with identical row and column labels. Each cell in the heat map contains the correlation coefficient of two signatures indicated by their column and row labels. The heat map is color coded so that brown indicates higher and blue indicates lower correlation coefficient values. (B) An enlarged version of the similarity tree in the heat map. The tree was built based on a hierarchical clustering approach implemented in the hclust tool in the R software package. Red boxes indicate the global-level clustering (based on signature identity) of the protein clusters.
(TIF)

Table S1 **List of proteins in the confidently annotated reference set (CARS) with their annotations based on literature and/or context-based analyses.** Uniprot_Acc: the Uniprot accession number of proteins; SEED_PEGid: the protein identification number in SEED; Uniprot_Recname: the Uniprot-recommended name of the protein; Uniprot_Subname: the submitter-recommended name of the protein; Organism: organism name from which the protein was identified; Context-based annotation: the annotation based on genomic and functional context analyses; Functional_context: the number of proteins in the genome that perform neighboring functions in a metabolic pathway of the target protein; Genomic_context: the number of proteins in a same operon of the target protein that perform neighboring functions in the metabolic pathway; Reference_annotation: the annotation based on literature; Clust_ID: the cluster numbers of the target proteins in 30% sequence identity clustering.
(PDF)

Table S2 **Genomic and functional context of proteins in the confidently annotated reference set (CARS).** Uniprot_Acc: the Uniprot accession number of proteins; SEED_PEGid: the protein identification number in SEED; Context-based annotation: the annotation based on genomic and functional context analyses; Functional_context: the number of proteins in the genome that perform neighboring functions in a metabolic pathway of the target protein (same as in Table S1); Functional_context_PEGids: the SEED protein identification numbers of the functional context; Genomic_context (same as in Table S1): the number of proteins in a same operon of the target protein that perform neighboring functions in the metabolic pathway; Genomic_context_PEGids: the SEED protein identification numbers of the genomic context.
(PDF)

Table S3 **Prediction of FGGY kinase functions in a list of selected genomes based on genomic and functional context prediction, as well as the identity of signature residues.** Taxonomy_ID: the taxonomy identification numbers of the species; Species (Strain): the name and strain number of the species; Uniprot_list: the list of FGGY proteins identified by their Uniprot Accession numbers; Taxonomy_level: the taxonomy levels chosen in Figure 5 to collapse the species tree; Species: the name of species without counting strain names (when calculating the existence of various FGGY functions in the species tree, the counts were averaged among various strains of the same species, which is represented only once on the tree); Functions: the existence of various FGGY functions (see Table 1 for the full names of different functions), a number larger than 0 indicates a function exists in the genome, “0” indicates a function does not exist in the genome.
(PDF)

Text S1 **The prediction of SDPs, combining sequence and structural information.**
(DOC)

Text S2 **The hierarchical clustering of SDP signatures.**
(DOC)

Text S3 **Reconstruction and analysis of protein similarity networks.**
(DOC)
