# Supplementary figures and images for: The FGGY Carbohydrate Kinase Family: Insights into the Evolution of Functional Specificities
Source: PLoS Comput Biol. 2011 Dec 22;7(12):e1002318. doi: 10.1371/journal.pcbi.1002318 (PMC3245297; doi:10.1371/journal.pcbi.1002318)

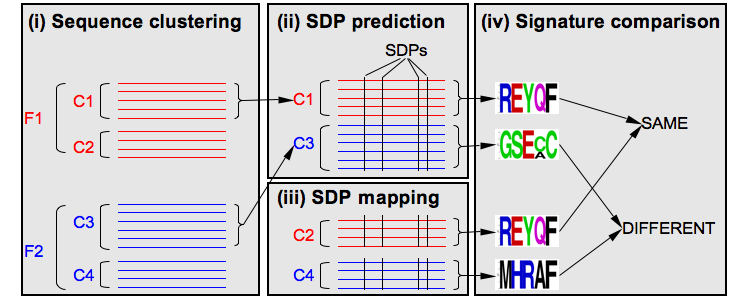

Supplement: Figure S1 — Sequence-based prediction of specificity-determining positions (SDPs). When the level of sequence similarity within isofunctional groups is low, a sequence-based protein clustering is needed to identify a list of conserved isofunctional clusters for the application of standard SDP prediction algorithms. The predicted SDPs can then be mapped to the rest of the proteins using a master alignment of the entire family. The amino acid distributions among different clusters within the same isofunctional group can then be compared to identify same or distinct chemical mechanisms. F1 and F2 indicate two different isofunctional groups within a family. C1, C2, C3, and C4 are conserved isofunctional clusters, among which C1 and C3 were selected in SDP prediction to represent F1 and F2, respectively. (TIF) [file pcbi.1002318.s001.tif]

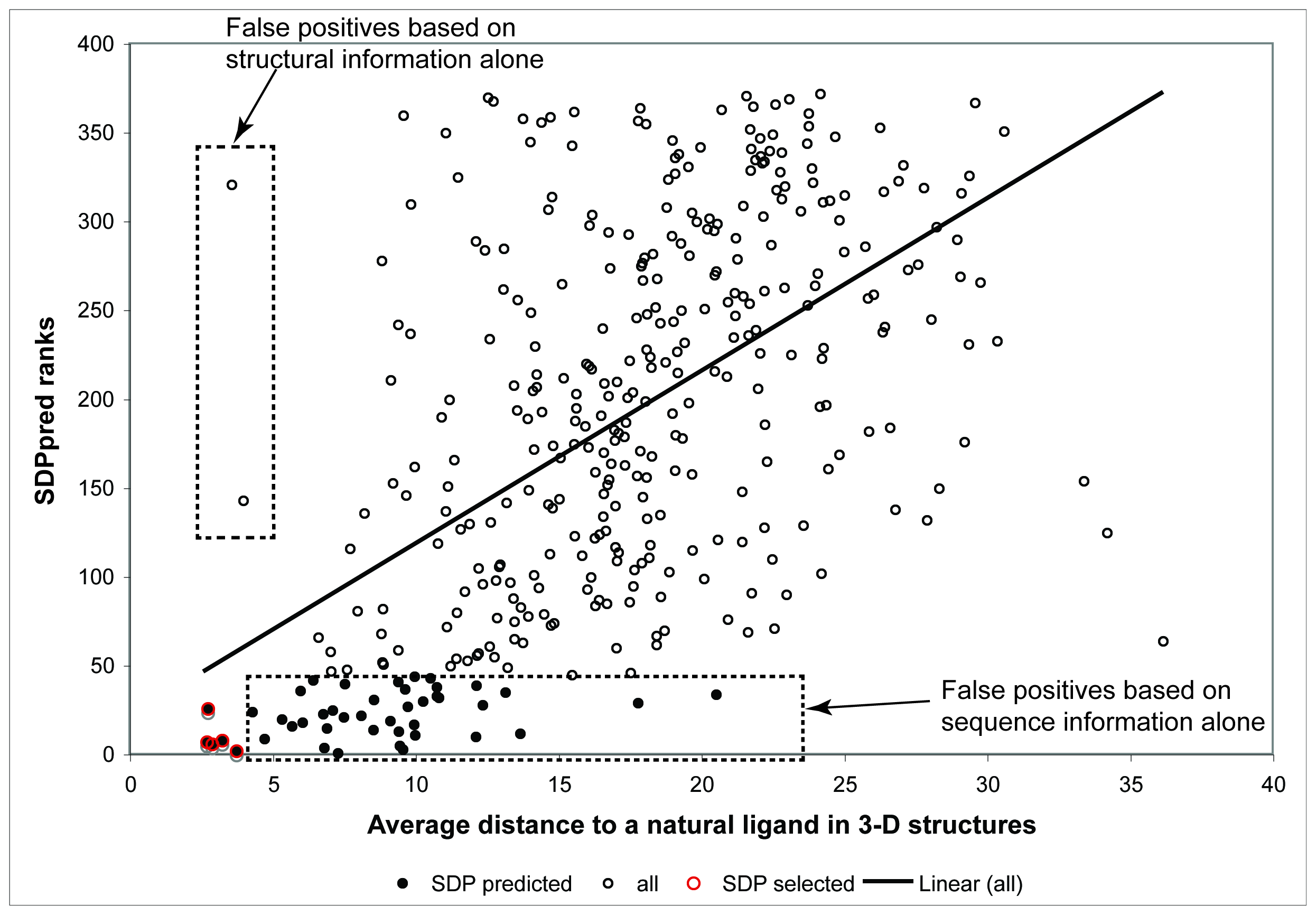

Supplement: Figure S2 — Selection of SDPs in a multiple sequence alignment (MSA) of FGGY kinases combining sequence and structural information. The SDPpred ranking of MSA positions were plotted against their average distances from co-crystallized, functionally relevant ligands in three-dimensional structures. Each data point represents an alignment position. The open circles are all positions in the MSA that have an SDPpred ranking and an average distance value. The filled black dots indicate MSA positions with an SDPpred ranking higher than the global minimum. The red circles indicate the five signature residue positions with and SDPpred ranking higher than the global minimum and the average distance no more than 4 Å. (TIF) [file pcbi.1002318.s002.tif]

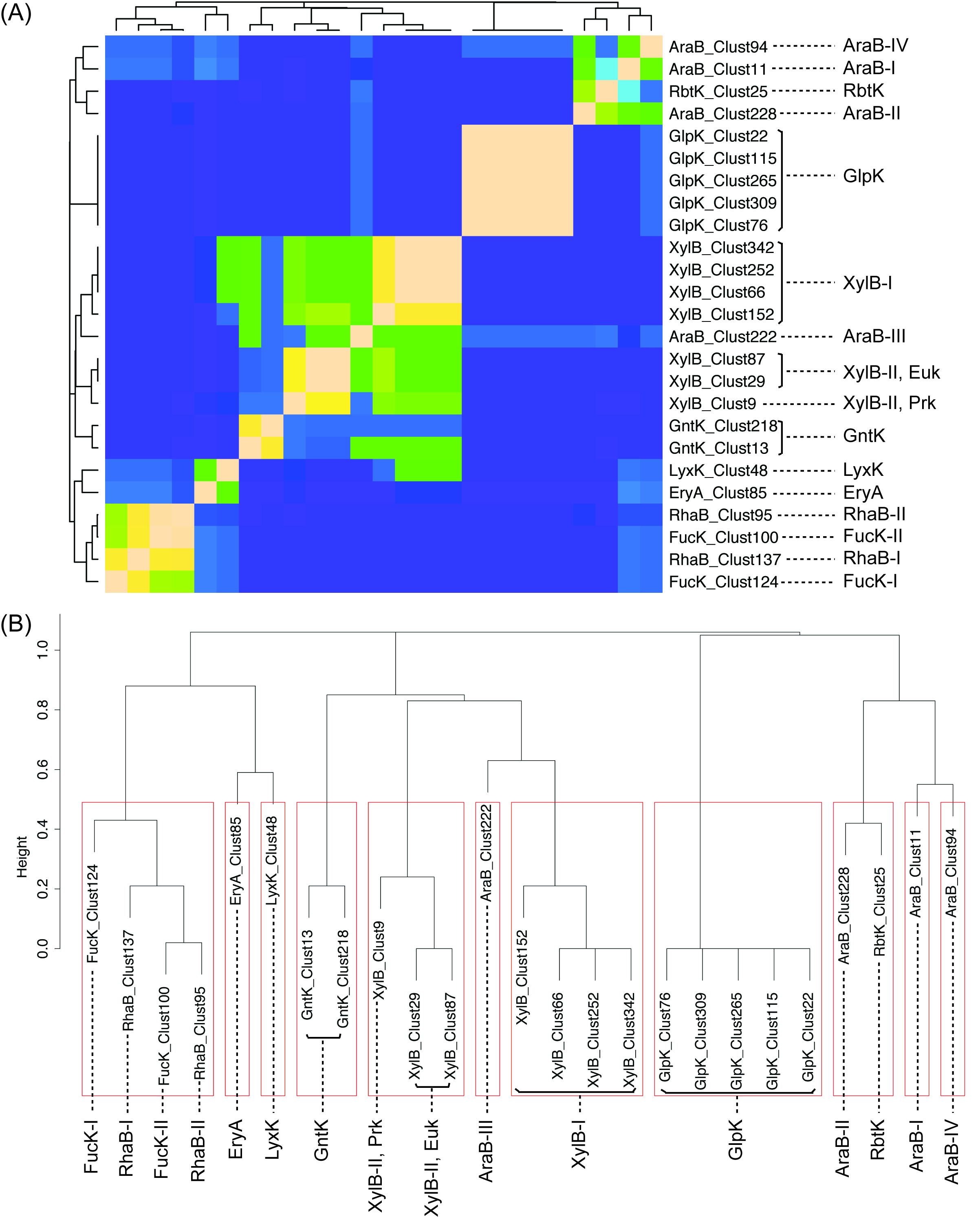

Supplement: Figure S3 — Comparison of SDP signatures among clusters of isofunctional proteins. (A) A heat map based on the correlation coefficients of the signature position weight matrices. The heat map is symmetric, with identical row and column labels. Each cell in the heat map contains the correlation coefficient of two signatures indicated by their column and row labels. The heat map is color coded so that brown indicates higher and blue indicates lower correlation coefficient values. (B) An enlarged version of the similarity tree in the heat map. The tree was built based on a hierarchical clustering approach implemented in the hclust tool in the R software package. Red boxes indicate the global-level clustering (based on signature identity) of the protein clusters. (TIF) [file pcbi.1002318.s003.tif]
