# Supplementary material for: The FGGY Carbohydrate Kinase Family: Insights into the Evolution of Functional Specificities
Source: PLoS Comput Biol. 2011 Dec 22;7(12):e1002318. doi: 10.1371/journal.pcbi.1002318 (PMC3245297; doi:10.1371/journal.pcbi.1002318)
Supplement: Table S1 — List of proteins in the confidently annotated reference set (CARS) with their annotations based on literature and/or context-based analyses. Uniprot_Acc: the Uniprot accession number of proteins; SEED_PEGid: the protein identification number in SEED; Uniprot_Recname: the Uniprot-recommended name of the protein; Uniprot_Subname: the submitter-recommended name of the protein; Organism: organism name from which the protein was identified; Context-based annotation: the annotation based on genomic and functional context analyses; Functional_context: the number of proteins in the genome that perform neighboring functions in a metabolic pathway of the target protein; Genomic_context: the number of proteins in a same operon of the target protein that perform neighboring functions in the metabolic pathway; Reference_annotation: the annotation based on literature; Clust_ID: the cluster numbers of the target proteins in 30% sequence identity clustering. (PDF) [file pcbi.1002318.s004.pdf]

Table S1. **List of proteins in the confidently annotated reference set (CARS)** with their annotations based on literature and/or the context-based analyses. Uniprot_Acc: the Uniprot accession number of proteins; SEED_PEGid: the protein identification number in SEED database; Uniprot_Recname: the Uniprot recommanded name of the protein; Uniprot_Subname: The submitter reommanded name of the protein; Organism: organism name from which the protein was identified; Context-based annotation: the annotation based on genomic and functional context analyses; Functional_context: the number of proteins in the genome that perform neighboring functions in a metabolic pathway of the target protein; Genomic_context: the number of proteins in a same operon of the target protein that perform neighboring functions in the metabolic pathway; Reference_annotation: the annotation based on literature; Clust_ID: the cluster numbers of target protein in 30% sequence identity clustering.

Uniprot_Acc	SEED_PEGid	Uniprot_Recname	Uniprot_Subname	Organism	Context-based Annotation	Functional_context	Genomic_context	Reference annotation	Clust_ID
A3V752	fig|272620.3.peg.3969	-	Xylulokinase	Klebsiella pneumoniae NTUH-K2044.	XylB	3	2	XylB	252
Q0I2L5	fig|83333.1.peg.3498	-	Xylulokinase	Escherichia coli (strain K12 / DH10B).	XylB	3	2	XylB	66
Q2YIQ1	fig|100226.1.peg.1137	Xylulose kinase	-	Streptomyces coelicolor.	XylB	1	1	XylB	9
Q65PY1	fig|387344.13.peg.182	-	Xylulokinase	Lactobacillus brevis (strain ATCC 367 / JCM 1170).	XylB	3	2	XylB	342
Q8NZW9	fig|243274.1.peg.116	-	Sugar kinase, FGGY family	Thermotoga maritima.	XylB	1	0	XylB	152
A0LWM7	fig|350058.5.peg.5216	-	Xylulokinase	Mycobacterium vanbaalenii (strain DSM 7251 / PYR-1).	XylB	2	2	-	9
A1QZ35	fig|349965.3.peg.2781	-	Xylulose kinase	Yersinia intermedia ATCC 29909.	XylB	2	2	-	252
A1TFW7	fig|101510.15.peg.2837	-	Probable xylulokinase	Rhodococcus sp. (strain RHA1).	XylB	2	1	-	9
A2TYE0	fig|221988.1.peg.2230	-	XylB protein	Mannheimia succiniciproducens (strain MBEL55E).	XylB	2	2	-	252
A3I438	fig|220341.1.peg.3657	-	Xylulose kinase	Salmonella typhi.	XylB	1	1	-	252
A3IGA5	fig|349967.3.peg.716	-	Xylulose kinase	Yersinia mollaretii ATCC 43969.	XylB	2	2	-	252
A3SK66	fig|340185.3.peg.30	-	Xylulokinase	Escherichia coli (strain 55989 / EAEC).	XylB	3	2	-	252
A3VKN6	fig|300269.11.peg.4392	-	Xylulokinase	Shigella sonnei (strain Ss046).	XylB	2	1	-	252
A3XK62	fig|273123.1.peg.3950	-	Xylulose kinase	Yersinia pseudotuberculosis.	XylB	2	2	-	252
A3XSZ3	fig|502800.3.peg.55	-	Xylulokinase	Yersinia pseudotuberculosis serotype O:3 (strain YPIII).	XylB	2	2	-	66

A3ZQG2	fig|331112.3.peg.3527	-	Xylulokinase	Escherichia coli 101-1.	XylB	3	2	-	252
A4APS0	fig|349968.3.peg.182	-	Xylulose kinase	Yersinia bercovieri ATCC 43970.	XylB	2	2	-	252
A4BZE8	fig|585057.4.peg.4219	-	Xylulokinase	Escherichia coli O7:K1 (strain IAI39 / ExPEC).	XylB	2	2	-	252
A4CJ47	fig|198215.1.peg.3578	-	Xylulokinase	Shigella flexneri serotype 5b (strain 8401).	XylB	3	2	-	252
A4VJY7	fig|331271.3.peg.1043	-	Xylulokinase	Burkholderia cenocepacia (strain AU 1054).	XylB	3	2	-	252
A5IMF5	fig|187410.1.peg.4002	-	Xylulokinase	Yersinia pestis.	XylB	2	2	-	252
A5MU26	fig|205914.5.peg.613	-	Xylulokinase (Xylulose kinase)	Haemophilus somnus (strain 129Pt) (Histophilus somni (strain 129Pt)).	XylB	2	2	-	252
A5UIF7	fig|481805.3.peg.158	-	Xylulokinase	Escherichia coli (strain ATCC 8739 / DSM 1576 / Crooks).	XylB	3	2	-	66
A6LKN0	fig|300268.10.peg.4268	-	Xylulokinase	Shigella boydii serotype 4 (strain Sb227).	XylB	3	2	-	252
A6V1L3	fig|298386.1.peg.2696	-	Putative xylulose kinase	Photobacterium profundum (Photobacterium sp. (strain SS9)).	XylB	2	1	-	252
A6VPR1	fig|434924.4.peg.559	-	Xylulose kinase	Coxiella burnetii (strain CbuK_Q154) (Coxiella burnetii (strainQ154)).	XylB	1	1	-	252
A8GLA1	fig|342610.3.peg.3714	-	Xylulokinase	Pseudoalteromonas atlantica (strain T6c / BAA-1087).	XylB	3	2	-	252
A8LDF9	fig|281090.3.peg.1380	-	Xylulose kinase	Leifsonia xyli subsp. xyli.	XylB	1	1	-	9
A9WZF0	fig|391037.3.peg.3826	-	Xylulokinase	Salinispora arenicola (strain CNS-205).	XylB	2	1	-	9
B0GU21	fig|399741.3.peg.128	-	Xylulokinase	Serratia proteamaculans (strain 568).	XylB	2	2	-	66
B0UT20	fig|349747.3.peg.2621	-	Xylulokinase	Yersinia pseudotuberculosis serotype O:1b (strain IP 31758).	XylB	2	2	-	66
B0UTW2	fig|584.1.peg.2287	-	Xylulose kinase	Proteus mirabilis (strain HI4320).	XylB	1	1	-	66
B1BSG4	fig|554290.7.peg.3706	-	Xylulose kinase	Salmonella paratyphi A.	XylB	1	1	-	252
B1JH39	fig|400668.6.peg.1903	-	Xylulokinase	Marinomonas sp. (strain MWYL1).	XylB	3	1	-	66
B1LBG8	fig|216593.1.peg.4305	-	Xylulokinase	Escherichia coli O127:H6 (strain E2348/69 / EPEC).	XylB	2	2	-	252
B1RR24	fig|218491.3.peg.3058	-	Xylulose kinase	Erwinia carotovora subsp. atroseptica (Pectobacterium atrosepticum).	XylB	2	2	-	252

B2HNZ2	fig|312284.3.peg.1739	-	Xylulose kinase	marine actinobacterium PHSC20C1.	XylB	1	1	-	9
B3IHD4	fig|227377.1.peg.331	-	Xylulose kinase	Coxiella burnetii.	XylB	2	2	-	252
B5BHV9	fig|83334.1.peg.4418	-	Xylulokinase	Escherichia coli O157:H7 str. EC4501.	XylB	3	2	-	66
B5PRN3	fig|228400.4.peg.987	-	Xylulokinase	Haemophilus somnus (strain 2336) (Histophilus somni (strain 2336)).	XylB	2	2	-	66
B7ULE2	fig|349746.3.peg.3071	-	Xylulokinase	Yersinia pestis biovar Orientalis str. MG05-1020.	XylB	3	3	-	66
B7VAK3	fig|48935.1.peg.1771	-	Xylulokinase	Novosphingobium aromaticivorans (strain DSM 12444).	XylB	2	1	-	252
C2PSC4	fig|272558.1.peg.2756	-	Xylose kinase	Bacillus halodurans.	XylB	1	1	-	342
C4IV00	fig|369723.3.peg.3537	-	Xylulokinase	Salinispora tropica (strain ATCC BAA-916 / DSM 44818 / CNB-440).	XylB	2	1	-	9
C4SXT4	fig|331111.3.peg.1705	-	Xylulokinase	Escherichia coli O139:H28 (strain E24377A / ETEC).	XylB	3	2	-	66
C5C1D9	fig|269800.4.peg.1887	-	Xylulokinase	Thermobifida fusca (strain YX).	XylB	1	1	-	9
Q02R51	fig|349966.3.peg.2473	-	Xylulose kinase	Yersinia frederiksenii ATCC 33641.	XylB	2	2	-	252
Q0SCW9	fig|196627.4.peg.150	-	XYLULOSE KINASE	Corynebacterium glutamicum (Brevibacterium flavum).	XylB	1	1	-	9
Q162I2	fig|298653.4.peg.4564	-	Xylulokinase	Frankia sp. (strain EAN1pec).	XylB	1	1	-	9
Q1AX49	fig|221109.1.peg.3121	-	Xylose kinase (Xylulokinase)	Oceanobacillus iheyensis.	XylB	1	1	-	342
Q1LL59	fig|66692.3.peg.574	-	Xylose kinase	Bacillus clausii (strain KSM-K16).	XylB	1	1	-	342
Q31V54	fig|224308.1.peg.1765	Xylulose kinase	-	Bacillus subtilis.	XylB	1	1	-	342
Q3ICL7	fig|199310.1.peg.4289	-	Xylulose kinase	Escherichia coli O6.	XylB	2	2	-	252

Q3R5A3	fig|279010.5.peg.3168	-	Xylulose kinase	Bacillus licheniformis (strain DSM 13 / ATCC 14580).	XylB	1	1	-	342
Q3YVU9	fig|222523.1.peg.2195	-	Xylulokinase	Bacillus cereus (strain ATCC 10987).	XylB	2	2	-	342
Q4JHR4	fig|471853.4.peg.3727	-	Xylulokinase	Beutenbergia cavernae (strain ATCC BAA-8 / DSM 12333 / NBRC 16432).	XylB	1	1	-	9
Q65WK5	fig|399742.4.peg.239	-	Xylulokinase	Enterobacter sp. (strain 638).	XylB	2	2	-	66
Q92NH0	fig|227882.1.peg.7183	-	Xylulose kinase	Streptomyces avermitilis.	XylB	1	1	-	9
Q93HF4	fig|351607.5.peg.2061	-	Xylulokinase	Acidothermus cellulolyticus (strain ATCC 43068 / 11B).	XylB	2	1	-	9
Q986P3	fig|216594.1.peg.3495	-	Carbohydrate kinase	Mycobacterium marinum (strain ATCC BAA-535 / M).	XylB	1	1	-	9
A4XXN1	fig|83333.1.peg.3825	Rhamnulokinase	-	Escherichia coli (strain K12 / DH10B).	RhaB	2	2	RhaB	137
Q73CE0	fig|99287.1.peg.3901	-	Rhamnulokinase	Salmonella enterica subsp. enterica serovar Saintpaul str. SARA23.	RhaB	2	2	RhaB	137
Q9X0G2	fig|243274.1.peg.1063	-	Sugar kinase	Thermotoga maritima.	RhaB	2	2	RhaB	95
A1VA10	fig|340184.3.peg.1501	-	Rhamnulokinase	Escherichia coli B7A.	RhaB	2	2	-	137
A5EWH1	fig|216593.1.peg.4734	Rhamnulokinase	-	Escherichia coli O127:H6 (strain E2348/69 / EPEC).	RhaB	2	2	-	137
A5INA3	fig|390874.10.peg.1685	-	Carbohydrate kinase, FGGY	Thermotoga petrophila (strain RKU-1 / ATCC BAA-488 / DSM 13995).	RhaB	2	2	-	95
A5VZG7	fig|218491.3.peg.4084	Rhamnulokinase	-	Erwinia carotovora subsp. atroseptica (Pectobacterium atrosepticum).	RhaB	2	2	-	137
A6LCZ1	fig|439842.7.peg.3839	-	Rhamnulokinase	Salmonella enterica subsp. enterica serovar Kentucky str. CDC 191.	RhaB	2	2	-	137
A7X1U3	fig|83334.1.peg.4805	-	Rhamnulokinase	Escherichia coli O157:H7 str. EC869.	RhaB	2	2	-	137

B9DV90	fig|439843.6.peg.4214	Rhamnulokinase	-	Salmonella schwarzengrund (strain CVM19633).	RhaB	2	2	-	137
P94524	fig|585034.4.peg.4001	Rhamnulokinase	-	Escherichia coli (strain 55989 / EAEC).	RhaB	2	2	-	137
Q0SQ01	fig|554290.7.peg.4099	Rhamnulokinase	-	Salmonella paratyphi A (strain AKU_12601).	RhaB	2	2	-	137
Q1IMB2	fig|340186.3.peg.1207	-	Rhamnulokinase	Escherichia coli E110019.	RhaB	2	2	-	137
Q2FYZ5	fig|272994.5.peg.4165	Rhamnulokinase	-	Salmonella paratyphi B (strain ATCC BAA-1250 / SPB7).	RhaB	2	2	-	137
Q2YXR6	fig|550537.3.peg.4090	-	Rhamnulokinase	Salmonella enterica subsp. enterica serovar Saintpaul str. SARA29.	RhaB	2	2	-	137
Q39V17	fig|502800.3.peg.3796	-	Rhamnulokinase	Yersinia pseudotuberculosis serotype O:3 (strain YPIII).	RhaB	2	2	-	137
Q3AB25	fig|362663.8.peg.4143	-	Rhamnulokinase	Escherichia coli F11.	RhaB	2	2	-	137
Q3BYR0	fig|220341.1.peg.3393	Rhamnulokinase	-	Salmonella typhi.	RhaB	2	2	-	137
Q4L607	fig|344601.3.peg.1720	-	Rhamnulokinase	Escherichia coli E22.	RhaB	2	2	-	137
Q4UZR8	fig|331112.3.peg.3858	Rhamnulokinase	-	Escherichia coli O9:H4 (strain HS).	RhaB	2	2	-	137
Q65GC1	fig|454166.6.peg.4164	Rhamnulokinase	-	Salmonella agona (strain SL483).	RhaB	2	2	-	137
Q6G9R3	fig|187410.1.peg.576	Rhamnulokinase	-	Yersinia pestis bv. Antiqua (strain Nepal516).	RhaB	2	2	-	137
Q6GHD5	fig|364106.7.peg.4374	Rhamnulokinase	-	Escherichia coli O45:K1 (strain S88 / ExPEC).	RhaB	2	2	-	137
Q749I1	fig|290338.6.peg.2603	-	Putative uncharacterized protein	Citrobacter koseri (strain ATCC BAA-895 / CDC 4225-83 / SGSC4696).	RhaB	2	2	-	137
Q81GZ2	fig|221988.1.peg.2189	Rhamnulokinase	-	Mannheimia succiniciproducens (strain MBEL55E).	RhaB	2	2	-	137
Q81U58	fig|300268.10.peg.4659	Rhamnulokinase	-	Shigella boydii serotype 4 (strain Sb227).	RhaB	2	2	-	137

Q87BZ2	fig|300269.11.peg.4690	Rhamnulokinase	-	Shigella sonnei (strain Ss046).	RhaB	2	2	-	137
Q8CSS0	fig|198214.1.peg.3748	Rhamnulokinase	-	Shigella flexneri.	RhaB	2	2	-	137
Q8ENK7	fig|585057.4.peg.3200	Rhamnulokinase	-	Escherichia coli O7:K1 (strain IAI39 / ExPEC).	RhaB	2	2	-	137
Q8NWX7	fig|349746.3.peg.2147	Rhamnulokinase	-	Yersinia pestis bv. Antiqua (strain Angola).	RhaB	2	2	-	137
Q8RHZ9	fig|439851.5.peg.4384	Rhamnulokinase	-	Salmonella dublin (strain CT_02021853).	RhaB	2	2	-	137
Q97JG4	fig|454169.6.peg.4312	-	Rhamnulokinase	Salmonella enterica subsp. enterica serovar Heidelberg str. SL486.	RhaB	2	2	-	137
Q9HY41	fig|321314.4.peg.3436	Rhamnulokinase	-	Salmonella paratyphi C (strain RKS4594).	RhaB	2	2	-	137
Q9KDW8	fig|349965.3.peg.3107	-	Rhamnulokinase	Yersinia intermedia ATCC 29909.	RhaB	2	2	-	137
Q9WYC0	fig|266117.6.peg.1540	-	Carbohydrate kinase, FGGY	Rubrobacter xylanophilus (strain DSM 9941 / NBRC 16129).	RhaB	2	1	-	95
Q9X1E4	fig|216598.1.peg.3988	-	Rhamnulokinase	Shigella dysenteriae serotype 1 (strain Sd197).	RhaB	2	2	-	137
A5LQY3	fig|272943.3.peg.3604	-	D-ribulokinase	Rhodobacter sphaeroides (strain ATCC 17023 / 2.4.1 / NCIB 8253 / DSM158).	RbtK	3	1	-	25
A5M8Q4	fig|204722.1.peg.2375	-	Ribitol kinase	Brucella suis.	RbtK	1	1	-	25
A5MG16	fig|216596.1.peg.7085	-	Putative D-ribulokinase/ribitol kinase	Rhizobium leguminosarum bv. viciae (strain 3841).	RbtK	5	1	-	25
B3BWR9	fig|224911.1.peg.3226	-	Ribitol kinase	Bradyrhizobium japonicum.	RbtK	2	1	-	25
B3H0N9	fig|288000.5.peg.1100	-	Putative sugar kinase (Ribulo-/ribitol kinase)	Bradyrhizobium sp. (strain BTAi1 / ATCC BAA-1182).	RbtK	2	1	-	25
B5NTU6	fig|224914.1.peg.3039	-	FGGY-family pentulose kinase	Brucella abortus str. 2308 A.	RbtK	1	1	-	25

Q9K994	fig|344610.3.peg.15	-	Carbohydrate kinase FGGY	Escherichia coli (strain ATCC 8739 / DSM 1576 / Crooks).	LyxK	3	2	LyxK	48
A3GF74	fig|439851.5.peg.4018	-	L-xylulose/3-keto-L-gulonate kinase	Salmonella dublin (strain CT_02021853).	LyxK	3	2	-	48
A7ZTC7	fig|205914.5.peg.801	-	L-xylulose kinase	Haemophilus somnus (strain 129Pt) (Histophilus somni (strain 129Pt)).	LyxK	3	2	-	48
A8ARG8	fig|454169.6.peg.3949	-	L-xylulose/3-keto-L-gulonate kinase	Salmonella enterica subsp. enterica serovar Virchow str. SL491.	LyxK	3	2	-	48
B1IZL3	fig|314290.3.peg.1474	-	Lyx	Vibrio sp. MED222.	LyxK	2	2	-	48
B3H8E2	fig|331111.3.peg.1721	-	Cryptic L-xylulose kinase	Escherichia coli O139:H28 (strain E24377A / ETEC).	LyxK	3	3	-	48
B3HZJ6	fig|99287.1.peg.3550	-	L-xylulose/3-keto-L-gulonate kinase	Salmonella enterica subsp. enterica serovar Saintpaul str. SARA23.	LyxK	3	2	-	48
B3YDJ1	fig|272843.1.peg.1247	Probable L-xylulose kinase	-	Pasteurella multocida.	LyxK	3	2	-	48
B4SWM2	fig|216593.1.peg.4322	-	L-xylulose kinase	Escherichia coli O127:H6 (strain E2348/69 / EPEC).	LyxK	3	2	-	48
B5C9Q2	fig|585057.4.peg.4236	-	L-xylulose kinase	Escherichia coli O7:K1 (strain IAI39 / ExPEC).	LyxK	3	3	-	48
B5EX85	fig|221988.1.peg.46	-	XylB protein	Mannheimia succiniciproducens (strain MBEL55E).	LyxK	3	1	-	48
B5FLF1	fig|373384.10.peg.4447	-	L-xylulose kinase, cryptic	Shigella flexneri serotype 5b (strain 8401).	LyxK	3	2	-	48
B5Q768	fig|349965.3.peg.195	-	L-xylulose/3-keto-L-gulonate kinase	Yersinia intermedia ATCC 29909.	LyxK	3	3	-	48
B5R4R1	fig|374931.9.peg.1736	-	L-xylulose kinase	Haemophilus influenzae (strain PittGG).	LyxK	2	2	-	48
B7L6Z2	fig|439842.7.peg.3463	-	L-xylulose/3-keto-L-gulonate kinase	Salmonella enterica subsp. enterica serovar Kentucky str. CVM29188.	LyxK	3	2	-	48

B7NP80	fig|554290.7.peg.3721	-	Putative L-xylulose kinase	Salmonella paratyphi A (strain AKU_12601).	LyxK	3	2	-	48
C1HSH9	fig|340197.3.peg.583	-	Cryptic L-xylulose kinase	Escherichia coli O6:K15:H31 (strain 536 / UPEC).	LyxK	3	2	-	48
C4VEE9	fig|340184.3.peg.274	-	Cryptic L-xylulose kinase	Escherichia coli B7A.	LyxK	3	3	-	48
Q02YH8	fig|199310.1.peg.4308	-	Cryptic L-xylulose kinase	Escherichia coli O6.	LyxK	3	2	-	48
Q03BB9	fig|364106.7.peg.4035	-	Cryptic L-xylulose kinase	Escherichia coli (strain UTI89 / UPEC).	LyxK	3	2	-	48
Q03TX2	fig|405955.9.peg.3369	-	L-xylulose kinase	Escherichia sp. 3_2_53FAA.	LyxK	3	2	-	48
Q0SY98	fig|71421.1.peg.989	Probable L-xylulose kinase	-	Haemophilus influenzae.	LyxK	3	3	-	48
Q0TBM0	fig|228400.4.peg.1295	-	Carbohydrate kinase FGGY	Haemophilus somnus (strain 2336) (Histophilus somni (strain 2336)).	LyxK	3	2	-	48
Q1R512	fig|454166.6.peg.3780	-	L-xylulose/3-keto-L-gulonate kinase	Salmonella agona (strain SL483).	LyxK	3	2	-	48
Q2GC90	fig|439843.6.peg.3849	-	L-xylulose/3-keto-L-gulonate kinase (L-xylulokinase)	Salmonella enterica subsp. enterica serovar Schwarzengrund str. SL480.	LyxK	3	2	-	48
Q53W24	fig|290338.6.peg.4218	-	Putative uncharacterized protein	Citrobacter koseri (strain ATCC BAA-895 / CDC 4225-83 / SGSC4696).	LyxK	3	3	-	48
Q5WKJ2	fig|340185.3.peg.14	-	Cryptic L-xylulose kinase	Escherichia coli E22.	LyxK	3	3	-	48
Q65DK4	fig|423368.6.peg.3987	-	L-xylulose/3-keto-L-gulonate kinase	Salmonella newport (strain SL254).	LyxK	3	2	-	48
Q739D1	fig|340186.3.peg.1713	-	L-xylulose kinase	Escherichia coli (strain 55989 / EAEC).	LyxK	3	3	-	48
Q8CX70	fig|209261.1.peg.3576	-	Putative L-xylulose kinase	Salmonella typhi.	LyxK	3	2	-	48
Q8FCD0	fig|550537.3.peg.3723	-	Putative L-xylulose kinase	Salmonella enteritidis PT4 (strain P125109).	LyxK	3	2	-	48
Q8Z2C9	fig|272994.5.peg.3802	-	L-xylulose/3-keto-L-gulonate kinase	Salmonella enterica subsp. enterica serovar Hadar str. RI_05P066.	LyxK	3	2	-	48
Q1J5E4	fig|243274.1.peg.437	-	Gluconate kinase	Thermotoga maritima.	GntK	2	2	GntK	218

Q1QVQ3	fig|224308.1.peg.4012	Gluconokinase	-	Bacillus subtilis.	GntK	2	1	GntK	13
A2SM29	fig|198094.1.peg.5316	-	Gluconate kinase, C-terminus	Bacillus anthracis.	GntK	2	1	-	13
A4J8E6	fig|281309.3.peg.3350	-	Gluconate kinase (2-dehydro-3-deoxygluconokinase)	Bacillus thuringiensis (strain Al Hakam).	GntK	2	1	-	13
A4JHM8	fig|315730.5.peg.3652	-	Gluconate kinase	Bacillus weihenstephanensis (strain KBAB4).	GntK	2	1	-	13
A4SPA7	fig|281309.3.peg.161	-	Gluconate kinase	Bacillus thuringiensis subsp. konkukian.	GntK	2	1	-	13
A6M1Y8	fig|220668.1.peg.1044	-	Gluconokinase	Lactobacillus plantarum.	GntK	2	1	-	13
A9MI40	fig|321967.8.peg.241	-	Gluconate kinase	Lactobacillus paracasei subsp. paracasei 8700:2.	GntK	2	1	-	13
B2FI02	fig|272622.8.peg.2621	-	Gluconate kinase	Lactococcus lactis subsp. cremoris (strain SK11).	GntK	2	1	-	13
B4SJT3	fig|261591.3.peg.3922	-	Gluconokinase	Bacillus anthracis (strain CDC 684 / NRRL 3495).	GntK	2	1	-	13
C3KBM0	fig|222523.1.peg.3379	-	Gluconate kinase	Bacillus cereus (strain ATCC 10987).	GntK	2	1	-	13
O51257	fig|278197.10.peg.1196	-	Gluconate kinase	Pediococcus pentosaceus (strain ATCC 25745 / 183-1w).	GntK	2	1	-	13
P57944	fig|543734.3.peg.221	-	Gluconokinase	Lactobacillus casei (strain BL23).	GntK	2	1	-	13
Q0BC36	fig|412694.5.peg.214	-	Gluconokinase	Bacillus cereus (strain 03BB102).	GntK	2	1	-	13
Q0SNS0	fig|279010.5.peg.3745	-	GntK	Bacillus licheniformis (strain DSM 13 / ATCC 14580).	GntK	2	1	-	13
Q13UE3	fig|416870.7.peg.2457	-	Gluconate kinase	Lactococcus lactis subsp. cremoris (strain MG1363).	GntK	2	1	-	13
Q1BTT7	fig|203120.4.peg.541	-	Gluconate kinase	Leuconostoc mesenteroides subsp. mesenteroides (strain ATCC 8293 /NCDO 523).	GntK	2	1	-	13
Q1JAF2	fig|126740.4.peg.495	-	Carbohydrate kinase FGGY	Thermotoga sp. (strain RQ2).	GntK	2	2	-	218
Q3K7I5	fig|269801.1.peg.1152	-	Gluconate kinase	Bacillus cereus G9241.	GntK	3	1	-	13
Q48F01	fig|272623.1.peg.2239	-	Gluconate kinase	Lactococcus lactis subsp. lactis (Streptococcus lactis).	GntK	2	1	-	13

Q49X93	fig|288681.12.peg.158	-	Gluconokinase	Bacillus cereus (strain ZK / E33L).	GntK	2	1	-	13
Q4K734	fig|269801.1.peg.135	-	Gluconate kinase	Bacillus cereus G9241.	GntK	3	1	-	13
Q4ZPI7	fig|101031.3.peg.165	-	Gluconokinase	Bacillus sp. B14905.	GntK	1	1	-	13
Q63X50	fig|340099.4.peg.591	-	Carbohydrate kinase, FGGY	Thermoanaerobacter pseudethanolicus (strain ATCC 33223 / 39E)(Clostridium thermohydrosulfuricum).	GntK	3	1	-	13
Q662C3	fig|226900.1.peg.3220	-	Gluconokinase	Bacillus cereus (strain ATCC 14579 / DSM 31).	GntK	2	1	-	13
Q7P1G2	fig|280355.3.peg.2781	-	Gluconate kinase	Bacillus anthracis str. A0465.	GntK	2	1	-	13
Q83D14	fig|280477.3.peg.4500	-	Gluconate kinase	Bacillus anthracis str. A0193.	GntK	2	1	-	13
Q8Z2Y6	fig|288681.12.peg.3257	-	Gluconate kinase (2-dehydro-3-deoxygluconokinase)	Bacillus cereus (strain ZK / E33L).	GntK	2	1	-	13
A9GMQ4	fig|71421.1.peg.661	-	Glycerol kinase	Haemophilus influenzae PittHH.	GlpK	4	1	GlpK	22
B3PD59	fig|511145.6.peg.4026	-	Glycerol kinase	Escherichia coli O157:H7 str. EC508.	GlpK	6	1	GlpK	22
C4SM10	fig|226185.1.peg.1795	-	Glycerol kinase	Enterococcus faecalis TUSoD Ef11.	GlpK	2	1	GlpK	309
A0KWX5	fig|344609.3.peg.3562	Glycerol kinase	-	Shigella boydii serotype 18 (strain CDC 3083-94 / BS512).	GlpK	5	1	-	22
A0LZ79	fig|312309.3.peg.2894	Glycerol kinase	-	Vibrio fischeri (strain ATCC 700601 / ES114).	GlpK	5	2	-	22
A0RGG3	fig|243365.1.peg.251	Glycerol kinase	-	Chromobacterium violaceum.	GlpK	3	2	-	22
A1AIA2	fig|126740.4.peg.1339	-	Glycerol kinase	Thermotoga sp. (strain RQ2).	GlpK	2	1	-	22

A1JI03	fig|208964.1.peg.3579	Glycerol kinase 2	-	Pseudomonas aeruginosa.	GlpK	4	2	-	22
A1RJD4	fig|434271.3.peg.395	-	Glycerol kinase	Actinobacillus pleuropneumoniae serotype 7 (strain AP76).	GlpK	5	2	-	22
A2RNY7	fig|391008.3.peg.3555	Glycerol kinase	-	Stenotrophomonas maltophilia (strain R551-3).	GlpK	2	1	-	22
A3IAP5	fig|342451.4.peg.903	Glycerol kinase	-	Staphylococcus saprophyticus subsp. saprophyticus (strain ATCC 15305 /DSM 20229).	GlpK	3	2	-	22
A3UPP4	fig|381754.5.peg.1506	-	Glycerol kinase	Pseudomonas aeruginosa (strain PA7).	GlpK	4	2	-	22
A3XLT3	fig|262727.1.peg.1467	-	Glycerol kinase	Haemophilus influenzae 3655.	GlpK	4	1	-	22
A3XSV7	fig|313598.3.peg.1727	-	Glycerol kinase	Polaribacter sp. MED152.	GlpK	3	1	-	22
A4BF97	fig|406561.4.peg.1158	-	Glycerol kinase	Streptococcus pneumoniae SP18-BS74.	GlpK	2	1	-	22
A4CJN0	fig|374931.9.peg.1335	-	Glycerol kinase	Haemophilus influenzae 22.1-21.	GlpK	8	2	-	22
A4Y756	fig|416269.5.peg.361	-	Glycerol kinase	Actinobacillus pleuropneumoniae serotype 5b (strain L20).	GlpK	4	3	-	22
A5EAI3	fig|183190.1.peg.1247	Glycerol kinase	-	Xylella fastidiosa (strain Temecula1 / ATCC 700964).	GlpK	3	2	-	265
A5FKW1	fig|320372.3.peg.3613	-	Glycerol kinase	Burkholderia pseudomallei (strain 1710b).	GlpK	3	2	-	22
A5LLM3	fig|196600.1.peg.2692	Glycerol kinase	-	Vibrio vulnificus (strain YJ016).	GlpK	5	2	-	22
A5LR06	fig|412883.3.peg.366	-	Glycerol kinase	Vibrio cholerae MZO-3.	GlpK	5	3	-	22
A5M1U5	fig|314280.3.peg.5317	-	Glycerol kinase	Photobacterium profundum 3TCK.	GlpK	5	1	-	22
A5MU04	fig|262543.4.peg.1098	-	Glycerol kinase	Exiguobacterium sibiricum (strain DSM 17290 / JCM 13490 / 255-15).	GlpK	5	1	-	22
A6TBJ4	fig|272558.1.peg.1093	Glycerol kinase	-	Bacillus halodurans.	GlpK	3	2	-	265
A6TD84	fig|224326.1.peg.625	Glycerol kinase	-	Borrelia burgdorferi (Lyme disease spirochete).	GlpK	3	2	-	22
A7FHY3	fig|381754.5.peg.1503	-	Glycerol kinase	Pseudomonas aeruginosa (strain PA7).	GlpK	4	2	-	22

A8A707	fig|66692.3.peg.3363	-	Glycerol kinase	Bacillus clausii (strain KSM-K16).	GlpK	4	1	-	115
A8AL29	fig|101031.3.peg.69	-	Glycerol kinase	Bacillus sp. B14905.	GlpK	4	1	-	22
A8AP16	fig|227377.1.peg.889	Glycerol kinase	-	Coxiella burnetii.	GlpK	2	1	-	22
A8FBF0	fig|418136.4.peg.209	Glycerol kinase	-	Francisella tularensis subsp. tularensis (strain WY96-3418).	GlpK	3	2	-	22
A9MZC6	fig|340099.4.peg.528	Glycerol kinase	-	Thermoanaerobacter pseudethanolicus (strain ATCC 33223 / 39E)(Clostridium thermohydrosulfuricum).	GlpK	3	2	-	115
A9N2J3	fig|349161.4.peg.2797	Glycerol kinase	-	Desulfotomaculum reducens (strain MI-1).	GlpK	4	3	-	22
A9QYS1	fig|279010.5.peg.1669	Glycerol kinase	-	Bacillus licheniformis (strain DSM 13 / ATCC 14580).	GlpK	3	2	-	115
A9VMK7	fig|195102.1.peg.2615	-	Glycerol kinase	Clostridium perfringens E str. JGS1987.	GlpK	5	1	-	22
B0GHX8	fig|314288.3.peg.430	-	Glycerol kinase	Vibrio alginolyticus 12G01.	GlpK	3	2	-	22
B0K7L5	fig|40324.1.peg.3273	Glycerol kinase	-	Stenotrophomonas maltophilia (strain K279a).	GlpK	2	1	-	22
B0PX16	fig|269482.4.peg.5988	Glycerol kinase	-	Burkholderia vietnamiensis (strain G4 / LMG 22486) (Burkholderiacepacia (strain R1808)).	GlpK	3	2	-	22
B1GKI9	fig|205922.3.peg.5515	Glycerol kinase	-	Pseudomonas fluorescens (strain Pf0-1).	GlpK	3	2	-	22
B1I9X7	fig|349521.5.peg.6106	-	Glycerol kinase	Hahella chejuensis (strain KCTC 2396).	GlpK	4	1	-	22
B1JQ85	fig|314232.3.peg.2627	-	Glycerol kinase	Loktanella vestfoldensis SKA53.	GlpK	2	1	-	22
B1L950	fig|190304.1.peg.318	Glycerol kinase	-	Fusobacterium nucleatum subsp. nucleatum.	GlpK	2	1	-	22
B1S255	fig|314290.3.peg.1506	-	Glycerol kinase	Vibrio sp. MED222.	GlpK	3	2	-	22
B1S281	fig|406558.4.peg.25	-	Glycerol kinase	Streptococcus pneumoniae SP9-BS68.	GlpK	2	1	-	22
B1YE68	fig|391009.4.peg.651	-	Glycerol kinase	Thermosipho melanesiensis (strain BI429 / DSM 12029).	GlpK	2	1	-	22
B1YKL3	fig|439842.7.peg.3882	-	Glycerol kinase	Salmonella enterica subsp. enterica serovar Kentucky str. CDC 191.	GlpK	5	1	-	22

B2DHW6	fig|290338.6.peg.2577	Glycerol kinase	-	Citrobacter koseri (strain ATCC BAA-895 / CDC 4225-83 / SGSC4696).	GlpK	6	1	-	22
B2DUN0	fig|208963.3.peg.1118	-	Glycerol kinase	Pseudomonas aeruginosa (strain LESB58).	GlpK	4	2	-	22
B2GE12	fig|246194.3.peg.2378	Glycerol kinase	-	Carboxydothermus hydrogenoformans (strain Z-2901 / DSM 6008).	GlpK	4	3	-	265
B2K4K8	fig|416870.7.peg.1098	-	GlpK protein	Lactococcus lactis subsp. cremoris (strain MG1363).	GlpK	7	2	-	22
B2UN37	fig|379731.4.peg.1612	-	Glycerol kinase	Pseudomonas stutzeri (strain A1501).	GlpK	4	2	-	22
B3BFS9	fig|418127.4.peg.1195	Glycerol kinase	-	Staphylococcus aureus (strain Mu3 / ATCC 700698).	GlpK	3	2	-	22
B3DTJ7	fig|222523.1.peg.1120	Glycerol kinase	-	Bacillus cereus (strain ATCC 10987).	GlpK	3	2	-	265
B3HBT3	fig|204669.6.peg.2985	Glycerol kinase	-	Acidobacteria bacterium (strain Ellin345).	GlpK	4	1	-	22
B3HPH9	fig|441952.3.peg.234	Glycerol kinase	-	Francisella tularensis subsp. mediasiatica (strain FSC147).	GlpK	3	2	-	22
B3HTS7	fig|272562.1.peg.1479	Glycerol kinase	-	Clostridium acetobutylicum.	GlpK	5	2	-	22
B3HY55	fig|316275.9.peg.3623	Glycerol kinase	-	Aliivibrio salmonicida (strain LFI1238) (Vibrio salmonicida (strainLFI1238)).	GlpK	5	2	-	22
B3I5Y6	fig|314724.3.peg.234	-	Glycerol kinase	Borrelia turicatae (strain 91E135).	GlpK	3	2	-	22
B3IHB1	fig|282458.1.peg.1205	Glycerol kinase	-	Staphylococcus aureus (strain MRSA252).	GlpK	3	2	-	22
B3IQ43	fig|269483.3.peg.7487	-	Glycerol kinase	Burkholderia sp. (strain 383) (Burkholderia cepacia (strain ATCC 17760/ NCIB 9086 / R18194)).	GlpK	4	2	-	22
B3IQX1	fig|557723.7.peg.1050	-	Glycerol kinase	Haemophilus parasuis serovar 5 (strain SH0165).	GlpK	5	1	-	22
B3W8Q2	fig|216595.1.peg.7475	Glycerol kinase	-	Pseudomonas fluorescens (strain SBW25).	GlpK	3	2	-	22
B3WR88	fig|388396.7.peg.3479	Glycerol kinase	-	Vibrio fischeri (strain MJ11).	GlpK	5	2	-	22
B3X839	fig|370553.3.peg.1404	Glycerol kinase	-	Streptococcus pyogenes serotype M12 (strain MGAS2096).	GlpK	2	1	-	309

B3XIM5	fig|343509.6.peg.4788	-	Glycerol kinase	Sodalis glossinidius (strain morsitans).	GlpK	5	2	-	22
B3YJZ6	fig|41514.4.peg.3400	Glycerol kinase	-	Salmonella arizonae (strain ATCC BAA-731 / CDC346-86 / RSK2980).	GlpK	5	1	-	22
B4BKR7	fig|585034.4.peg.4025	Glycerol kinase	-	Escherichia coli O8 (strain IAI1).	GlpK	6	1	-	22
B4F166	fig|390874.10.peg.1375	-	Glycerol kinase	Thermotoga petrophila (strain RKU-1 / ATCC BAA-488 / DSM 13995).	GlpK	2	1	-	22
B4T4X2	fig|220341.1.peg.3356	Glycerol kinase	-	Salmonella typhi.	GlpK	5	1	-	22
B4TGN4	fig|339671.5.peg.1576	-	Glycerol kinase	Actinobacillus succinogenes (strain ATCC 55618 / 130Z).	GlpK	4	1	-	22
B4TPQ8	fig|487214.3.peg.2311	Glycerol kinase	-	Streptococcus pneumoniae (strain Hungary19A-6).	GlpK	2	1	-	115
B5BJG7	fig|66692.3.peg.924	-	Glycerol kinase	Bacillus clausii (strain KSM-K16).	GlpK	4	1	-	115
B5C7K8	fig|273036.3.peg.1482	Glycerol kinase	-	Staphylococcus aureus (strain bovine RF122 / ET3-1).	GlpK	3	2	-	22
B5CBW6	fig|406563.4.peg.807	-	Glycerol kinase	Streptococcus pneumoniae SP23-BS72.	GlpK	2	1	-	22
B5F0M8	fig|453363.3.peg.1184	-	Glycerol kinase	Streptococcus pneumoniae CDC1873-00.	GlpK	2	1	-	115
B5F4S4	fig|264730.3.peg.4170	Glycerol kinase	-	Pseudomonas syringae pv. phaseolicola (strain 1448A / Race 6).	GlpK	3	2	-	22
B5FTY2	fig|272843.1.peg.1447	Glycerol kinase	-	Pasteurella multocida.	GlpK	4	3	-	22
B5MMZ8	fig|314230.3.peg.1516	-	Glycerol kinase	Blastopirellula marina DSM 3645.	GlpK	2	1	-	22
B5MV63	fig|314292.13.peg.2707	-	Glycerol kinase	Photobacterium angustum (strain S14 / CCUG 15956) (Vibrio sp. (strainS14 / CCUG 15956)).	GlpK	3	2	-	22
B5NMB4	fig|208964.1.peg.3582	Glycerol kinase 1	-	Pseudomonas aeruginosa.	GlpK	4	2	-	22
B5NTQ5	fig|279808.3.peg.2592	Glycerol kinase	-	Staphylococcus haemolyticus (strain JCSC1435).	GlpK	3	2	-	22
B5P7I0	fig|313594.3.peg.1540	-	Glycerol kinase	Polaribacter irgensii 23-P.	GlpK	3	2	-	22

B6J5X8	fig|198094.1.peg.955	Glycerol kinase	-	Bacillus anthracis.	GlpK	3	2	-	265
B7L9G0	fig|406557.4.peg.2267	-	Glycerol kinase	Streptococcus pneumoniae CDC1087-00.	GlpK	3	1	-	115
B7MLC4	fig|406560.4.peg.1337	-	Glycerol kinase	Streptococcus pneumoniae SP14-BS69.	GlpK	2	1	-	22
B7NP64	fig|370554.3.peg.1492	Glycerol kinase	-	Streptococcus pyogenes serotype M4 (strain MGAS10750).	GlpK	3	1	-	309
B7NUA2	fig|484022.4.peg.1045	Glycerol kinase	-	Francisella philomiragia subsp. philomiragia (strain ATCC 25017).	GlpK	3	2	-	115
B7UHL9	fig|340186.3.peg.1230	-	Glycerol kinase	Escherichia coli E110019.	GlpK	5	1	-	22
B7ULC5	fig|220668.1.peg.311	Glycerol kinase 1	-	Lactobacillus plantarum.	GlpK	5	2	-	309
C0Q3L3	fig|467705.8.peg.609	Glycerol kinase	-	Streptococcus gordonii (strain Challis / ATCC 35105 / CH1 / DL1 /V288).	GlpK	2	1	-	115
C1EU16	fig|313596.3.peg.1776	-	Glycerol kinase	Robiginitalea biformata (strain ATCC BAA-864 / HTCC2501 / KCTC 12146).	GlpK	3	1	-	22
C3LJZ6	fig|435591.10.peg.1729	Glycerol kinase	-	Parabacteroides distasonis (strain ATCC 8503 / DSM 20701 / NCTC11152).	GlpK	3	2	-	22
C3SWV3	fig|99287.1.peg.3940	Glycerol kinase	-	Salmonella typhimurium.	GlpK	5	1	-	22
C4B4W2	fig|266264.4.peg.2636	-	Glycerol kinase	Ralstonia metallidurans (strain CH34 / ATCC 43123 / DSM 2839).	GlpK	3	1	-	265
C4IWD3	fig|196620.1.peg.1183	Glycerol kinase	-	Staphylococcus aureus (strain MW2).	GlpK	3	2	-	265
C4S052	fig|286636.1.peg.1429	Glycerol kinase	-	Streptococcus pyogenes serotype M6.	GlpK	3	1	-	309
C4S4W9	fig|345072.3.peg.1663	Glycerol kinase	-	Vibrio cholerae.	GlpK	5	3	-	22
C4S6A1	fig|326442.4.peg.3476	-	Putative glycerol kinase	Pseudoalteromonas haloplanktis (strain TAC 125).	GlpK	3	2	-	22
C4SAH6	fig|338187.4.peg.2422	Glycerol kinase	-	Vibrio harveyi (strain ATCC BAA-1116 / BB120).	GlpK	3	2	-	22

C4SC56	fig|198466.1.peg.1468	Glycerol kinase	-	Streptococcus pyogenes serotype M3.	GlpK	2	1	-	309
C4SS63	fig|399739.6.peg.3233	Glycerol kinase	-	Pseudomonas mendocina (strain ymp).	GlpK	3	1	-	22
C4STW2	fig|223926.1.peg.2386	Glycerol kinase	-	Vibrio parahaemolyticus.	GlpK	3	2	-	22
C4T286	fig|186103.1.peg.1395	Glycerol kinase	-	Streptococcus pyogenes serotype M18.	GlpK	3	1	-	309
C4T5B8	fig|208963.3.peg.1115	-	Putative glycerol kinase	Pseudomonas aeruginosa (strain UCBPP-PA14).	GlpK	4	2	-	22
C4T6J3	fig|101031.3.peg.804	-	Glycerol kinase	Bacillus sp. B14905.	GlpK	4	2	-	22
C4X272	fig|278197.10.peg.1429	Glycerol kinase	-	Pediococcus pentosaceus (strain ATCC 25745 / 183-1w).	GlpK	4	1	-	309
C5F6I2	fig|205918.4.peg.4340	Glycerol kinase	-	Pseudomonas syringae pv. syringae (strain B728a).	GlpK	3	2	-	22
P21939	fig|349968.3.peg.2516	-	Glycerol kinase	Yersinia bercovieri ATCC 43970.	GlpK	5	1	-	22
P39211	fig|349124.5.peg.78	Glycerol kinase	-	Halorhodospira halophila (strain DSM 244 / SL1) (Ectothiorhodospira halophila (strain DSM 244 / SL1)).	GlpK	2	1	-	22
P44991	fig|630.2.peg.98	-	Glycerol kinase	Yersinia enterocolitica serotype O:8 / biotype 1B (strain 8081).	GlpK	5	1	-	22
P57928	fig|349965.3.peg.2953	-	Glycerol kinase	Yersinia intermedia ATCC 29909.	GlpK	5	1	-	22
Q02VU6	fig|351746.4.peg.1100	Glycerol kinase	-	Pseudomonas putida (strain F1 / ATCC 700007).	GlpK	3	2	-	22
Q03DS8	fig|298386.1.peg.2471	-	Putative ATP:glycerol 3-phosphotransferase (Glycerokinase)(GK)	Photobacterium profundum (Photobacterium sp. (strain SS9)).	GlpK	5	1	-	22
Q03EH2	fig|290398.4.peg.2121	Glycerol kinase	-	Chromohalobacter salexigens (strain DSM 3043 / ATCC BAA-138 / NCIMB13768).	GlpK	1	1	-	22
Q03HQ2	fig|314565.3.peg.388	Glycerol kinase	-	Xanthomonas campestris pv. campestris (strain 8004).	GlpK	3	2	-	265
Q03PR3	fig|289380.14.peg.2479	Glycerol kinase	-	Clostridium perfringens (strain SM101 / Type A).	GlpK	5	2	-	265
Q03XW0	fig|266117.6.peg.975	-	Glycerol kinase	Rubrobacter xylanophilus (strain DSM 9941 / NBRC 16129).	GlpK	2	1	-	265

Q03YQ6	fig|89187.3.peg.1225	-	Glycerol kinase	Roseovarius nubinhibens ISM.	GlpK	2	1	-	22
Q04H58	fig|316273.3.peg.591	Glycerol kinase	-	Xanthomonas campestris pv. vesicatoria (strain 85-10).	GlpK	3	2	-	265
Q04I07	fig|357804.5.peg.2985	Glycerol kinase	-	Psychromonas ingrahamii (strain 37).	GlpK	3	1	-	22
Q0HIR7	fig|550537.3.peg.4131	Glycerol kinase	-	Salmonella paratyphi A (strain AKU_12601).	GlpK	5	1	-	22
Q0HV69	fig|374930.8.peg.1737	Glycerol kinase	-	Haemophilus influenzae (strain PittEE).	GlpK	7	3	-	22
Q15PG1	fig|315730.5.peg.1465	-	Glycerol kinase	Bacillus cereus AH621.	GlpK	3	2	-	265
Q1BG91	fig|243231.1.peg.2744	Glycerol kinase	-	Geobacter sulfurreducens.	GlpK	2	1	-	265
Q1CEB3	fig|235909.3.peg.209	Glycerol kinase	-	Geobacillus kaustophilus.	GlpK	3	1	-	115
Q1M8S5	fig|367830.3.peg.820	Glycerol kinase	-	Staphylococcus aureus (strain NCTC 8325).	GlpK	3	2	-	265
Q1YZX9	fig|557722.3.peg.1500	-	Probable carbohydrate kinase	Pseudomonas aeruginosa (strain LESB58).	GlpK	4	2	-	22
Q21MP5	fig|585057.4.peg.3176	Glycerol kinase	-	Escherichia coli O7:K1 (strain IAI39 / ExPEC).	GlpK	6	1	-	22
Q2S6Z4	fig|314271.3.peg.3568	-	Glycerol kinase	Rhodobacterales bacterium HTCC2654.	GlpK	3	1	-	22
Q32A69	fig|391774.5.peg.375	Glycerol kinase	-	Desulfovibrio vulgaris subsp. vulgaris (strain DP4).	GlpK	5	2	-	22
Q32CB5	fig|399741.3.peg.4775	-	Glycerol kinase	Serratia proteamaculans (strain 568).	GlpK	5	1	-	22
Q3IVW9	fig|282459.1.peg.1227	Glycerol kinase	-	Staphylococcus aureus (strain MSSA476).	GlpK	3	2	-	265
Q3YV72	fig|420246.5.peg.1187	-	Glycerol kinase	Geobacillus sp. G11MC16.	GlpK	3	1	-	115
Q3YY54	fig|388919.8.peg.1620	Glycerol kinase	-	Streptococcus sanguinis (strain SK36).	GlpK	3	1	-	22
Q48RX6	fig|405955.9.peg.3694	-	Glycerol kinase	Escherichia coli (strain UTI89 / UPEC).	GlpK	5	1	-	22

Q4MUX7	fig|36873.1.peg.5233	Glycerol kinase	-	Burkholderia xenovorans (strain LB400).	GlpK	3	2	-	22
Q4MXU4	fig|290434.1.peg.339	Glycerol kinase	-	Borrelia garinii.	GlpK	3	2	-	22
Q5PEK8	fig|220664.3.peg.306	Glycerol kinase	-	Pseudomonas fluorescens (strain Pf-5 / ATCC BAA-477).	GlpK	4	2	-	22
Q5PLM7	fig|48935.1.peg.3229	-	Glycerol kinase	Novosphingobium aromaticivorans (strain DSM 12444).	GlpK	3	1	-	265
Q5WCJ3	fig|393115.8.peg.138	Glycerol kinase	-	Francisella tularensis subsp. tularensis.	GlpK	3	2	-	22
Q5WIL1	fig|399742.4.peg.3849	Glycerol kinase	-	Enterobacter sp. (strain 638).	GlpK	5	1	-	22
Q5XAJ9	fig|155864.1.peg.4863	-	Glycerol kinase	Escherichia coli O1:K1 / APEC.	GlpK	5	1	-	22
Q638K2	fig|331271.3.peg.6140	Glycerol kinase	-	Burkholderia cenocepacia (strain AU 1054).	GlpK	2	1	-	22
Q63H40	fig|320390.3.peg.2705	Glycerol kinase	-	Burkholderia pseudomallei (Pseudomonas pseudomallei).	GlpK	3	2	-	22
Q65CW4	fig|339670.3.peg.434	Glycerol kinase	-	Burkholderia ambifaria (strain ATCC BAA-244 / AMMD) (Burkholderiacepacia (strain AMMD)).	GlpK	3	2	-	22
Q65Q24	fig|262543.4.peg.718	-	Glycerol kinase	Exiguobacterium sibiricum (strain DSM 17290 / JCM 13490 / 255-15).	GlpK	5	2	-	115
Q663Y2	fig|321967.8.peg.655	-	Glycerol kinase	Lactobacillus casei (strain ATCC 334).	GlpK	4	2	-	309
Q6DB06	fig|300852.3.peg.2129	-	Glycerol kinase	Thermus thermophilus (strain HB8 / ATCC 27634 / DSM 579).	GlpK	2	1	-	265
Q6HPK8	fig|290402.34.peg.4428	Glycerol kinase	-	Clostridium beijerinckii (strain ATCC 51743 / NCIMB 8052) (Clostridiumacetobutylicum).	GlpK	5	2	-	22
Q6I4N1	fig|176279.3.peg.254	Glycerol kinase	-	Staphylococcus epidermidis (strain ATCC 12228).	GlpK	3	2	-	22
Q6LUX7	fig|272623.1.peg.1281	Glycerol kinase	-	Lactococcus lactis subsp. lactis (Streptococcus lactis).	GlpK	6	2	-	309
Q6LVI8	fig|313603.3.peg.2550	-	Glycerol kinase	Flavobacteriales bacterium HTCC2170.	GlpK	3	2	-	22
Q734J8	fig|382245.6.peg.2531	Glycerol kinase	-	Aeromonas salmonicida (strain A449).	GlpK	5	2	-	22

Q81B23	fig|195103.9.peg.2755	-	Glycerol kinase	Clostridium perfringens NCTC 8239.	GlpK	6	2	-	22
Q83EH5	fig|269799.3.peg.1993	Glycerol kinase	-	Geobacter metallireducens (strain GS-15 / ATCC 53774 / DSM 7210).	GlpK	2	1	-	265
Q88S82	fig|221109.1.peg.2474	Glycerol kinase	-	Oceanobacillus iheyensis.	GlpK	3	2	-	265
Q88XF6	fig|420662.8.peg.3445	Glycerol kinase	-	Methylibium petroleiphilum (strain PM1).	GlpK	2	1	-	22
Q88ZF1	fig|584.1.peg.1407	-	Glycerol kinase	Proteus mirabilis (strain HI4320).	GlpK	5	1	-	22
Q89QA4	fig|155920.1.peg.1144	-	Glycerol kinase	Xylella fastidiosa subsp. sandyi Ann-1.	GlpK	3	2	-	265
Q8CZH7	fig|272622.8.peg.1629	-	Glycerol kinase	Lactococcus lactis subsp. cremoris (strain SK11).	GlpK	6	2	-	309
Q8E794	fig|349746.3.peg.2939	-	Glycerol kinase	Yersinia pseudotuberculosis serotype O:3 (strain YPIII).	GlpK	6	1	-	22
Q8FCE4	fig|208435.1.peg.272	Glycerol kinase	-	Streptococcus agalactiae serotype III.	GlpK	3	1	-	309
Q8FX21	fig|405532.4.peg.931	Glycerol kinase	-	Bacillus cereus (strain ATCC 14579 / DSM 31).	GlpK	3	2	-	265
Q8XNL6	fig|150340.3.peg.367	-	Glycerol kinase	Vibrio sp. (strain Ex25).	GlpK	3	2	-	22
Q8Z2B7	fig|319701.3.peg.1805	Glycerol kinase	-	Streptococcus pyogenes serotype M28.	GlpK	2	1	-	309
Q8Z2V4	fig|315750.5.peg.795	-	Glycerol kinase	Bacillus pumilus (strain SAFR-032).	GlpK	3	2	-	115
Q97JE2	fig|218495.3.peg.1315	Glycerol kinase	-	Streptococcus uberis (strain ATCC BAA-854 / 0140J).	GlpK	3	1	-	265
Q97N88	fig|88888881.3.peg.2620	-	Glycerol kinase	Vibrio cholerae 1587.	GlpK	5	3	-	22
Q9CDN5	fig|390236.5.peg.302	Glycerol kinase	-	Borrelia afzelii (strain PKo).	GlpK	3	2	-	22
Q9CG64	fig|349966.3.peg.3323	-	Glycerol kinase	Yersinia frederiksenii ATCC 33641.	GlpK	5	1	-	22
Q9RK00	fig|314291.3.peg.4041	-	Glycerol kinase	Vibrio splendidus 12B01.	GlpK	3	2	-	22
Q9WXX1	fig|313593.3.peg.1786	-	Glycerol kinase	Leeuwenhoekiella blandensis MED217.	GlpK	3	2	-	22
Q9WYS4	fig|246195.3.peg.210	Glycerol kinase	-	Dichelobacter nodosus (strain VCS1703A).	GlpK	3	2	-	22

B6ER09	fig|83333.1.peg.2759	L-fuculokinase	-	Escherichia coli (strain K12).	FucK	3	3	FucK	124
C1Z4Y4	fig|171101.1.peg.2172	-	L-fuculose kinase FucK, putative	Streptococcus pneumoniae serotype 2 (strain D39 / NCTC 7466).	FucK	3	2	FucK	100
A3CPV3	fig|272994.5.peg.3090	-	Putative uncharacterized protein	Salmonella paratyphi B (strain ATCC BAA-1250 / SPB7).	FucK	3	3	-	124
A4IW85	fig|454166.6.peg.3047	-	L-fuculokinase	Salmonella agona (strain SL483).	FucK	3	3	-	124
A4WG72	fig|272620.3.peg.3157	-	L-fuculokinase	Klebsiella pneumoniae subsp. pneumoniae (strain ATCC 700721 / MGH78578).	FucK	3	3	-	124
A5UE44	fig|349741.3.peg.1944	-	L-fuculokinase	Akkermansia muciniphila (strain ATCC BAA-835).	FucK	2	1	-	124
A7N1R1	fig|155864.1.peg.3666	-	L-fuculokinase	Escherichia coli.	FucK	3	3	-	124
A8AL00	fig|439843.6.peg.3147	-	L-fuculokinase	Salmonella enterica subsp. enterica serovar Schwarzengrund str. SL480.	FucK	3	3	-	124
B2SF32	fig|290338.6.peg.3495	-	Putative uncharacterized protein	Citrobacter koseri (strain ATCC BAA-895 / CDC 4225-83 / SGSC4696).	FucK	3	3	-	124
B2TWC2	fig|439842.7.peg.2718	-	L-fuculokinase	Salmonella enterica subsp. enterica serovar Kentucky str. CVM29188.	FucK	3	3	-	124
B5BJK3	fig|99287.1.peg.2872	-	L-fuculokinase	Salmonella heidelberg (strain SL476).	FucK	3	3	-	124
B5ET10	fig|216593.1.peg.1270	-	L-fuculokinase	Escherichia coli O127:H6 (strain E2348/69 / EPEC).	FucK	3	3	-	124
B7M6X7	fig|220341.1.peg.2762	L-fuculokinase	-	Salmonella typhi.	FucK	3	3	-	124
B7NU81	fig|550538.3.peg.3066	-	L-fuculokinase	Salmonella dublin (strain CT_02021853).	FucK	3	3	-	124
C1Q0P5	fig|170187.1.peg.2028	-	Putative L-fuculose kinase fucK	Streptococcus pneumoniae.	FucK	3	2	-	100

C1RTJ4	fig|406559.4.peg.1306	-	D-alanine--poly(Phosphoribitol) ligase subunit 2	Streptococcus pneumoniae SP11-BS70.	FucK	3	2	-	100
C1U043	fig|453362.3.peg.1376	-	Rhamnulokinase (Rhamnulose kinase)	Streptococcus pneumoniae CDC1873-00.	FucK	3	2	-	100
C1W5M4	fig|453364.8.peg.1218	-	Rhamnulokinase (Rhamnulose kinase)	Streptococcus pneumoniae CDC0288-04.	FucK	3	2	-	100
C1Z7E3	fig|406563.4.peg.788	-	L-fuculose kinase fucK, putative	Streptococcus pneumoniae SP23-BS72.	FucK	3	2	-	100
C4BFM5	fig|406557.4.peg.932	-	L-fuculose kinase FucK, putative	Streptococcus pneumoniae SP6-BS73.	FucK	3	2	-	100
C4CLB0	fig|453365.3.peg.1417	-	Rhamnulokinase (Rhamnulose kinase)	Streptococcus pneumoniae (strain Hungary19A-6).	FucK	3	2	-	100
C4EMX7	fig|1313.3.peg.1747	-	D-alanine--poly(Phosphoribitol) ligase subunit 2	Streptococcus pneumoniae SP9-BS68.	FucK	4	1	-	100
C4EWK3	fig|195102.1.peg.380	-	Rhamnulokinase	Clostridium perfringens.	FucK	2	2	-	100
Q51390	fig|405955.9.peg.2580	-	L-fuculokinase	Escherichia coli O45:K1 (strain S88 / ExPEC).	FucK	3	3	-	124
Q5E0Z0	fig|216598.1.peg.49	-	L-fuculokinase	Shigella dysenteriae serotype 1 (strain Sd197).	FucK	3	2	-	124
Q5NIE5	fig|554290.7.peg.2999	-	L-fuculose kinase	Salmonella paratyphi A.	FucK	3	3	-	124
Q7MI93	fig|340186.3.peg.4322	-	L-fuculokinase	Escherichia coli E110019.	FucK	3	3	-	124
Q87M72	fig|340197.3.peg.1264	-	L-fuculokinase	Escherichia coli F11.	FucK	3	3	-	124
Q8ZKP3	fig|423368.6.peg.3277	-	L-fuculokinase	Salmonella newport (strain SL254).	FucK	3	3	-	124
Q9KLJ9	fig|216599.1.peg.1785	-	L-fuculokinase	Shigella sonnei (strain Ss046).	FucK	3	3	-	124
A7FP69	fig|262698.3.peg.2303	-	EryA, erythritol kinase	Brucella abortus str. 2308 A.	EryA	2	2	EryA	85
A4W567	fig|288000.5.peg.3260	-	Putative carbohydrate kinase	Bradyrhizobium sp. (strain BTAi1 / ATCC BAA-1182).	EryA	2	2	-	85

A5EGP3	fig|470137.3.peg.826	-	Putative uncharacterized protein	Brucella suis (strain ATCC 23445 / NCTC 10510).	EryA	2	2	-	85
A6VWG3	fig|430066.3.peg.382	-	Carbohydrate kinase, FGGY	Brucella abortus (strain 2308).	EryA	1	1	-	85
A8G7W7	fig|224914.1.peg.2490	-	Erythritol kinase	Brucella melitensis.	EryA	2	2	-	85
B1IZM8	fig|114615.3.peg.4624	-	Putative carbohydrate kinase (Xylulose/erythritol kinase, lyx/eryA-like)	Bradyrhizobium sp. (strain ORS278).	EryA	2	2	-	85
B1X8I0	fig|266834.1.peg.3529	-	Erythritol kinase	Rhizobium meliloti (Sinorhizobium meliloti).	EryA	3	2	-	85
B3AWW2	fig|314269.3.peg.1569	-	Putative sugar kinase	Manganese-oxidizing bacterium (strain SI85-9A1).	EryA	4	2	-	85
B4F2W7	fig|375451.6.peg.3381	-	FGGY family of carbohydrate kinases	Roseobacter denitrificans (strain ATCC 33942 / OCh 114) (Erythrobactersp. (strain OCh 114)) (Roseobacter denitrificans).	EryA	3	2	-	85
Q1YJN8	fig|204722.1.peg.2941	-	Erythritol kinase	Brucella suis.	EryA	2	2	-	85
Q38DF2	fig|266835.1.peg.5725	-	Xylulose kinase	Rhizobium loti (Mesorhizobium loti).	EryA	3	3	-	85
A4N0H8	fig|224308.1.peg.2882	Ribulokinase	-	Bacillus subtilis.	AraB	2	2	AraB	228
A5IKC5	fig|243274.1.peg.280	-	Sugar kinase, FGGY family	Thermotoga maritima.	AraB	2	1	AraB	94
B2U269	fig|316407.3.peg.63	Ribulokinase	-	Escherichia coli (strain K12).	AraB	3	2	AraB	11
C4BF18	fig|99287.1.peg.101	Ribulokinase	-	Salmonella enterica subsp. enterica serovar 4,[5],12:i:-str.CVM23701.	AraB	2	2	AraB	11
A2P9H6	fig|205913.1.peg.935	-	Putative L-ribulokinase	Bifidobacterium longum (strain DJO10A).	AraB	2	2	-	222
A2RK83	fig|279010.5.peg.2761	-	Sugar kinase, possible xylulose kinase	Bacillus licheniformis (strain DSM 13 / ATCC 14580).	AraB	4	2	-	222
A3MZ93	fig|279010.5.peg.3375	Ribulokinase	-	Bacillus licheniformis (strain DSM 13 / ATCC 14580).	AraB	4	2	-	228
A4NC34	fig|220668.1.peg.2892	-	L-ribulokinase (Putative)	Lactobacillus plantarum.	AraB	2	2	-	222
A4NMV6	fig|203123.5.peg.222	-	L-ribulokinase (Putative)	Oenococcus oeni (strain BAA-331 / PSU-1).	AraB	2	2	-	222
A4W6G7	fig|349747.3.peg.649	Ribulokinase	-	Yersinia pseudotuberculosis serotype O:1b (strain IP 31758).	AraB	4	2	-	11
A6V1L0	fig|334390.3.peg.1712	-	Sugar kinase	Lactobacillus fermentum (strain IFO 3956 / LMG 18251).	AraB	2	2	-	222
A7ZHF4	fig|331111.3.peg.2635	Ribulokinase	-	Escherichia coli O139:H28 (strain E24377A / ETEC).	AraB	3	2	-	11

A8AVX5	fig|373384.10.peg.67	Ribulokinase	-	Shigella flexneri serotype 5b (strain 8401).	AraB	4	2	-	11
A9MYN9	fig|502800.3.peg.1973	Ribulokinase	-	Yersinia pseudotuberculosis serotype IB (strain PB1/+).	AraB	4	2	-	11
B0K754	fig|411154.5.peg.661	Ribulokinase	-	Gramella forsetii (strain KT0803).	AraB	2	2	-	11
B0TWZ7	fig|585034.4.peg.64	Ribulokinase	-	Escherichia coli O8 (strain IAI1).	AraB	3	2	-	11
B1I9Z6	fig|344609.3.peg.4672	Ribulokinase	-	Shigella boydii serotype 18 (strain CDC 3083-94 / BS512).	AraB	3	2	-	11
B1XB71	fig|314283.3.peg.1782	Ribulokinase	-	Reinekea blandensis MED297.	AraB	2	2	-	11
B4SU24	fig|295319.3.peg.193	Ribulokinase	-	Salmonella paratyphi A (strain AKU_12601).	AraB	2	2	-	11
B4TJ60	fig|272994.5.peg.106	Ribulokinase	-	Salmonella paratyphi B (strain ATCC BAA-1250 / SPB7).	AraB	3	2	-	11
B5BL44	fig|550537.3.peg.106	Ribulokinase	-	Salmonella enteritidis PT4 (strain P125109).	AraB	2	2	-	11
B5F784	fig|399742.4.peg.665	Ribulokinase	-	Enterobacter sp. (strain 638).	AraB	3	2	-	11
B5FPP4	fig|203122.12.peg.770	Ribulokinase	-	Saccharophagus degradans (strain 2-40 / ATCC 43961 / DSM 17024).	AraB	3	2	-	11
B5R1U0	fig|223926.1.peg.4754	Ribulokinase	-	Vibrio parahaemolyticus.	AraB	3	3	-	11
B5RGD4	fig|209261.1.peg.105	Ribulokinase	-	Salmonella typhi.	AraB	2	2	-	11
B7L4I4	fig|198214.1.peg.56	Ribulokinase	-	Shigella flexneri.	AraB	2	2	-	11
B7M0F8	fig|340185.3.peg.4269	Ribulokinase	-	Escherichia coli B171.	AraB	5	2	-	11
B7MI36	fig|498211.3.peg.2903	Ribulokinase	-	Cellvibrio japonicus (strain Ueda107) (Pseudomonas fluorescens subsp.cellulosa).	AraB	3	3	-	11
B7UIA9	fig|216593.1.peg.3514	Ribulokinase	-	Escherichia coli O127:H6 (strain E2348/69 / EPEC).	AraB	3	2	-	11
C0T3B0	fig|439851.5.peg.201	Ribulokinase	-	Salmonella gallinarum (strain 287/91 / NCTC 13346).	AraB	2	2	-	11
C0V0B3	fig|454166.6.peg.150	Ribulokinase	-	Salmonella agona (strain SL483).	AraB	3	2	-	11

C1QVV7	fig|454169.6.peg.203	Ribulokinase	-	Salmonella heidelberg (strain SL476).	AraB	2	2	-	11
C1RPJ6	fig|349968.3.peg.2951	Ribulokinase	-	Yersinia bercovieri ATCC 43970.	AraB	3	2	-	11
C1UX26	fig|349966.3.peg.3141	Ribulokinase	-	Yersinia frederiksenii ATCC 33641.	AraB	3	2	-	11
C1W0X2	fig|349967.3.peg.418	Ribulokinase	-	Yersinia mollaretii ATCC 43969.	AraB	3	2	-	11
C1WJY8	fig|439842.7.peg.4504	Ribulokinase	-	Salmonella enterica subsp. enterica serovar Kentucky str. CDC 191.	AraB	3	2	-	11
C4DF67	fig|423368.6.peg.266	Ribulokinase	-	Salmonella newport (strain SL254).	AraB	3	2	-	11
O66131	fig|376686.6.peg.1114	Ribulokinase	-	Flavobacterium johnsoniae (strain ATCC 17061 / DSM 2064 / UW101)(Cytophaga johnsonae).	AraB	2	2	-	11
P08204	fig|300268.10.peg.245	Ribulokinase	-	Shigella boydii serotype 4 (strain Sb227).	AraB	3	2	-	11
P11553	fig|351745.7.peg.1826	-	L-ribulokinase	Shewanella sp. (strain W3-18-1).	AraB	3	2	-	11
P12011	fig|60481.10.peg.1888	Ribulokinase	-	Shewanella sp. (strain MR-7).	AraB	3	2	-	11
P18157	fig|319224.13.peg.1888	Ribulokinase	-	Shewanella putrefaciens (strain CN-32 / ATCC BAA-453).	AraB	3	2	-	11
P58541	fig|405955.9.peg.54	Ribulokinase	-	Escherichia coli (strain UTI89 / UPEC).	AraB	3	2	-	11
P58542	fig|377628.5.peg.2035	Ribulokinase	-	Yersinia pestis biovar Antiqua str. UG05-0454.	AraB	4	2	-	11
Q0T8D4	fig|344610.3.peg.4328	Ribulokinase	-	Escherichia coli 101-1.	AraB	3	2	-	11
Q1RGD6	fig|340184.3.peg.2862	Ribulokinase	-	Escherichia coli (strain 55989 / EAEC).	AraB	3	2	-	11
Q1VF74	fig|203120.4.peg.837	-	L-ribulokinase (Putative)	Leuconostoc mesenteroides subsp. mesenteroides (strain ATCC 8293 /NCDO 523).	AraB	3	2	-	222
Q1ZSC2	fig|221109.1.peg.2801	-	Hypothetical conserved protein	Oceanobacillus iheyensis.	AraB	2	2	-	222
Q2NQX8	fig|387344.13.peg.1637	-	L-ribulokinase (Putative)	Lactobacillus brevis (strain ATCC 367 / JCM 1170).	AraB	2	2	-	222

Q326H2	fig|199310.1.peg.71	Ribulokinase	-	Escherichia coli O6.	AraB	3	2	-	11
Q39D66	fig|272562.1.peg.1501	-	Sugar kinase, possible xylulose kinase	Clostridium acetobutylicum.	AraB	3	3	-	222
Q3JVT4	fig|278197.10.peg.146	-	L-ribulokinase (Putative)	Pediococcus pentosaceus (strain ATCC 25745 / 183-1w).	AraB	2	2	-	222
Q5L091	fig|313593.3.peg.3518	-	Ribulokinase	Leeuwenhoekiella blandensis MED217.	AraB	2	2	-	11
Q65M11	fig|448385.11.peg.3058	-	Ribulokinase	Sorangium cellulosum (strain So ce56) (Polyangium cellulosum (strainSo ce56)).	AraB	2	2	-	11
Q6DA23	fig|94122.5.peg.2132	Ribulokinase	-	Shewanella sp. (strain ANA-3).	AraB	3	2	-	11
Q6M8P0	fig|390874.10.peg.630	-	Carbohydrate kinase, FGGY	Thermotoga petrophila (strain RKU-1 / ATCC BAA-488 / DSM 13995).	AraB	2	1	-	94
Q83IU3	fig|60480.16.peg.1879	Ribulokinase	-	Shewanella sp. (strain MR-4).	AraB	3	2	-	11
Q83MG5	fig|340186.3.peg.4032	Ribulokinase	-	Escherichia coli E110019.	AraB	3	2	-	11
Q87FK5	fig|155864.1.peg.67	Ribulokinase	-	Escherichia coli O157:H7.	AraB	3	2	-	11
Q8FL88	fig|340197.3.peg.4346	Ribulokinase	-	Escherichia coli F11.	AraB	3	2	-	11
Q9WX53	fig|313596.3.peg.1965	Ribulokinase	-	Robiginitalea biformata (strain ATCC BAA-864 / HTCC2501 / KCTC 12146).	AraB	2	2	-	11
A4YX95	-	-	D-xylulokinase	Candida sp. Xu316.	-	-	-	XylB	87
Q0SY84	-	-	D-xylulokinase	Pichia stipitis (Yeast).	-	-	-	XylB	29
Q2GAC0	-	Xylulose kinase	-	Lactobacillus pentosus.	-	-	-	XylB	342
B8F5S3	-	-	Ribulokinase	Klebsiella pneumoniae subsp. pneumoniae (strain ATCC 700721 / MGH78578).	-	-	-	RbtK	25
A1SZE1	-	Glycerol kinase	-	Bacillus subtilis.	-	-	-	GlpK	115
A1WT61	-	Glycerol kinase	-	Thermus aquaticus.	-	-	-	GlpK	115
A7ZTB1	-	-	Glycerol kinase, glycosomal	Trypanosoma brucei.	-	-	-	GlpK	76
Q0I345	-	Glycerol kinase 2	-	Thermotoga maritima.	-	-	-	GlpK	265
Q31U85	-	Glycerol kinase	-	Thermus thermophilus.	-	-	-	GlpK	115
B7VAK0	-	-	L-ribulokinase	Corynebacterium glutamicum (Brevibacterium flavum).	-	-	-	AraB	222
