# Supplementary material for: The FGGY Carbohydrate Kinase Family: Insights into the Evolution of Functional Specificities
Source: PLoS Comput Biol. 2011 Dec 22;7(12):e1002318. doi: 10.1371/journal.pcbi.1002318 (PMC3245297; doi:10.1371/journal.pcbi.1002318)
Supplement: Table S2 — Genomic and functional context of proteins in the confidently annotated reference set (CARS). Uniprot_Acc: the Uniprot accession number of proteins; SEED_PEGid: the protein identification number in SEED; Context-based annotation: the annotation based on genomic and functional context analyses; Functional_context: the number of proteins in the genome that perform neighboring functions in a metabolic pathway of the target protein (same as in Table S1); Functional_context_PEGids: the SEED protein identification numbers of the functional context; Genomic_context (same as in Table S1): the number of proteins in a same operon of the target protein that perform neighboring functions in the metabolic pathway; Genomic_context_PEGids: the SEED protein identification numbers of the genomic context. (PDF) [file pcbi.1002318.s005.pdf]

Table S2. **Genomic and functional context of proteins in the confidently annotated reference set (CARS).** Uniprot_Acc: the Uniprot accession number of proteins; SEED_PEGid: the protein identification number in SEED database; Context-based annotation: the annotation based on genomic and functional context analyses; Functional_context: the number of proteins in the genome that perform neighboring functions in a metabolic pathway of the target protein (same as in Table S1); Functional_context_PEGids: the SEED protein identification numbers of the functional context; Genomic_context (same as in Table S1): the number of proteins in a same operon of the target protein that perform neighboring functions in the metabolic pathway; Genomic_context_PEGids: the SEED protein identification numbers of the genomic context.

Uniprot_Acc	SEED_PEGid	Context-based Annotation	Functional_context	Functional_context_PEGids	Genomic_context	Genomic_context_PEGids
C4STW2	fig|349966.3.peg.3141	AraB	3	fig|349966.3.peg.3140,fig|349966.3.peg.3142,fig|349966.3.peg.3794	2	fig|349966.3.peg.3140,fig|349966.3.peg.3142
C4SAH6	fig|349967.3.peg.418	AraB	3	fig|349967.3.peg.3509,fig|349967.3.peg.417,fig|349967.3.peg.419	2	fig|349967.3.peg.417,fig|349967.3.peg.419
C4S4W9	fig|349968.3.peg.2951	AraB	3	fig|349968.3.peg.2950,fig|349968.3.peg.2952,fig|349968.3.peg.3391	2	fig|349968.3.peg.2950,fig|349968.3.peg.2952
B5NMB4	fig|439842.7.peg.4504	AraB	3	fig|439842.7.peg.3466,fig|439842.7.peg.4502,fig|439842.7.peg.4503	2	fig|439842.7.peg.4502,fig|439842.7.peg.4503
B4SU24	fig|423368.6.peg.266	AraB	3	fig|423368.6.peg.264,fig|423368.6.peg.265,fig|423368.6.peg.3990	2	fig|423368.6.peg.264,fig|423368.6.peg.265
B5MV63	fig|99287.1.peg.101	AraB	2	fig|99287.1.peg.100,fig|99287.1.peg.99	2	fig|99287.1.peg.99,fig|99287.1.peg.100
B5RGD4	fig|439851.5.peg.201	AraB	2	fig|439851.5.peg.199,fig|439851.5.peg.200	2	fig|439851.5.peg.199,fig|439851.5.peg.200
B4TJ60	fig|454169.6.peg.203	AraB	2	fig|454169.6.peg.201,fig|454169.6.peg.202	2	fig|454169.6.peg.201,fig|454169.6.peg.202
B5F784	fig|454166.6.peg.150	AraB	3	fig|454166.6.peg.148,fig|454166.6.peg.149,fig|454166.6.peg.3783	2	fig|454166.6.peg.148,fig|454166.6.peg.149
B5BL44	fig|295319.3.peg.193	AraB	2	fig|295319.3.peg.170,fig|295319.3.peg.192	2	fig|295319.3.peg.170,fig|295319.3.peg.192
P58542	fig|209261.1.peg.105	AraB	2	fig|209261.1.peg.103,fig|209261.1.peg.104	2	fig|209261.1.peg.103,fig|209261.1.peg.104
A9MYN9	fig|272994.5.peg.106	AraB	3	fig|272994.5.peg.104,fig|272994.5.peg.105,fig|272994.5.peg.3805	2	fig|272994.5.peg.104,fig|272994.5.peg.105
A4W6G7	fig|399742.4.peg.665	AraB	3	fig|399742.4.peg.2381,fig|399742.4.peg.663,fig|399742.4.peg.664	2	fig|399742.4.peg.663,fig|399742.4.peg.664
B5R1U0	fig|550537.3.peg.106	AraB	2	fig|550537.3.peg.104,fig|550537.3.peg.105	2	fig|550537.3.peg.104,fig|550537.3.peg.105
B0GHX8	fig|377628.5.peg.2035	AraB	4	fig|377628.5.peg.2013,fig|377628.5.peg.2034,fig|377628.5.peg.2036,fig|377628.5.peg.977	2	fig|377628.5.peg.2034,fig|377628.5.peg.2036
B2K4K8	fig|502800.3.peg.1973	AraB	4	fig|502800.3.peg.1972,fig|502800.3.peg.1974,fig|502800.3.peg.1994,fig|502800.3.peg.3348	2	fig|502800.3.peg.1972,fig|502800.3.peg.1974
A7FHY3	fig|349747.3.peg.649	AraB	4	fig|349747.3.peg.3448,fig|349747.3.peg.628,fig|349747.3.peg.648,fig|349747.3.peg.650	2	fig|349747.3.peg.648,fig|349747.3.peg.650
Q87FK5	fig|223926.1.peg.4754	AraB	3	fig|223926.1.peg.4753,fig|223926.1.peg.4755,fig|223926.1.peg.4756	3	fig|223926.1.peg.4753,fig|223926.1.peg.4755,fig|223926.1.peg.4756
P58541	fig|155864.1.peg.67	AraB	3	fig|155864.1.peg.2644,fig|155864.1.peg.65,fig|155864.1.peg.66	2	fig|155864.1.peg.65,fig|155864.1.peg.66
Q1RGD6	fig|405955.9.peg.54	AraB	3	fig|405955.9.peg.1635,fig|405955.9.peg.52,fig|405955.9.peg.53	2	fig|405955.9.peg.52,fig|405955.9.peg.53
B7L4I4	fig|340184.3.peg.2862	AraB	3	fig|340184.3.peg.2863,fig|340184.3.peg.2864,fig|340184.3.peg.4761	2	fig|340184.3.peg.2863,fig|340184.3.peg.2864
Q83MG5	fig|198214.1.peg.56	AraB	2	fig|198214.1.peg.4200,fig|198214.1.peg.55	2	fig|198214.1.peg.55,fig|198214.1.peg.4200
B3IQ43	fig|340186.3.peg.4032	AraB	3	fig|340186.3.peg.3061,fig|340186.3.peg.4030,fig|340186.3.peg.4031	2	fig|340186.3.peg.4030,fig|340186.3.peg.4031
B7UIA9	fig|216593.1.peg.3514	AraB	3	fig|216593.1.peg.3515,fig|216593.1.peg.3516,fig|216593.1.peg.736	2	fig|216593.1.peg.3515,fig|216593.1.peg.3516
A7ZHF4	fig|331111.3.peg.2635	AraB	3	fig|331111.3.peg.2633,fig|331111.3.peg.2634,fig|331111.3.peg.4548	2	fig|331111.3.peg.2633,fig|331111.3.peg.2634
B3HY55	fig|340197.3.peg.4346	AraB	3	fig|340197.3.peg.3086,fig|340197.3.peg.4347,fig|340197.3.peg.4348	2	fig|340197.3.peg.4347,fig|340197.3.peg.4348
Q8FL88	fig|199310.1.peg.71	AraB	3	fig|199310.1.peg.2249,fig|199310.1.peg.69,fig|199310.1.peg.70	2	fig|199310.1.peg.69,fig|199310.1.peg.70
Q326H2	fig|300268.10.peg.245	AraB	3	fig|300268.10.peg.1442,fig|300268.10.peg.243,fig|300268.10.peg.244	2	fig|300268.10.peg.243,fig|300268.10.peg.244
B3XIM5	fig|344610.3.peg.4328	AraB	3	fig|344610.3.peg.3002,fig|344610.3.peg.4329,fig|344610.3.peg.4330	2	fig|344610.3.peg.4329,fig|344610.3.peg.4330
P08204	fig|316407.3.peg.63	AraB	3	fig|316407.3.peg.1855,fig|316407.3.peg.61,fig|316407.3.peg.62	2	fig|316407.3.peg.61,fig|316407.3.peg.62

B3WR88	fig|340185.3.peg.4269	AraB	5	fig|340185.3.peg.3866,fig|340185.3.peg.3867,fig|340185.3.peg.3868,fig|340185.3.peg.4270,fig|340185.3.peg.4271	2	fig|340185.3.peg.4270,fig|340185.3.peg.4271
Q0T8D4	fig|373384.10.peg.67	AraB	4	fig|373384.10.peg.2225,fig|373384.10.peg.4444,fig|373384.10.peg.65,fig|373384.10.peg.66	2	fig|373384.10.peg.65,fig|373384.10.peg.66
B2U269	fig|344609.3.peg.4672	AraB	3	fig|344609.3.peg.4670,fig|344609.3.peg.4671,fig|344609.3.peg.4762	2	fig|344609.3.peg.4670,fig|344609.3.peg.4671
B7M0F8	fig|585034.4.peg.64	AraB	3	fig|585034.4.peg.1964,fig|585034.4.peg.62,fig|585034.4.peg.63	2	fig|585034.4.peg.62,fig|585034.4.peg.63
A0LZ79	fig|411154.5.peg.661	AraB	2	fig|411154.5.peg.662,fig|411154.5.peg.663	2	fig|411154.5.peg.662,fig|411154.5.peg.663
A3XLT3	fig|313593.3.peg.3518	AraB	2	fig|313593.3.peg.3515,fig|313593.3.peg.3517	2	fig|313593.3.peg.3515,fig|313593.3.peg.3517
A9GMQ4	fig|448385.11.peg.3058	AraB	2	fig|448385.11.peg.3056,fig|448385.11.peg.3057	2	fig|448385.11.peg.3056,fig|448385.11.peg.3057
A5FKW1	fig|376686.6.peg.1114	AraB	2	fig|376686.6.peg.1115,fig|376686.6.peg.1116	2	fig|376686.6.peg.1115,fig|376686.6.peg.1116
A4CJN0	fig|313596.3.peg.1965	AraB	2	fig|313596.3.peg.1963,fig|313596.3.peg.1964	2	fig|313596.3.peg.1963,fig|313596.3.peg.1964
A4Y756	fig|319224.13.peg.1888	AraB	3	fig|319224.13.peg.1879,fig|319224.13.peg.1886,fig|319224.13.peg.1887	2	fig|319224.13.peg.1886,fig|319224.13.peg.1887
A1RJD4	fig|351745.7.peg.1826	AraB	3	fig|351745.7.peg.1827,fig|351745.7.peg.1828,fig|351745.7.peg.1835	2	fig|351745.7.peg.1827,fig|351745.7.peg.1828
C1Z4Y4	fig|485917.5.peg.1125	AraB	2	fig|485917.5.peg.1123,fig|485917.5.peg.1124	2	fig|485917.5.peg.1123,fig|485917.5.peg.1124
Q0HV69	fig|60481.10.peg.1888	AraB	3	fig|60481.10.peg.1879,fig|60481.10.peg.1886,fig|60481.10.peg.1887	2	fig|60481.10.peg.1886,fig|60481.10.peg.1887
A0KWX5	fig|94122.5.peg.2132	AraB	3	fig|94122.5.peg.2133,fig|94122.5.peg.2134,fig|94122.5.peg.2141	2	fig|94122.5.peg.2133,fig|94122.5.peg.2134
Q0HIR7	fig|60480.16.peg.1879	AraB	3	fig|60480.16.peg.1880,fig|60480.16.peg.1881,fig|60480.16.peg.1889	2	fig|60480.16.peg.1880,fig|60480.16.peg.1881
B3PD59	fig|498211.3.peg.2903	AraB	3	fig|498211.3.peg.2901,fig|498211.3.peg.2902,fig|498211.3.peg.2905	3	fig|498211.3.peg.2901,fig|498211.3.peg.2902,fig|498211.3.peg.2905
Q21MP5	fig|203122.12.peg.770	AraB	3	fig|203122.12.peg.764,fig|203122.12.peg.771,fig|203122.12.peg.772	2	fig|203122.12.peg.771,fig|203122.12.peg.772
C1U043	fig|471854.4.peg.5664	AraB	2	fig|471854.4.peg.5665,fig|471854.4.peg.5666	2	fig|471854.4.peg.5665,fig|471854.4.peg.5666
A4BF97	fig|314283.3.peg.1782	AraB	2	fig|314283.3.peg.1780,fig|314283.3.peg.1783	2	fig|314283.3.peg.1780,fig|314283.3.peg.1783
C4BFM5	fig|446465.4.peg.714	AraB	2	fig|446465.4.peg.715,fig|446465.4.peg.719	2	fig|446465.4.peg.715,fig|446465.4.peg.719
B3DTJ7	fig|205913.1.peg.935	AraB	2	fig|205913.1.peg.936,fig|205913.1.peg.937	2	fig|205913.1.peg.936,fig|205913.1.peg.937
C1W5M4	fig|471856.4.peg.1744	AraB	2	fig|471856.4.peg.1742,fig|471856.4.peg.1743	2	fig|471856.4.peg.1742,fig|471856.4.peg.1743
Q8EMP2	fig|221109.1.peg.2801	AraB	2	fig|221109.1.peg.2799,fig|221109.1.peg.2800	2	fig|221109.1.peg.2799,fig|221109.1.peg.2800
Q03XW0	fig|203120.4.peg.837	AraB	3	fig|203120.4.peg.1913,fig|203120.4.peg.835,fig|203120.4.peg.838	2	fig|203120.4.peg.835,fig|203120.4.peg.838
Q65J12	fig|279010.5.peg.2761	AraB	4	fig|279010.5.peg.2762,fig|279010.5.peg.2763,fig|279010.5.peg.3374,fig|279010.5.peg.3376	2	fig|279010.5.peg.2762,fig|279010.5.peg.2763
B2GE12	fig|334390.3.peg.1712	AraB	2	fig|334390.3.peg.1708,fig|334390.3.peg.1711	2	fig|334390.3.peg.1708,fig|334390.3.peg.1711
Q97JE2	fig|272562.1.peg.1501	AraB	3	fig|272562.1.peg.1498,fig|272562.1.peg.1499,fig|272562.1.peg.1503	3	fig|272562.1.peg.1498,fig|272562.1.peg.1499,fig|272562.1.peg.1503
Q03PR3	fig|387344.13.peg.1637	AraB	2	fig|387344.13.peg.1635,fig|387344.13.peg.1636	2	fig|387344.13.peg.1635,fig|387344.13.peg.1636
Q88S82	fig|220668.1.peg.2892	AraB	2	fig|220668.1.peg.2890,fig|220668.1.peg.2891	2	fig|220668.1.peg.2890,fig|220668.1.peg.2891
Q04H58	fig|203123.5.peg.222	AraB	2	fig|203123.5.peg.223,fig|203123.5.peg.224	2	fig|203123.5.peg.223,fig|203123.5.peg.224
Q03HQ2	fig|278197.10.peg.146	AraB	2	fig|278197.10.peg.147,fig|278197.10.peg.148	2	fig|278197.10.peg.147,fig|278197.10.peg.148
P94524	fig|224308.1.peg.2882	AraB	2	fig|224308.1.peg.2881,fig|224308.1.peg.2883	2	fig|224308.1.peg.2881,fig|224308.1.peg.2883
Q65GC1	fig|279010.5.peg.3375	AraB	4	fig|279010.5.peg.2762,fig|279010.5.peg.2763,fig|279010.5.peg.3374,fig|279010.5.peg.3376	2	fig|279010.5.peg.2762,fig|279010.5.peg.2763,fig|279010.5.peg.3374,fig|279010.5.peg.3376
A5IKC5	fig|390874.10.peg.630	AraB	2	fig|390874.10.peg.631,fig|390874.10.peg.650	1	fig|390874.10.peg.631
Q9WYC0	fig|243274.1.peg.280	AraB	2	fig|243274.1.peg.273,fig|243274.1.peg.279	1	fig|243274.1.peg.279
Q1YJN8	fig|314269.3.peg.1569	EryA	4	fig|314269.3.peg.1560,fig|314269.3.peg.1567,fig|314269.3.peg.1574,fig|314269.3.peg.507	2	fig|314269.3.peg.1567,fig|314269.3.peg.1574

A5EGP3	fig|288000.5.peg.3260	EryA	2	fig|288000.5.peg.3255,fig|288000.5.peg.3263	2	fig|288000.5.peg.3255,fig|288000.5.peg.3263
A4YX95	fig|114615.3.peg.4624	EryA	2	fig|114615.3.peg.4621,fig|114615.3.peg.4629	2	fig|114615.3.peg.4621,fig|114615.3.peg.4629
Q92NH0	fig|266834.1.peg.3529	EryA	3	fig|266834.1.peg.3522,fig|266834.1.peg.3526,fig|266834.1.peg.3534	2	fig|266834.1.peg.3526,fig|266834.1.peg.3534
Q162I2	fig|375451.6.peg.3381	EryA	3	fig|375451.6.peg.3374,fig|375451.6.peg.3378,fig|375451.6.peg.3386	2	fig|375451.6.peg.3378,fig|375451.6.peg.3386
C4IV00	fig|262698.3.peg.2303	EryA	2	fig|262698.3.peg.2301,fig|262698.3.peg.2302	2	fig|262698.3.peg.2301,fig|262698.3.peg.2302
Q8YCU8	fig|224914.1.peg.2490	EryA	2	fig|224914.1.peg.2488,fig|224914.1.peg.2489	2	fig|224914.1.peg.2488,fig|224914.1.peg.2489
Q2YIQ1	fig|430066.3.peg.382	EryA	1	fig|430066.3.peg.381	1	fig|430066.3.peg.381
Q986P3	fig|266835.1.peg.5725	EryA	3	fig|266835.1.peg.5720,fig|266835.1.peg.5727,fig|266835.1.peg.5729	3	fig|266835.1.peg.5720,fig|266835.1.peg.5727,fig|266835.1.peg.5729
Q8FVH7	fig|204722.1.peg.2941	EryA	2	fig|204722.1.peg.2942,fig|204722.1.peg.2943	2	fig|204722.1.peg.2942,fig|204722.1.peg.2943
A9WZF0	fig|470137.3.peg.826	EryA	2	fig|470137.3.peg.827,fig|470137.3.peg.828	2	fig|470137.3.peg.827,fig|470137.3.peg.828
Q04I07	fig|171101.1.peg.2172	FucK	3	fig|171101.1.peg.1961,fig|171101.1.peg.1968,fig|171101.1.peg.2275	2	fig|171101.1.peg.1968,fig|171101.1.peg.2275
B1S255	fig|453362.3.peg.1376	FucK	3	fig|453362.3.peg.1377,fig|453362.3.peg.1378,fig|453362.3.peg.1385	2	fig|453362.3.peg.1377,fig|453362.3.peg.1378
A5M1U5	fig|406559.4.peg.1306	FucK	3	fig|406559.4.peg.1307,fig|406559.4.peg.1308,fig|406559.4.peg.1315	2	fig|406559.4.peg.1307,fig|406559.4.peg.1308
B1I9X7	fig|453365.3.peg.1417	FucK	3	fig|453365.3.peg.1418,fig|453365.3.peg.1419,fig|453365.3.peg.1426	2	fig|453365.3.peg.1418,fig|453365.3.peg.1419
A5MU04	fig|406563.4.peg.788	FucK	3	fig|406563.4.peg.779,fig|406563.4.peg.786,fig|406563.4.peg.787	2	fig|406563.4.peg.786,fig|406563.4.peg.787
A5LLM3	fig|406557.4.peg.932	FucK	3	fig|406557.4.peg.923,fig|406557.4.peg.930,fig|406557.4.peg.931	2	fig|406557.4.peg.930,fig|406557.4.peg.931
B2DUN0	fig|453364.8.peg.1218	FucK	3	fig|453364.8.peg.1209,fig|453364.8.peg.1216,fig|453364.8.peg.1217	2	fig|453364.8.peg.1216,fig|453364.8.peg.1217
Q8XNL6	fig|195102.1.peg.380	FucK	2	fig|195102.1.peg.381,fig|195102.1.peg.382	2	fig|195102.1.peg.381,fig|195102.1.peg.382
Q97N88	fig|170187.1.peg.2028	FucK	3	fig|170187.1.peg.2019,fig|170187.1.peg.2026,fig|170187.1.peg.2027	2	fig|170187.1.peg.2026,fig|170187.1.peg.2027
A5LR06	fig|1313.3.peg.1747	FucK	4	fig|1313.3.peg.1741,fig|1313.3.peg.1746,fig|1313.3.peg.855,fig|1313.3.peg.856	1	fig|1313.3.peg.1746
B3IQX1	fig|340186.3.peg.4322	FucK	3	fig|340186.3.peg.4321,fig|340186.3.peg.4323,fig|340186.3.peg.4325	3	fig|340186.3.peg.4321,fig|340186.3.peg.4323,fig|340186.3.peg.4325
B3HPH9	fig|340197.3.peg.1264	FucK	3	fig|340197.3.peg.1263,fig|340197.3.peg.1265,fig|340197.3.peg.1267	3	fig|340197.3.peg.1263,fig|340197.3.peg.1265,fig|340197.3.peg.1267
C3SWV3	fig|155864.1.peg.3666	FucK	3	fig|155864.1.peg.3663,fig|155864.1.peg.3665,fig|155864.1.peg.3667	3	fig|155864.1.peg.3663,fig|155864.1.peg.3665,fig|155864.1.peg.3667
Q3YY54	fig|216599.1.peg.1785	FucK	3	fig|216599.1.peg.1782,fig|216599.1.peg.1784,fig|216599.1.peg.1786	3	fig|216599.1.peg.1782,fig|216599.1.peg.1784,fig|216599.1.peg.1786
B7MLC4	fig|405955.9.peg.2580	FucK	3	fig|405955.9.peg.2577,fig|405955.9.peg.2579,fig|405955.9.peg.2581	3	fig|405955.9.peg.2577,fig|405955.9.peg.2579,fig|405955.9.peg.2581
P11553	fig|83333.1.peg.2759	FucK	3	fig|83333.1.peg.2756,fig|83333.1.peg.2758,fig|83333.1.peg.2760	3	fig|83333.1.peg.2756,fig|83333.1.peg.2758,fig|83333.1.peg.2760
B7UHL9	fig|216593.1.peg.1270	FucK	3	fig|216593.1.peg.1266,fig|216593.1.peg.1269,fig|216593.1.peg.1271	3	fig|216593.1.peg.1266,fig|216593.1.peg.1269,fig|216593.1.peg.1271
Q32CB5	fig|216598.1.peg.49	FucK	3	fig|216598.1.peg.47,fig|216598.1.peg.50,fig|216598.1.peg.54	2	fig|216598.1.peg.47,fig|216598.1.peg.50
B2UN37	fig|349741.3.peg.1944	FucK	2	fig|349741.3.peg.1946,fig|349741.3.peg.2237	1	fig|349741.3.peg.1946
B3YJZ6	fig|439842.7.peg.2718	FucK	3	fig|439842.7.peg.2714,fig|439842.7.peg.2717,fig|439842.7.peg.2719	3	fig|439842.7.peg.2714,fig|439842.7.peg.2717,fig|439842.7.peg.2719
B4TGN4	fig|99287.1.peg.2872	FucK	3	fig|99287.1.peg.2870,fig|99287.1.peg.2871,fig|99287.1.peg.2873	3	fig|99287.1.peg.2870,fig|99287.1.peg.2871,fig|99287.1.peg.2873
B5FTY2	fig|550538.3.peg.3066	FucK	3	fig|550538.3.peg.3062,fig|550538.3.peg.3065,fig|550538.3.peg.3067	3	fig|550538.3.peg.3062,fig|550538.3.peg.3065,fig|550538.3.peg.3067
B5CBW6	fig|439843.6.peg.3147	FucK	3	fig|439843.6.peg.3142,fig|439843.6.peg.3146,fig|439843.6.peg.3148	3	fig|439843.6.peg.3142,fig|439843.6.peg.3146,fig|439843.6.peg.3148

A6TD84	fig|272620.3.peg.3157	FucK	3	fig|272620.3.peg.3154,fig|272620.3.peg.3156,fig|272620.3.peg.3158	3	fig|272620.3.peg.3154,fig|272620.3.peg.3156,fig|272620.3.peg.3158
B5F4S4	fig|454166.6.peg.3047	FucK	3	fig|454166.6.peg.3042,fig|454166.6.peg.3046,fig|454166.6.peg.3048	3	fig|454166.6.peg.3042,fig|454166.6.peg.3046,fig|454166.6.peg.3048
Q5PEK8	fig|554290.7.peg.2999	FucK	3	fig|554290.7.peg.2994,fig|554290.7.peg.2998,fig|554290.7.peg.3000	3	fig|554290.7.peg.2994,fig|554290.7.peg.2998,fig|554290.7.peg.3000
Q8Z428	fig|220341.1.peg.2762	FucK	3	fig|220341.1.peg.2759,fig|220341.1.peg.2761,fig|220341.1.peg.2763	3	fig|220341.1.peg.2759,fig|220341.1.peg.2761,fig|220341.1.peg.2763
A8AP16	fig|290338.6.peg.3495	FucK	3	fig|290338.6.peg.3492,fig|290338.6.peg.3494,fig|290338.6.peg.3496	3	fig|290338.6.peg.3492,fig|290338.6.peg.3494,fig|290338.6.peg.3496
B4T4X2	fig|423368.6.peg.3277	FucK	3	fig|423368.6.peg.3272,fig|423368.6.peg.3276,fig|423368.6.peg.3278	3	fig|423368.6.peg.3272,fig|423368.6.peg.3276,fig|423368.6.peg.3278
A9N2J3	fig|272994.5.peg.3090	FucK	3	fig|272994.5.peg.3085,fig|272994.5.peg.3089,fig|272994.5.peg.3091	3	fig|272994.5.peg.3085,fig|272994.5.peg.3089,fig|272994.5.peg.3091
A8AVX5	fig|467705.8.peg.609	GlpK	2	fig|467705.8.peg.164,fig|467705.8.peg.607	1	fig|467705.8.peg.607
B2DHW6	fig|406557.4.peg.2267	GlpK	3	fig|406557.4.peg.1826,fig|406557.4.peg.2269,fig|406557.4.peg.949	1	fig|406557.4.peg.2269
B1S281	fig|453363.3.peg.1184	GlpK	2	fig|453363.3.peg.1082,fig|453363.3.peg.1182	1	fig|453363.3.peg.1182
B1I9Z6	fig|487214.3.peg.2311	GlpK	2	fig|487214.3.peg.2208,fig|487214.3.peg.2309	1	fig|487214.3.peg.2309
Q5WIL1	fig|66692.3.peg.924	GlpK	4	fig|66692.3.peg.1872,fig|66692.3.peg.1922,fig|66692.3.peg.3362,fig|66692.3.peg.925	1	fig|66692.3.peg.3362,fig|66692.3.peg.925
B0TWZ7	fig|484022.4.peg.1045	GlpK	3	fig|484022.4.peg.1046,fig|484022.4.peg.1047,fig|484022.4.peg.446	2	fig|484022.4.peg.1046,fig|484022.4.peg.1047
A8FBF0	fig|315750.5.peg.795	GlpK	3	fig|315750.5.peg.1832,fig|315750.5.peg.794,fig|315750.5.peg.796	2	fig|315750.5.peg.794,fig|315750.5.peg.796
Q5WCJ3	fig|66692.3.peg.3363	GlpK	4	fig|66692.3.peg.1872,fig|66692.3.peg.1922,fig|66692.3.peg.3362,fig|66692.3.peg.925	1	fig|66692.3.peg.3362
B4BKR7	fig|420246.5.peg.1187	GlpK	3	fig|420246.5.peg.1186,fig|420246.5.peg.1997,fig|420246.5.peg.2060	1	fig|420246.5.peg.1186
B0K754	fig|340099.4.peg.528	GlpK	3	fig|340099.4.peg.527,fig|340099.4.peg.529,fig|340099.4.peg.825	2	fig|340099.4.peg.527,fig|340099.4.peg.529
Q5L091	fig|235909.3.peg.209	GlpK	3	fig|235909.3.peg.1030,fig|235909.3.peg.1117,fig|235909.3.peg.208	1	fig|235909.3.peg.208
Q65M11	fig|279010.5.peg.1669	GlpK	3	fig|279010.5.peg.1668,fig|279010.5.peg.1670,fig|279010.5.peg.269	2	fig|279010.5.peg.1668,fig|279010.5.peg.1670
B1YKL3	fig|262543.4.peg.718	GlpK	5	fig|262543.4.peg.1099,fig|262543.4.peg.1808,fig|262543.4.peg.210,fig|262543.4.peg.717,fig|262543.4.peg.719	2	fig|262543.4.peg.1099,fig|262543.4.peg.717,fig|262543.4.peg.719
Q1R3Z2	fig|405955.9.peg.3694	GlpK	5	fig|405955.9.peg.2015,fig|405955.9.peg.2016,fig|405955.9.peg.3218,fig|405955.9.peg.3391,fig|405955.9.peg.3695	1	fig|405955.9.peg.3695
C1RTJ4	fig|485918.5.peg.2857	GlpK	3	fig|485918.5.peg.2615,fig|485918.5.peg.2856,fig|485918.5.peg.2858	2	fig|485918.5.peg.2856,fig|485918.5.peg.2858
B4F166	fig|584.1.peg.1407	GlpK	5	fig|584.1.peg.1406,fig|584.1.peg.30,fig|584.1.peg.3469,fig|584.1.peg.3540,fig|584.1.peg.3541	1	fig|584.1.peg.1406
A1AIA2	fig|155864.1.peg.4863	GlpK	5	fig|155864.1.peg.3125,fig|155864.1.peg.3126,fig|155864.1.peg.4277,fig|155864.1.peg.4499,fig|155864.1.peg.4864	1	fig|155864.1.peg.4864
Q6LVI8	fig|298386.1.peg.2471	GlpK	5	fig|298386.1.peg.2382,fig|298386.1.peg.2450,fig|298386.1.peg.2472,fig|298386.1.peg.3625,fig|298386.1.peg.3626	1	fig|298386.1.peg.2472
B1JQ85	fig|349746.3.peg.2939	GlpK	6	fig|349746.3.peg.1206,fig|349746.3.peg.2760,fig|349746.3.peg.2940,fig|349746.3.peg.3442,fig|349746.3.peg.3443,fig|349746.3.peg.3444	1	fig|349746.3.peg.2940
C4SS63	fig|349966.3.peg.3323	GlpK	5	fig|349966.3.peg.1171,fig|349966.3.peg.2643,fig|349966.3.peg.2644,fig|349966.3.peg.3302,fig|349966.3.peg.3324	1	fig|349966.3.peg.3324
C4S6A1	fig|349968.3.peg.2516	GlpK	5	fig|349968.3.peg.125,fig|349968.3.peg.1448,fig|349968.3.peg.1984,fig|349968.3.peg.1985,fig|349968.3.peg.2515	1	fig|349968.3.peg.2515
A1WT61	fig|349124.5.peg.78	GlpK	2	fig|349124.5.peg.1194,fig|349124.5.peg.79	1	fig|349124.5.peg.79

A1JI03	fig|630.2.peg.98	GlpK	5	fig|630.2.peg.207,fig|630.2.peg.208,fig|630.2.peg.3936,fig|630.2.peg.81,fig|630.2.peg.99	1	fig|630.2.peg.99
C4T5B8	fig|349965.3.peg.2953	GlpK	5	fig|349965.3.peg.1630,fig|349965.3.peg.1784,fig|349965.3.peg.1785,fig|349965.3.peg.2930,fig|349965.3.peg.2954	1	fig|349965.3.peg.2954
A3UPP4	fig|314291.3.peg.4041	GlpK	3	fig|314291.3.peg.216,fig|314291.3.peg.4040,fig|314291.3.peg.4043	2	fig|314291.3.peg.4040,fig|314291.3.peg.4043
A1SZE1	fig|357804.5.peg.2985	GlpK	3	fig|357804.5.peg.2983,fig|357804.5.peg.3023,fig|357804.5.peg.3048	1	fig|357804.5.peg.2983
A3XSV7	fig|314290.3.peg.1506	GlpK	3	fig|314290.3.peg.1505,fig|314290.3.peg.1508,fig|314290.3.peg.3143	2	fig|314290.3.peg.1505,fig|314290.3.peg.1508
Q1YZX9	fig|314280.3.peg.5317	GlpK	5	fig|314280.3.peg.2594,fig|314280.3.peg.2595,fig|314280.3.peg.4421,fig|314280.3.peg.4508,fig|314280.3.peg.5316	1	fig|314280.3.peg.5316
Q2S6Z4	fig|349521.5.peg.6106	GlpK	4	fig|349521.5.peg.2462,fig|349521.5.peg.3667,fig|349521.5.peg.4044,fig|349521.5.peg.6105	1	fig|349521.5.peg.6105
B1YE68	fig|262543.4.peg.1098	GlpK	5	fig|262543.4.peg.1099,fig|262543.4.peg.1808,fig|262543.4.peg.210,fig|262543.4.peg.717,fig|262543.4.peg.719	1	fig|262543.4.peg.1099
Q7MI93	fig|196600.1.peg.2692	GlpK	5	fig|196600.1.peg.2691,fig|196600.1.peg.3153,fig|196600.1.peg.3847,fig|196600.1.peg.5099,fig|196600.1.peg.5135	2	fig|196600.1.peg.2691,fig|196600.1.peg.5099
B7VAK0	fig|208963.3.peg.1118	GlpK	4	fig|208963.3.peg.1117,fig|208963.3.peg.1120,fig|208963.3.peg.3220,fig|208963.3.peg.593	2	fig|208963.3.peg.1117,fig|208963.3.peg.1120,fig|208963.3.peg.1117,fig|208963.3.peg.1120
A7K371	fig|150340.3.peg.367	GlpK	3	fig|150340.3.peg.1218,fig|150340.3.peg.365,fig|150340.3.peg.368	2	fig|150340.3.peg.365,fig|150340.3.peg.368
A2P9H6	fig|88888881.3.peg.2620	GlpK	5	fig|88888881.3.peg.1470,fig|88888881.3.peg.2622,fig|88888881.3.peg.2623,fig|88888881.3.peg.2624,fig|88888881.3.peg.3615	3	fig|88888881.3.peg.2622,fig|88888881.3.peg.2623,fig|88888881.3.peg.2624
A2PPL3	fig|412883.3.peg.366	GlpK	5	fig|412883.3.peg.2508,fig|412883.3.peg.367,fig|412883.3.peg.368,fig|412883.3.peg.369,fig|412883.3.peg.457	3	fig|412883.3.peg.367,fig|412883.3.peg.368,fig|412883.3.peg.369
Q87M72	fig|223926.1.peg.2386	GlpK	3	fig|223926.1.peg.2385,fig|223926.1.peg.2388,fig|223926.1.peg.2832	2	fig|223926.1.peg.2385,fig|223926.1.peg.2388
A7N1R1	fig|338187.4.peg.2422	GlpK	3	fig|338187.4.peg.135,fig|338187.4.peg.2421,fig|338187.4.peg.2424	2	fig|338187.4.peg.2421,fig|338187.4.peg.2424
Q9KLJ9	fig|345072.3.peg.1663	GlpK	5	fig|345072.3.peg.1587,fig|345072.3.peg.1664,fig|345072.3.peg.1665,fig|345072.3.peg.1666,fig|345072.3.peg.2462	3	fig|345072.3.peg.1664,fig|345072.3.peg.1665,fig|345072.3.peg.1666
Q51390	fig|208964.1.peg.3582	GlpK	4	fig|208964.1.peg.1615,fig|208964.1.peg.3025,fig|208964.1.peg.3581,fig|208964.1.peg.3584	2	fig|208964.1.peg.3581,fig|208964.1.peg.3584,fig|208964.1.peg.3581,fig|208964.1.peg.3584
Q1ZSC2	fig|314292.13.peg.2707	GlpK	3	fig|314292.13.peg.2706,fig|314292.13.peg.2709,fig|314292.13.peg.884	2	fig|314292.13.peg.2706,fig|314292.13.peg.2709
Q1VF74	fig|314288.3.peg.430	GlpK	3	fig|314288.3.peg.3916,fig|314288.3.peg.428,fig|314288.3.peg.431	2	fig|314288.3.peg.428,fig|314288.3.peg.431
A2RK83	fig|416870.7.peg.1098	GlpK	7	fig|416870.7.peg.1096,fig|416870.7.peg.1097,fig|416870.7.peg.1113,fig|416870.7.peg.2312,fig|416870.7.peg.250,fig|416870.7.peg.251,fig|416870.7.peg.860	2	fig|416870.7.peg.1096,fig|416870.7.peg.1097
A6V1L0	fig|381754.5.peg.1503	GlpK	4	fig|381754.5.peg.1501,fig|381754.5.peg.1504,fig|381754.5.peg.2048,fig|381754.5.peg.3491	2	fig|381754.5.peg.1501,fig|381754.5.peg.1504
Q39D66	fig|269483.3.peg.7487	GlpK	4	fig|269483.3.peg.2086,fig|269483.3.peg.7486,fig|269483.3.peg.7488,fig|269483.3.peg.7663	2	fig|269483.3.peg.2086,fig|269483.3.peg.7486,fig|269483.3.peg.7488
B6ER09	fig|316275.9.peg.3623	GlpK	5	fig|316275.9.peg.2927,fig|316275.9.peg.3622,fig|316275.9.peg.3625,fig|316275.9.peg.3635,fig|316275.9.peg.3636	2	fig|316275.9.peg.3622,fig|316275.9.peg.3625
Q2NQX8	fig|343509.6.peg.4788	GlpK	5	fig|343509.6.peg.3963,fig|343509.6.peg.4786,fig|343509.6.peg.4787,fig|343509.6.peg.4811,fig|343509.6.peg.5057	2	fig|343509.6.peg.4786,fig|343509.6.peg.4787
B5ET10	fig|388396.7.peg.3479	GlpK	5	fig|388396.7.peg.3469,fig|388396.7.peg.3470,fig|388396.7.peg.3477,fig|388396.7.peg.3480,fig|388396.7.peg.379	2	fig|388396.7.peg.3477,fig|388396.7.peg.3480

Q5E0Z0	fig|312309.3.peg.2894	GlpK	5	fig|312309.3.peg.2348,fig|312309.3.peg.2893,fig|312309.3.peg.2897,fig|312309.3.peg.2907,fig|312309.3.peg.2908	2	fig|312309.3.peg.2893,fig|312309.3.peg.2897
A4NC34	fig|262727.1.peg.1467	GlpK	4	fig|262727.1.peg.1250,fig|262727.1.peg.1468,fig|262727.1.peg.1472,fig|262727.1.peg.1473	1	fig|262727.1.peg.1468
A4NMV6	fig|71421.1.peg.661	GlpK	4	fig|71421.1.peg.574,fig|71421.1.peg.654,fig|71421.1.peg.655,fig|71421.1.peg.660	1	fig|71421.1.peg.660
Q3JVT4	fig|320372.3.peg.3613	GlpK	3	fig|320372.3.peg.3164,fig|320372.3.peg.3612,fig|320372.3.peg.3614	2	fig|320372.3.peg.3612,fig|320372.3.peg.3614
A4N0H8	fig|374931.9.peg.1335	GlpK	8	fig|374931.9.peg.1327,fig|374931.9.peg.1328,fig|374931.9.peg.1329,fig|374931.9.peg.1330,fig|374931.9.peg.1334,fig|374931.9.peg.1378,fig|374931.9.peg.1379,fig|374931.9.peg.1725	2	fig|374931.9.peg.1330,fig|374931.9.peg.1334
A3MZ93	fig|416269.5.peg.361	GlpK	4	fig|416269.5.peg.1433,fig|416269.5.peg.360,fig|416269.5.peg.365,fig|416269.5.peg.366	3	fig|416269.5.peg.360,fig|416269.5.peg.365,fig|416269.5.peg.366
B3H0N9	fig|434271.3.peg.395	GlpK	5	fig|434271.3.peg.1522,fig|434271.3.peg.394,fig|434271.3.peg.400,fig|434271.3.peg.401,fig|434271.3.peg.402	2	fig|434271.3.peg.394,fig|434271.3.peg.400
A5UE44	fig|374930.8.peg.1737	GlpK	7	fig|374930.8.peg.1727,fig|374930.8.peg.1738,fig|374930.8.peg.1739,fig|374930.8.peg.1742,fig|374930.8.peg.1743,fig|374930.8.peg.1744,fig|374930.8.peg.397	3	fig|374930.8.peg.1738,fig|374930.8.peg.1739,fig|374930.8.peg.1742
B2TWC2	fig|344609.3.peg.3562	GlpK	5	fig|344609.3.peg.2328,fig|344609.3.peg.2329,fig|344609.3.peg.317,fig|344609.3.peg.3561,fig|344609.3.peg.86	1	fig|344609.3.peg.3561
B5BJK3	fig|550537.3.peg.4131	GlpK	5	fig|550537.3.peg.2408,fig|550537.3.peg.2409,fig|550537.3.peg.3563,fig|550537.3.peg.3750,fig|550537.3.peg.4132	1	fig|550537.3.peg.4132
B3BWR9	fig|511145.6.peg.4026	GlpK	6	fig|511145.6.peg.2314,fig|511145.6.peg.2315,fig|511145.6.peg.2316,fig|511145.6.peg.3504,fig|511145.6.peg.3709,fig|511145.6.peg.4027	1	fig|511145.6.peg.4027
B7NU81	fig|585057.4.peg.3176	GlpK	6	fig|585057.4.peg.2480,fig|585057.4.peg.2481,fig|585057.4.peg.2482,fig|585057.4.peg.3175,fig|585057.4.peg.4036,fig|585057.4.peg.4271	1	fig|585057.4.peg.3175
A5MG16	fig|406561.4.peg.1158	GlpK	2	fig|406561.4.peg.1160,fig|406561.4.peg.1257	1	fig|406561.4.peg.1160
A8AL00	fig|290338.6.peg.2577	GlpK	6	fig|290338.6.peg.2576,fig|290338.6.peg.4059,fig|290338.6.peg.4241,fig|290338.6.peg.460,fig|290338.6.peg.461,fig|290338.6.peg.462	1	fig|290338.6.peg.2576
A5LQY3	fig|406558.4.peg.25	GlpK	2	fig|406558.4.peg.118,fig|406558.4.peg.27	1	fig|406558.4.peg.27
A4WG72	fig|399742.4.peg.3849	GlpK	5	fig|399742.4.peg.210,fig|399742.4.peg.2684,fig|399742.4.peg.2685,fig|399742.4.peg.3640,fig|399742.4.peg.3848	1	fig|399742.4.peg.3848
A4IW85	fig|418136.4.peg.209	GlpK	3	fig|418136.4.peg.1254,fig|418136.4.peg.212,fig|418136.4.peg.213	2	fig|418136.4.peg.212,fig|418136.4.peg.213
Q5NIE5	fig|393115.8.peg.138	GlpK	3	fig|393115.8.peg.141,fig|393115.8.peg.142,fig|393115.8.peg.936	2	fig|393115.8.peg.141,fig|393115.8.peg.142
B7M6X7	fig|585034.4.peg.4025	GlpK	6	fig|585034.4.peg.2286,fig|585034.4.peg.2287,fig|585034.4.peg.2288,fig|585034.4.peg.3488,fig|585034.4.peg.3698,fig|585034.4.peg.4026	1	fig|585034.4.peg.4026
B5NTU6	fig|439842.7.peg.3882	GlpK	5	fig|439842.7.peg.2090,fig|439842.7.peg.2091,fig|439842.7.peg.3306,fig|439842.7.peg.3490,fig|439842.7.peg.3883	1	fig|439842.7.peg.3883
B8F5S3	fig|557723.7.peg.1050	GlpK	5	fig|557723.7.peg.1051,fig|557723.7.peg.1293,fig|557723.7.peg.1294,fig|557723.7.peg.1295,fig|557723.7.peg.1483	1	fig|557723.7.peg.1051
B2SF32	fig|441952.3.peg.234	GlpK	3	fig|441952.3.peg.237,fig|441952.3.peg.238,fig|441952.3.peg.573	2	fig|441952.3.peg.237,fig|441952.3.peg.238
Q8ZKP3	fig|99287.1.peg.3940	GlpK	5	fig|99287.1.peg.2207,fig|99287.1.peg.2208,fig|99287.1.peg.3405,fig|99287.1.peg.3576,fig|99287.1.peg.3941	1	fig|99287.1.peg.3941
A3CPV3	fig|388919.8.peg.1620	GlpK	3	fig|388919.8.peg.1622,fig|388919.8.peg.1925,fig|388919.8.peg.488	1	fig|388919.8.peg.1622

A5M8Q4	fig|406560.4.peg.1337	GlpK	2	fig|406560.4.peg.1223,fig|406560.4.peg.1335	1	fig|406560.4.peg.1335
B3IHD4	fig|340186.3.peg.1230	GlpK	5	fig|340186.3.peg.1231,fig|340186.3.peg.1346,fig|340186.3.peg.1347,fig|340186.3.peg.1750,fig|340186.3.peg.2002	1	fig|340186.3.peg.1231
A8GLA1	fig|399741.3.peg.4775	GlpK	5	fig|399741.3.peg.227,fig|399741.3.peg.228,fig|399741.3.peg.4622,fig|399741.3.peg.4774,fig|399741.3.peg.4792	1	fig|399741.3.peg.4774
A4VJY7	fig|379731.4.peg.1612	GlpK	4	fig|379731.4.peg.1610,fig|379731.4.peg.1613,fig|379731.4.peg.1909,fig|379731.4.peg.2854	2	fig|379731.4.peg.1610,fig|379731.4.peg.1613
A9MI40	fig|41514.4.peg.3400	GlpK	5	fig|41514.4.peg.3399,fig|41514.4.peg.3748,fig|41514.4.peg.3903,fig|41514.4.peg.582,fig|41514.4.peg.583	1	fig|41514.4.peg.3399
A6VPR1	fig|339671.5.peg.1576	GlpK	4	fig|339671.5.peg.1575,fig|339671.5.peg.202,fig|339671.5.peg.203,fig|339671.5.peg.381	1	fig|339671.5.peg.1575
P57944	fig|272843.1.peg.1447	GlpK	4	fig|272843.1.peg.1432,fig|272843.1.peg.1442,fig|272843.1.peg.1443,fig|272843.1.peg.1446	3	fig|272843.1.peg.1442,fig|272843.1.peg.1443,fig|272843.1.peg.1446
A5MU26	fig|406563.4.peg.807	GlpK	2	fig|406563.4.peg.1894,fig|406563.4.peg.805	1	fig|406563.4.peg.805
O51257	fig|224326.1.peg.625	GlpK	3	fig|224326.1.peg.624,fig|224326.1.peg.627,fig|224326.1.peg.752	2	fig|224326.1.peg.624,fig|224326.1.peg.627
Q48F01	fig|264730.3.peg.4170	GlpK	3	fig|264730.3.peg.2088,fig|264730.3.peg.4169,fig|264730.3.peg.4172	2	fig|264730.3.peg.4169,fig|264730.3.peg.4172
Q4K734	fig|220664.3.peg.306	GlpK	4	fig|220664.3.peg.2734,fig|220664.3.peg.305,fig|220664.3.peg.308,fig|220664.3.peg.4255	2	fig|220664.3.peg.305,fig|220664.3.peg.308
Q83D14	fig|227377.1.peg.889	GlpK	2	fig|227377.1.peg.1445,fig|227377.1.peg.888	1	fig|227377.1.peg.888
Q8Z2Y6	fig|220341.1.peg.3356	GlpK	5	fig|220341.1.peg.2217,fig|220341.1.peg.2218,fig|220341.1.peg.3355,fig|220341.1.peg.3617,fig|220341.1.peg.3775	1	fig|220341.1.peg.3355
A4J8E6	fig|349161.4.peg.2797	GlpK	4	fig|349161.4.peg.1144,fig|349161.4.peg.2795,fig|349161.4.peg.2796,fig|349161.4.peg.2798	3	fig|349161.4.peg.2795,fig|349161.4.peg.2796,fig|349161.4.peg.2798
Q4ZPI7	fig|205918.4.peg.4340	GlpK	3	fig|205918.4.peg.2499,fig|205918.4.peg.4339,fig|205918.4.peg.4342	2	fig|205918.4.peg.4339,fig|205918.4.peg.4342
C3KBM0	fig|216595.1.peg.7475	GlpK	3	fig|216595.1.peg.2614,fig|216595.1.peg.4828,fig|216595.1.peg.7476	2	fig|216595.1.peg.2614,fig|216595.1.peg.7476
Q1QVQ3	fig|290398.4.peg.2121	GlpK	1	fig|290398.4.peg.2122	1	fig|290398.4.peg.2122
Q0SNS0	fig|390236.5.peg.302	GlpK	3	fig|390236.5.peg.301,fig|390236.5.peg.303,fig|390236.5.peg.427	2	fig|390236.5.peg.301,fig|390236.5.peg.303
Q662C3	fig|290434.1.peg.339	GlpK	3	fig|290434.1.peg.338,fig|290434.1.peg.340,fig|290434.1.peg.461	2	fig|290434.1.peg.338,fig|290434.1.peg.340
A4JHM8	fig|269482.4.peg.5988	GlpK	3	fig|269482.4.peg.5987,fig|269482.4.peg.5989,fig|269482.4.peg.6153	2	fig|269482.4.peg.5987,fig|269482.4.peg.5989
Q1BTT7	fig|331271.3.peg.6140	GlpK	2	fig|331271.3.peg.4083,fig|331271.3.peg.6141	1	fig|331271.3.peg.6141
Q7P1G2	fig|243365.1.peg.251	GlpK	3	fig|243365.1.peg.1129,fig|243365.1.peg.252,fig|243365.1.peg.254	2	fig|243365.1.peg.252,fig|243365.1.peg.254
Q49X93	fig|342451.4.peg.903	GlpK	3	fig|342451.4.peg.1335,fig|342451.4.peg.902,fig|342451.4.peg.904	2	fig|342451.4.peg.902,fig|342451.4.peg.904
A4SPA7	fig|382245.6.peg.2531	GlpK	5	fig|382245.6.peg.2171,fig|382245.6.peg.2172,fig|382245.6.peg.2527,fig|382245.6.peg.2530,fig|382245.6.peg.3843	2	fig|382245.6.peg.2527,fig|382245.6.peg.2530
Q0BC36	fig|339670.3.peg.434	GlpK	3	fig|339670.3.peg.257,fig|339670.3.peg.433,fig|339670.3.peg.435	2	fig|339670.3.peg.433,fig|339670.3.peg.435
B1RR24	fig|195103.9.peg.2755	GlpK	6	fig|195103.9.peg.10,fig|195103.9.peg.1125,fig|195103.9.peg.1951,fig|195103.9.peg.2754,fig|195103.9.peg.2757,fig|195103.9.peg.389	2	fig|195103.9.peg.2754,fig|195103.9.peg.2757
B1BSG4	fig|195102.1.peg.2615	GlpK	5	fig|195102.1.peg.1817,fig|195102.1.peg.2617,fig|195102.1.peg.472,fig|195102.1.peg.75,fig|195102.1.peg.989	1	fig|195102.1.peg.2617
A3SK66	fig|89187.3.peg.1225	GlpK	2	fig|89187.3.peg.1032,fig|89187.3.peg.1224	1	fig|89187.3.peg.1224
Q3K7I5	fig|205922.3.peg.5515	GlpK	3	fig|205922.3.peg.4466,fig|205922.3.peg.5514,fig|205922.3.peg.5517	2	fig|205922.3.peg.5514,fig|205922.3.peg.5517
Q63X50	fig|320390.3.peg.2705	GlpK	3	fig|320390.3.peg.2704,fig|320390.3.peg.2706,fig|320390.3.peg.3441	2	fig|320390.3.peg.2704,fig|320390.3.peg.2706

A6M1Y8	fig|290402.34.peg.4428	GlpK	5	fig|290402.34.peg.10,fig|290402.34.peg.1127,fig|290402.34.peg.2469,fig|290402.34.peg.4427,fig|290402.34.peg.4430	2	fig|290402.34.peg.4427,fig|290402.34.peg.4430
A4CJ47	fig|313596.3.peg.1776	GlpK	3	fig|313596.3.peg.1584,fig|313596.3.peg.1585,fig|313596.3.peg.1775	1	fig|313596.3.peg.1775
Q13UE3	fig|36873.1.peg.5233	GlpK	3	fig|36873.1.peg.5232,fig|36873.1.peg.5234,fig|36873.1.peg.7233	2	fig|36873.1.peg.5232,fig|36873.1.peg.5234
B2FI02	fig|40324.1.peg.3273	GlpK	2	fig|40324.1.peg.1663,fig|40324.1.peg.3272	1	fig|40324.1.peg.3272
A2SM29	fig|420662.8.peg.3445	GlpK	2	fig|420662.8.peg.3444,fig|420662.8.peg.678	1	fig|420662.8.peg.3444
B4SJT3	fig|391008.3.peg.3555	GlpK	2	fig|391008.3.peg.137,fig|391008.3.peg.3554	1	fig|391008.3.peg.3554
A5VZG7	fig|351746.4.peg.1100	GlpK	3	fig|351746.4.peg.1098,fig|351746.4.peg.1101,fig|351746.4.peg.1677	2	fig|351746.4.peg.1098,fig|351746.4.peg.1101
Q8CSS0	fig|176279.3.peg.254	GlpK	3	fig|176279.3.peg.1120,fig|176279.3.peg.253,fig|176279.3.peg.255	2	fig|176279.3.peg.253,fig|176279.3.peg.255
A6LCZ1	fig|435591.10.peg.1729	GlpK	3	fig|435591.10.peg.132,fig|435591.10.peg.1728,fig|435591.10.peg.1730	2	fig|435591.10.peg.1728,fig|435591.10.peg.1730
Q4L607	fig|279808.3.peg.2592	GlpK	3	fig|279808.3.peg.1997,fig|279808.3.peg.2591,fig|279808.3.peg.2596	2	fig|279808.3.peg.2591,fig|279808.3.peg.2596
A1QZ35	fig|314724.3.peg.234	GlpK	3	fig|314724.3.peg.233,fig|314724.3.peg.237,fig|314724.3.peg.357	2	fig|314724.3.peg.233,fig|314724.3.peg.237
A7X1U3	fig|418127.4.peg.1195	GlpK	3	fig|418127.4.peg.1194,fig|418127.4.peg.1196,fig|418127.4.peg.1358	2	fig|418127.4.peg.1194,fig|418127.4.peg.1196
C2K8T7	fig|282458.1.peg.1205	GlpK	3	fig|282458.1.peg.1204,fig|282458.1.peg.1206,fig|282458.1.peg.1403	2	fig|282458.1.peg.1204,fig|282458.1.peg.1206
A3ZQG2	fig|314230.3.peg.1516	GlpK	2	fig|314230.3.peg.1099,fig|314230.3.peg.1517	1	fig|314230.3.peg.1517
A4BZE8	fig|313594.3.peg.1540	GlpK	3	fig|313594.3.peg.1539,fig|313594.3.peg.1541,fig|313594.3.peg.743	2	fig|313594.3.peg.1539,fig|313594.3.peg.1541
Q1IMB2	fig|204669.6.peg.2985	GlpK	4	fig|204669.6.peg.2117,fig|204669.6.peg.2984,fig|204669.6.peg.3318,fig|204669.6.peg.3700	1	fig|204669.6.peg.2984
Q2YXR6	fig|273036.3.peg.1482	GlpK	3	fig|273036.3.peg.1108,fig|273036.3.peg.1481,fig|273036.3.peg.1483	2	fig|273036.3.peg.1481,fig|273036.3.peg.1483
Q97JG4	fig|272562.1.peg.1479	GlpK	5	fig|272562.1.peg.1477,fig|272562.1.peg.1480,fig|272562.1.peg.1867,fig|272562.1.peg.188,fig|272562.1.peg.935	2	fig|272562.1.peg.1477,fig|272562.1.peg.1480
A3I438	fig|101031.3.peg.804	GlpK	4	fig|101031.3.peg.4007,fig|101031.3.peg.68,fig|101031.3.peg.805,fig|101031.3.peg.807	2	fig|101031.3.peg.68,fig|101031.3.peg.805,fig|101031.3.peg.807
C4CLB0	fig|479434.4.peg.869	GlpK	2	fig|479434.4.peg.866,fig|479434.4.peg.880	1	fig|479434.4.peg.866
A3IGA5	fig|101031.3.peg.69	GlpK	4	fig|101031.3.peg.4007,fig|101031.3.peg.68,fig|101031.3.peg.805,fig|101031.3.peg.807	1	fig|101031.3.peg.68
A1VA10	fig|391774.5.peg.375	GlpK	5	fig|391774.5.peg.1269,fig|391774.5.peg.1270,fig|391774.5.peg.355,fig|391774.5.peg.376,fig|391774.5.peg.377	2	fig|391774.5.peg.376,fig|391774.5.peg.377
A3XK62	fig|313593.3.peg.1786	GlpK	3	fig|313593.3.peg.1785,fig|313593.3.peg.1787,fig|313593.3.peg.65	2	fig|313593.3.peg.1785,fig|313593.3.peg.1787
Q8RHZ9	fig|190304.1.peg.318	GlpK	2	fig|190304.1.peg.1482,fig|190304.1.peg.317	1	fig|190304.1.peg.317
A5EWH1	fig|246195.3.peg.210	GlpK	3	fig|246195.3.peg.209,fig|246195.3.peg.211,fig|246195.3.peg.287	2	fig|246195.3.peg.209,fig|246195.3.peg.211
C1Z7E3	fig|485917.5.peg.2941	GlpK	3	fig|485917.5.peg.2942,fig|485917.5.peg.3649,fig|485917.5.peg.3749	1	fig|485917.5.peg.2942
A5IMF5	fig|390874.10.peg.1375	GlpK	2	fig|390874.10.peg.1372,fig|390874.10.peg.529	1	fig|390874.10.peg.1372
B1LBG8	fig|126740.4.peg.1339	GlpK	2	fig|126740.4.peg.1341,fig|126740.4.peg.545	1	fig|126740.4.peg.1341
A4APS0	fig|313603.3.peg.2550	GlpK	3	fig|313603.3.peg.1290,fig|313603.3.peg.2551,fig|313603.3.peg.2553	2	fig|313603.3.peg.2551,fig|313603.3.peg.2553
A3V752	fig|314232.3.peg.2627	GlpK	2	fig|314232.3.peg.204,fig|314232.3.peg.2625	1	fig|314232.3.peg.2625
A4XXN1	fig|399739.6.peg.3233	GlpK	3	fig|399739.6.peg.1519,fig|399739.6.peg.2385,fig|399739.6.peg.3236	1	fig|399739.6.peg.3236
Q3ICL7	fig|326442.4.peg.3476	GlpK	3	fig|326442.4.peg.3472,fig|326442.4.peg.3477,fig|326442.4.peg.354	2	fig|326442.4.peg.3472,fig|326442.4.peg.3477
Q9HY41	fig|208964.1.peg.3579	GlpK	4	fig|208964.1.peg.1615,fig|208964.1.peg.3025,fig|208964.1.peg.3581,fig|208964.1.peg.3584	2	fig|208964.1.peg.3581,fig|208964.1.peg.3584

Q02R51	fig|208963.3.peg.1115	GlpK	4	fig|208963.3.peg.1117,fig|208963.3.peg.1120,fig|208963.3.peg.3220,fig|208963.3.peg.593	2	fig|208963.3.peg.1117,fig|208963.3.peg.1120
A6V1L3	fig|381754.5.peg.1506	GlpK	4	fig|381754.5.peg.1501,fig|381754.5.peg.1504,fig|381754.5.peg.2048,fig|381754.5.peg.3491	2	fig|381754.5.peg.1501,fig|381754.5.peg.1504,fig|381754.5.peg.1501,fig|381754.5.peg.1504
A2TYE0	fig|313598.3.peg.1727	GlpK	3	fig|313598.3.peg.1079,fig|313598.3.peg.1729,fig|313598.3.peg.1734	1	fig|313598.3.peg.1729
B7VAK3	fig|557722.3.peg.1500	GlpK	4	fig|557722.3.peg.1495,fig|557722.3.peg.1498,fig|557722.3.peg.2097,fig|557722.3.peg.3814	2	fig|557722.3.peg.1495,fig|557722.3.peg.1498,fig|557722.3.peg.1495,fig|557722.3.peg.1498
A3VKN6	fig|314271.3.peg.3568	GlpK	3	fig|314271.3.peg.1137,fig|314271.3.peg.1840,fig|314271.3.peg.3566	1	fig|314271.3.peg.3566
A6LKN0	fig|391009.4.peg.651	GlpK	2	fig|391009.4.peg.1832,fig|391009.4.peg.654	1	fig|391009.4.peg.654
Q1LL59	fig|266264.4.peg.2636	GlpK	3	fig|266264.4.peg.2637,fig|266264.4.peg.5822,fig|266264.4.peg.660	1	fig|266264.4.peg.2637
Q73CE0	fig|222523.1.peg.1120	GlpK	3	fig|222523.1.peg.1119,fig|222523.1.peg.1121,fig|222523.1.peg.1625	2	fig|222523.1.peg.1119,fig|222523.1.peg.1121
C4EWK3	fig|525903.4.peg.594	GlpK	3	fig|525903.4.peg.1051,fig|525903.4.peg.590,fig|525903.4.peg.595	2	fig|525903.4.peg.590,fig|525903.4.peg.595
Q1AX49	fig|266117.6.peg.975	GlpK	2	fig|266117.6.peg.977,fig|266117.6.peg.981	1	fig|266117.6.peg.977
C1Q0P5	fig|525919.4.peg.1165	GlpK	4	fig|525919.4.peg.1166,fig|525919.4.peg.471,fig|525919.4.peg.472,fig|525919.4.peg.854	1	fig|525919.4.peg.1166
Q3AB25	fig|246194.3.peg.2378	GlpK	4	fig|246194.3.peg.2376,fig|246194.3.peg.2377,fig|246194.3.peg.2379,fig|246194.3.peg.2536	3	fig|246194.3.peg.2376,fig|246194.3.peg.2377,fig|246194.3.peg.2379
B9DV90	fig|218495.3.peg.1315	GlpK	3	fig|218495.3.peg.1313,fig|218495.3.peg.1551,fig|218495.3.peg.481	1	fig|218495.3.peg.1313
Q0SQ01	fig|289380.14.peg.2479	GlpK	5	fig|289380.14.peg.10,fig|289380.14.peg.1704,fig|289380.14.peg.2478,fig|289380.14.peg.2481,fig|289380.14.peg.977	2	fig|289380.14.peg.2478,fig|289380.14.peg.2481
Q8ENK7	fig|221109.1.peg.2474	GlpK	3	fig|221109.1.peg.1797,fig|221109.1.peg.2470,fig|221109.1.peg.2475	2	fig|221109.1.peg.2470,fig|221109.1.peg.2475
Q3BYR0	fig|316273.3.peg.591	GlpK	3	fig|316273.3.peg.592,fig|316273.3.peg.593,fig|316273.3.peg.684	2	fig|316273.3.peg.592,fig|316273.3.peg.593
Q4UZR8	fig|314565.3.peg.388	GlpK	3	fig|314565.3.peg.389,fig|314565.3.peg.390,fig|314565.3.peg.726	2	fig|314565.3.peg.389,fig|314565.3.peg.390
Q87BZ2	fig|183190.1.peg.1247	GlpK	3	fig|183190.1.peg.1016,fig|183190.1.peg.1245,fig|183190.1.peg.1246	2	fig|183190.1.peg.1245,fig|183190.1.peg.1246
Q3R5A3	fig|155920.1.peg.1144	GlpK	3	fig|155920.1.peg.1145,fig|155920.1.peg.1146,fig|155920.1.peg.1853	2	fig|155920.1.peg.1145,fig|155920.1.peg.1146
Q2FYZ5	fig|367830.3.peg.820	GlpK	3	fig|367830.3.peg.1737,fig|367830.3.peg.819,fig|367830.3.peg.821	2	fig|367830.3.peg.819,fig|367830.3.peg.821
Q6G9R3	fig|282459.1.peg.1227	GlpK	3	fig|282459.1.peg.1226,fig|282459.1.peg.1228,fig|282459.1.peg.1401	2	fig|282459.1.peg.1226,fig|282459.1.peg.1228
Q8NWX7	fig|196620.1.peg.1183	GlpK	3	fig|196620.1.peg.1182,fig|196620.1.peg.1184,fig|196620.1.peg.1363	2	fig|196620.1.peg.1182,fig|196620.1.peg.1184
Q9KDW8	fig|272558.1.peg.1093	GlpK	3	fig|272558.1.peg.1092,fig|272558.1.peg.1095,fig|272558.1.peg.1640	2	fig|272558.1.peg.1092,fig|272558.1.peg.1095
Q81GZ2	fig|405532.4.peg.931	GlpK	3	fig|405532.4.peg.1401,fig|405532.4.peg.930,fig|405532.4.peg.932	2	fig|405532.4.peg.930,fig|405532.4.peg.932
Q39V17	fig|269799.3.peg.1993	GlpK	2	fig|269799.3.peg.1994,fig|269799.3.peg.72	1	fig|269799.3.peg.1994
C2PSC4	fig|315730.5.peg.1465	GlpK	3	fig|315730.5.peg.1464,fig|315730.5.peg.1466,fig|315730.5.peg.1956	2	fig|315730.5.peg.1464,fig|315730.5.peg.1466
Q749I1	fig|243231.1.peg.2744	GlpK	2	fig|243231.1.peg.2743,fig|243231.1.peg.6	1	fig|243231.1.peg.2743
Q81U58	fig|198094.1.peg.955	GlpK	3	fig|198094.1.peg.1405,fig|198094.1.peg.954,fig|198094.1.peg.956	2	fig|198094.1.peg.954,fig|198094.1.peg.956
Q53W24	fig|300852.3.peg.2129	GlpK	2	fig|300852.3.peg.2130,fig|300852.3.peg.943	1	fig|300852.3.peg.2130
Q2GC90	fig|48935.1.peg.3229	GlpK	3	fig|48935.1.peg.1026,fig|48935.1.peg.1580,fig|48935.1.peg.3228	1	fig|48935.1.peg.1580,fig|48935.1.peg.3228
C4EMX7	fig|479432.4.peg.4879	GlpK	6	fig|479432.4.peg.1645,fig|479432.4.peg.1647,fig|479432.4.peg.3547,fig|479432.4.peg.4878,fig|479432.4.peg.5971,fig|479432.4.peg.6609	1	fig|479432.4.peg.1645,fig|479432.4.peg.1647,fig|479432.4.peg.3547,fig|479432.4.peg.4878
Q8NZW9	fig|186103.1.peg.1395	GlpK	3	fig|186103.1.peg.1393,fig|186103.1.peg.165,fig|186103.1.peg.444	1	fig|186103.1.peg.1393
Q1JAF2	fig|370553.3.peg.1404	GlpK	2	fig|370553.3.peg.1402,fig|370553.3.peg.206	1	fig|370553.3.peg.1402
Q1J5E4	fig|370554.3.peg.1492	GlpK	3	fig|370554.3.peg.1490,fig|370554.3.peg.189,fig|370554.3.peg.450	1	fig|370554.3.peg.1490

Q48RX6	fig|319701.3.peg.1805	GlpK	2	fig|319701.3.peg.1803,fig|319701.3.peg.281	1	fig|319701.3.peg.1803
Q8K665	fig|198466.1.peg.1468	GlpK	2	fig|198466.1.peg.1466,fig|198466.1.peg.161	1	fig|198466.1.peg.1466
Q03BB9	fig|321967.8.peg.655	GlpK	4	fig|321967.8.peg.653,fig|321967.8.peg.654,fig|321967.8.peg.793,fig|321967.8.peg.906	2	fig|321967.8.peg.653,fig|321967.8.peg.654
Q02YH8	fig|272622.8.peg.1629	GlpK	6	fig|272622.8.peg.1604,fig|272622.8.peg.1630,fig|272622.8.peg.1631,fig|272622.8.peg.1882,fig|272622.8.peg.2469,fig|272622.8.peg.407	2	fig|272622.8.peg.1630,fig|272622.8.peg.1631
Q88ZF1	fig|220668.1.peg.311	GlpK	5	fig|220668.1.peg.137,fig|220668.1.peg.2790,fig|220668.1.peg.312,fig|220668.1.peg.313,fig|220668.1.peg.641	2	fig|220668.1.peg.312,fig|220668.1.peg.313
Q5XAJ9	fig|286636.1.peg.1429	GlpK	3	fig|286636.1.peg.1427,fig|286636.1.peg.225,fig|286636.1.peg.464	1	fig|286636.1.peg.1427
Q03DS8	fig|278197.10.peg.1429	GlpK	4	fig|278197.10.peg.1090,fig|278197.10.peg.1431,fig|278197.10.peg.1493,fig|278197.10.peg.411	1	fig|278197.10.peg.1431
Q8E794	fig|208435.1.peg.272	GlpK	3	fig|208435.1.peg.1608,fig|208435.1.peg.274,fig|208435.1.peg.405	1	fig|208435.1.peg.274
C4VEE9	fig|226185.1.peg.1795	GlpK	2	fig|226185.1.peg.1635,fig|226185.1.peg.1793	1	fig|226185.1.peg.1793
Q9CG64	fig|272623.1.peg.1281	GlpK	6	fig|272623.1.peg.1279,fig|272623.1.peg.1280,fig|272623.1.peg.1373,fig|272623.1.peg.1665,fig|272623.1.peg.2102,fig|272623.1.peg.259	2	fig|272623.1.peg.1279,fig|272623.1.peg.1280
C5F6I2	fig|321967.8.peg.241	GntK	2	fig|321967.8.peg.1649,fig|321967.8.peg.240	1	fig|321967.8.peg.240
B3W8Q2	fig|543734.3.peg.221	GntK	2	fig|543734.3.peg.1806,fig|543734.3.peg.220	1	fig|543734.3.peg.220
Q03EH2	fig|278197.10.peg.1196	GntK	2	fig|278197.10.peg.1197,fig|278197.10.peg.636	1	fig|278197.10.peg.1197
Q9CDN5	fig|272623.1.peg.2239	GntK	2	fig|272623.1.peg.2240,fig|272623.1.peg.637	1	fig|272623.1.peg.2240
Q4MXU4	fig|269801.1.peg.135	GntK	3	fig|269801.1.peg.1150,fig|269801.1.peg.132,fig|269801.1.peg.1533	1	fig|269801.1.peg.1150,fig|269801.1.peg.132
B0PX16	fig|280477.3.peg.4500	GntK	2	fig|280477.3.peg.179,fig|280477.3.peg.4503	1	fig|280477.3.peg.179,fig|280477.3.peg.179,fig|280477.3.peg.4503
Q638K2	fig|288681.12.peg.3257	GntK	2	fig|288681.12.peg.160,fig|288681.12.peg.3260	1	fig|288681.12.peg.160,fig|288681.12.peg.3260
A0RGG3	fig|281309.3.peg.3350	GntK	2	fig|281309.3.peg.163,fig|281309.3.peg.3353	1	fig|281309.3.peg.163,fig|281309.3.peg.3353
A3IAP5	fig|101031.3.peg.165	GntK	1	fig|101031.3.peg.167	1	fig|101031.3.peg.167
Q734J8	fig|222523.1.peg.3379	GntK	2	fig|222523.1.peg.2287,fig|222523.1.peg.3382	1	fig|222523.1.peg.2287,fig|222523.1.peg.3382
P12011	fig|224308.1.peg.4012	GntK	2	fig|224308.1.peg.2391,fig|224308.1.peg.4014	1	fig|224308.1.peg.4014
Q65CW4	fig|279010.5.peg.3745	GntK	2	fig|279010.5.peg.3747,fig|279010.5.peg.412	1	fig|279010.5.peg.3747
Q81B23	fig|226900.1.peg.3220	GntK	2	fig|226900.1.peg.2102,fig|226900.1.peg.3223	1	fig|226900.1.peg.2102,fig|226900.1.peg.3223
A9VMK7	fig|315730.5.peg.3652	GntK	2	fig|315730.5.peg.2636,fig|315730.5.peg.3654	1	fig|315730.5.peg.2636,fig|315730.5.peg.3654
Q03YQ6	fig|203120.4.peg.541	GntK	2	fig|203120.4.peg.540,fig|203120.4.peg.908	1	fig|203120.4.peg.540
B1GKI9	fig|280355.3.peg.2781	GntK	2	fig|280355.3.peg.2486,fig|280355.3.peg.2779	1	fig|280355.3.peg.2486,fig|280355.3.peg.2779
Q63H40	fig|288681.12.peg.158	GntK	2	fig|288681.12.peg.160,fig|288681.12.peg.3260	1	fig|288681.12.peg.160
Q6HPK8	fig|281309.3.peg.161	GntK	2	fig|281309.3.peg.163,fig|281309.3.peg.3353	1	fig|281309.3.peg.163
C1EU16	fig|412694.5.peg.214	GntK	2	fig|412694.5.peg.216,fig|412694.5.peg.2935	1	fig|412694.5.peg.216
Q4MUX7	fig|269801.1.peg.1152	GntK	3	fig|269801.1.peg.1150,fig|269801.1.peg.132,fig|269801.1.peg.1533	1	fig|269801.1.peg.1150
B0K7L5	fig|340099.4.peg.591	GntK	3	fig|340099.4.peg.2014,fig|340099.4.peg.589,fig|340099.4.peg.957	1	fig|340099.4.peg.589
Q88XF6	fig|220668.1.peg.1044	GntK	2	fig|220668.1.peg.1045,fig|220668.1.peg.1278	1	fig|220668.1.peg.1045
A2RNY7	fig|416870.7.peg.2457	GntK	2	fig|416870.7.peg.2458,fig|416870.7.peg.580	1	fig|416870.7.peg.2458
Q02VU6	fig|272622.8.peg.2621	GntK	2	fig|272622.8.peg.2622,fig|272622.8.peg.784	1	fig|272622.8.peg.2622
Q6I4N1	fig|198094.1.peg.5316	GntK	2	fig|198094.1.peg.157,fig|198094.1.peg.3143	1	fig|198094.1.peg.3143,fig|198094.1.peg.157,fig|198094.1.peg.157
C3LJZ6	fig|261591.3.peg.3922	GntK	2	fig|261591.3.peg.1859,fig|261591.3.peg.3924	1	fig|261591.3.peg.1859,fig|261591.3.peg.3924,fig|261591.3.peg.3924
B1L950	fig|126740.4.peg.495	GntK	2	fig|126740.4.peg.497,fig|126740.4.peg.500	2	fig|126740.4.peg.497,fig|126740.4.peg.500
Q9WYS4	fig|243274.1.peg.437	GntK	2	fig|243274.1.peg.432,fig|243274.1.peg.435	2	fig|243274.1.peg.432,fig|243274.1.peg.435

A8ARG8	fig|290338.6.peg.4218	LyxK	3	fig|290338.6.peg.4214,fig|290338.6.peg.4219,fig|290338.6.peg.4220	3	fig|290338.6.peg.4214,fig|290338.6.peg.4219,fig|290338.6.peg.4220
B5C9Q2	fig|439843.6.peg.3849	LyxK	3	fig|439843.6.peg.3843,fig|439843.6.peg.3850,fig|439843.6.peg.3851	2	fig|439843.6.peg.3850,fig|439843.6.peg.3851
B5FLF1	fig|439851.5.peg.4018	LyxK	3	fig|439851.5.peg.4012,fig|439851.5.peg.4019,fig|439851.5.peg.4020	2	fig|439851.5.peg.4019,fig|439851.5.peg.4020
Q1R512	fig|364106.7.peg.4035	LyxK	3	fig|364106.7.peg.4029,fig|364106.7.peg.4036,fig|364106.7.peg.4037	2	fig|364106.7.peg.4036,fig|364106.7.peg.4037
Q8FCD0	fig|199310.1.peg.4308	LyxK	3	fig|199310.1.peg.4301,fig|199310.1.peg.4309,fig|199310.1.peg.4310	2	fig|199310.1.peg.4309,fig|199310.1.peg.4310
B3H8E2	fig|340184.3.peg.274	LyxK	3	fig|340184.3.peg.272,fig|340184.3.peg.273,fig|340184.3.peg.279	3	fig|340184.3.peg.272,fig|340184.3.peg.273,fig|340184.3.peg.279
C1HSH9	fig|405955.9.peg.3369	LyxK	3	fig|405955.9.peg.3364,fig|405955.9.peg.3370,fig|405955.9.peg.3371	2	fig|405955.9.peg.3370,fig|405955.9.peg.3371
B7L6Z2	fig|340186.3.peg.1713	LyxK	3	fig|340186.3.peg.1708,fig|340186.3.peg.1714,fig|340186.3.peg.1715	3	fig|340186.3.peg.1708,fig|340186.3.peg.1714,fig|340186.3.peg.1715
B3HZJ6	fig|340185.3.peg.14	LyxK	3	fig|340185.3.peg.12,fig|340185.3.peg.13,fig|340185.3.peg.19	3	fig|340185.3.peg.12,fig|340185.3.peg.13,fig|340185.3.peg.19
Q8Z2C9	fig|209261.1.peg.3576	LyxK	3	fig|209261.1.peg.3574,fig|209261.1.peg.3575,fig|209261.1.peg.3581	2	fig|209261.1.peg.3574,fig|209261.1.peg.3575
B4SWM2	fig|423368.6.peg.3987	LyxK	3	fig|423368.6.peg.3981,fig|423368.6.peg.3988,fig|423368.6.peg.3989	2	fig|423368.6.peg.3988,fig|423368.6.peg.3989
B1IZL3	fig|344610.3.peg.15	LyxK	3	fig|344610.3.peg.13,fig|344610.3.peg.14,fig|344610.3.peg.21	2	fig|344610.3.peg.13,fig|344610.3.peg.14
B5Q768	fig|454169.6.peg.3949	LyxK	3	fig|454169.6.peg.3943,fig|454169.6.peg.3950,fig|454169.6.peg.3951	2	fig|454169.6.peg.3950,fig|454169.6.peg.3951
B7NP80	fig|585057.4.peg.4236	LyxK	3	fig|585057.4.peg.4231,fig|585057.4.peg.4237,fig|585057.4.peg.4238	3	fig|585057.4.peg.4231,fig|585057.4.peg.4237,fig|585057.4.peg.4238
Q0SY98	fig|373384.10.peg.4447	LyxK	3	fig|373384.10.peg.4445,fig|373384.10.peg.4446,fig|373384.10.peg.4455	2	fig|373384.10.peg.4445,fig|373384.10.peg.4446
B5EX85	fig|454166.6.peg.3780	LyxK	3	fig|454166.6.peg.3774,fig|454166.6.peg.3781,fig|454166.6.peg.3782	2	fig|454166.6.peg.3781,fig|454166.6.peg.3782
B5R4R1	fig|550537.3.peg.3723	LyxK	3	fig|550537.3.peg.3717,fig|550537.3.peg.3724,fig|550537.3.peg.3725	2	fig|550537.3.peg.3724,fig|550537.3.peg.3725
A7ZTC7	fig|331111.3.peg.1721	LyxK	3	fig|331111.3.peg.1716,fig|331111.3.peg.1722,fig|331111.3.peg.1723	3	fig|331111.3.peg.1716,fig|331111.3.peg.1722,fig|331111.3.peg.1723
Q0TBM0	fig|340197.3.peg.583	LyxK	3	fig|340197.3.peg.581,fig|340197.3.peg.582,fig|340197.3.peg.589	2	fig|340197.3.peg.581,fig|340197.3.peg.582
B3YDJ1	fig|439842.7.peg.3463	LyxK	3	fig|439842.7.peg.3457,fig|439842.7.peg.3464,fig|439842.7.peg.3465	2	fig|439842.7.peg.3464,fig|439842.7.peg.3465
B5C479	fig|99287.1.peg.3550	LyxK	3	fig|99287.1.peg.3544,fig|99287.1.peg.3551,fig|99287.1.peg.3552	2	fig|99287.1.peg.3551,fig|99287.1.peg.3552
B5PRN3	fig|272994.5.peg.3802	LyxK	3	fig|272994.5.peg.3796,fig|272994.5.peg.3803,fig|272994.5.peg.3804	2	fig|272994.5.peg.3803,fig|272994.5.peg.3804
B7ULE2	fig|216593.1.peg.4322	LyxK	3	fig|216593.1.peg.4316,fig|216593.1.peg.4323,fig|216593.1.peg.4324	2	fig|216593.1.peg.4323,fig|216593.1.peg.4324
A3XSZ3	fig|314290.3.peg.1474	LyxK	2	fig|314290.3.peg.1475,fig|314290.3.peg.1477	2	fig|314290.3.peg.1475,fig|314290.3.peg.1477
C4SXT4	fig|349965.3.peg.195	LyxK	3	fig|349965.3.peg.193,fig|349965.3.peg.194,fig|349965.3.peg.196	3	fig|349965.3.peg.193,fig|349965.3.peg.194,fig|349965.3.peg.196
B5BHV9	fig|554290.7.peg.3721	LyxK	3	fig|554290.7.peg.3715,fig|554290.7.peg.3722,fig|554290.7.peg.3723	2	fig|554290.7.peg.3722,fig|554290.7.peg.3723
P44991	fig|71421.1.peg.989	LyxK	3	fig|71421.1.peg.986,fig|71421.1.peg.988,fig|71421.1.peg.993	3	fig|71421.1.peg.986,fig|71421.1.peg.988,fig|71421.1.peg.993
Q65WK5	fig|221988.1.peg.46	LyxK	3	fig|221988.1.peg.45,fig|221988.1.peg.52,fig|221988.1.peg.54	1	fig|221988.1.peg.45
A5UIF7	fig|374931.9.peg.1736	LyxK	2	fig|374931.9.peg.1735,fig|374931.9.peg.1740	2	fig|374931.9.peg.1735,fig|374931.9.peg.1740
Q0I2L5	fig|205914.5.peg.801	LyxK	3	fig|205914.5.peg.790,fig|205914.5.peg.802,fig|205914.5.peg.803	2	fig|205914.5.peg.802,fig|205914.5.peg.803
B0UTW2	fig|228400.4.peg.1295	LyxK	3	fig|228400.4.peg.1283,fig|228400.4.peg.1296,fig|228400.4.peg.1297	2	fig|228400.4.peg.1296,fig|228400.4.peg.1297
P57928	fig|272843.1.peg.1247	LyxK	3	fig|272843.1.peg.1245,fig|272843.1.peg.1246,fig|272843.1.peg.1256	2	fig|272843.1.peg.1245,fig|272843.1.peg.1246

A5EAI3	fig|288000.5.peg.1100	RbtK	2	fig|288000.5.peg.1068,fig|288000.5.peg.1099	1	fig|288000.5.peg.1099
Q89QA4	fig|224911.1.peg.3226	RbtK	2	fig|224911.1.peg.3225,fig|224911.1.peg.6662	1	fig|224911.1.peg.3225
Q1M8S5	fig|216596.1.peg.7085	RbtK	5	fig|216596.1.peg.4352,fig|216596.1.peg.686,fig|216596.1.peg.6957,fig|216596.1.peg.6958,fig|216596.1.peg.7084	1	fig|216596.1.peg.4352,fig|216596.1.peg.7084
Q3IVW9	fig|272943.3.peg.3604	RbtK	3	fig|272943.3.peg.2032,fig|272943.3.peg.2033,fig|272943.3.peg.3603	1	fig|272943.3.peg.3603
C4IWD3	fig|224914.1.peg.3039	RbtK	1	fig|224914.1.peg.3040	1	fig|224914.1.peg.3040
Q8FX21	fig|204722.1.peg.2375	RbtK	1	fig|204722.1.peg.2374	1	fig|204722.1.peg.2374
Q6DA23	fig|218491.3.peg.4084	RhaB	2	fig|218491.3.peg.4082,fig|218491.3.peg.4083	2	fig|218491.3.peg.4082,fig|218491.3.peg.4083
Q83IU3	fig|198214.1.peg.3748	RhaB	2	fig|198214.1.peg.3746,fig|198214.1.peg.3747	2	fig|198214.1.peg.3746,fig|198214.1.peg.3747
B5NTQ5	fig|439842.7.peg.3839	RhaB	2	fig|439842.7.peg.3837,fig|439842.7.peg.3838	2	fig|439842.7.peg.3837,fig|439842.7.peg.3838
B3I5Y6	fig|344601.3.peg.1720	RhaB	2	fig|344601.3.peg.1721,fig|344601.3.peg.1722	2	fig|344601.3.peg.1721,fig|344601.3.peg.1722
B3BFS9	fig|83334.1.peg.4805	RhaB	2	fig|83334.1.peg.4803,fig|83334.1.peg.4804	2	fig|83334.1.peg.4803,fig|83334.1.peg.4804
B7MI36	fig|364106.7.peg.4374	RhaB	2	fig|364106.7.peg.4372,fig|364106.7.peg.4373	2	fig|364106.7.peg.4372,fig|364106.7.peg.4373
B3IHB1	fig|340186.3.peg.1207	RhaB	2	fig|340186.3.peg.1205,fig|340186.3.peg.1206	2	fig|340186.3.peg.1205,fig|340186.3.peg.1206
B5MMZ8	fig|550537.3.peg.4090	RhaB	2	fig|550537.3.peg.4088,fig|550537.3.peg.4089	2	fig|550537.3.peg.4088,fig|550537.3.peg.4089
B5P7I0	fig|454169.6.peg.4312	RhaB	2	fig|454169.6.peg.4310,fig|454169.6.peg.4311	2	fig|454169.6.peg.4310,fig|454169.6.peg.4311
B3HBT3	fig|340184.3.peg.1501	RhaB	2	fig|340184.3.peg.1499,fig|340184.3.peg.1500	2	fig|340184.3.peg.1499,fig|340184.3.peg.1500
B5FPP4	fig|439851.5.peg.4384	RhaB	2	fig|439851.5.peg.4382,fig|439851.5.peg.4383	2	fig|439851.5.peg.4382,fig|439851.5.peg.4383
B7UNM5	fig|216593.1.peg.4734	RhaB	2	fig|216593.1.peg.4732,fig|216593.1.peg.4733	2	fig|216593.1.peg.4732,fig|216593.1.peg.4733
B1XB71	fig|83333.1.peg.3825	RhaB	2	fig|83333.1.peg.3823,fig|83333.1.peg.3824	2	fig|83333.1.peg.3823,fig|83333.1.peg.3824
C0Q3L3	fig|321314.4.peg.3436	RhaB	2	fig|321314.4.peg.3434,fig|321314.4.peg.3435	2	fig|321314.4.peg.3434,fig|321314.4.peg.3435
B7L9G0	fig|585034.4.peg.4001	RhaB	2	fig|585034.4.peg.3999,fig|585034.4.peg.4000	2	fig|585034.4.peg.3999,fig|585034.4.peg.4000
B5F0M8	fig|454166.6.peg.4164	RhaB	2	fig|454166.6.peg.4162,fig|454166.6.peg.4163	2	fig|454166.6.peg.4162,fig|454166.6.peg.4163
B5C7K8	fig|99287.1.peg.3901	RhaB	2	fig|99287.1.peg.3899,fig|99287.1.peg.3900	2	fig|99287.1.peg.3899,fig|99287.1.peg.3900
B3HTS7	fig|362663.8.peg.4143	RhaB	2	fig|362663.8.peg.4141,fig|362663.8.peg.4142	2	fig|362663.8.peg.4141,fig|362663.8.peg.4142
B4TPQ8	fig|439843.6.peg.4214	RhaB	2	fig|439843.6.peg.4212,fig|439843.6.peg.4213	2	fig|439843.6.peg.4212,fig|439843.6.peg.4213
B5BJG7	fig|554290.7.peg.4099	RhaB	2	fig|554290.7.peg.4097,fig|554290.7.peg.4098	2	fig|554290.7.peg.4097,fig|554290.7.peg.4098
B7NUA2	fig|585057.4.peg.3200	RhaB	2	fig|585057.4.peg.3201,fig|585057.4.peg.3204	2	fig|585057.4.peg.3201,fig|585057.4.peg.3204
Q8Z2V4	fig|220341.1.peg.3393	RhaB	2	fig|220341.1.peg.3394,fig|220341.1.peg.4759	2	fig|220341.1.peg.3394,fig|220341.1.peg.4759
A8A707	fig|331112.3.peg.3858	RhaB	2	fig|331112.3.peg.3856,fig|331112.3.peg.3857	2	fig|331112.3.peg.3856,fig|331112.3.peg.3857
Q3YV72	fig|300269.11.peg.4690	RhaB	2	fig|300269.11.peg.4688,fig|300269.11.peg.4689	2	fig|300269.11.peg.4688,fig|300269.11.peg.4689
A9MZC6	fig|272994.5.peg.4165	RhaB	2	fig|272994.5.peg.4163,fig|272994.5.peg.4164	2	fig|272994.5.peg.4163,fig|272994.5.peg.4164
Q1CEB3	fig|187410.1.peg.576	RhaB	2	fig|187410.1.peg.574,fig|187410.1.peg.575	2	fig|187410.1.peg.574,fig|187410.1.peg.575
A9QYS1	fig|349746.3.peg.2147	RhaB	2	fig|349746.3.peg.2145,fig|349746.3.peg.2146	2	fig|349746.3.peg.2145,fig|349746.3.peg.2146
C4T6J3	fig|349965.3.peg.3107	RhaB	2	fig|349965.3.peg.3108,fig|349965.3.peg.3109	2	fig|349965.3.peg.3108,fig|349965.3.peg.3109
Q65Q24	fig|221988.1.peg.2189	RhaB	2	fig|221988.1.peg.2187,fig|221988.1.peg.2188	2	fig|221988.1.peg.2187,fig|221988.1.peg.2188
B1JNC8	fig|502800.3.peg.3796	RhaB	2	fig|502800.3.peg.3797,fig|502800.3.peg.3798	2	fig|502800.3.peg.3797,fig|502800.3.peg.3798
A8AL29	fig|290338.6.peg.2603	RhaB	2	fig|290338.6.peg.2604,fig|290338.6.peg.2605	2	fig|290338.6.peg.2604,fig|290338.6.peg.2605
Q31U85	fig|300268.10.peg.4659	RhaB	2	fig|300268.10.peg.4657,fig|300268.10.peg.4658	2	fig|300268.10.peg.4657,fig|300268.10.peg.4658
Q32A69	fig|216598.1.peg.3988	RhaB	2	fig|216598.1.peg.3989,fig|216598.1.peg.3990	2	fig|216598.1.peg.3989,fig|216598.1.peg.3990
Q1AVD2	fig|266117.6.peg.1540	RhaB	2	fig|266117.6.peg.1534,fig|266117.6.peg.1535	1	fig|266117.6.peg.1535
C0V0B3	fig|525904.4.peg.207	RhaB	3	fig|525904.4.peg.208,fig|525904.4.peg.209,fig|525904.4.peg.210	3	fig|525904.4.peg.208,fig|525904.4.peg.209,fig|525904.4.peg.210
Q9X0G2	fig|243274.1.peg.1063	RhaB	2	fig|243274.1.peg.1061,fig|243274.1.peg.1062	2	fig|243274.1.peg.1061,fig|243274.1.peg.1062

A5INA3	fig|390874.10.peg.1685	RhaB	2	fig|390874.10.peg.1686,fig|390874.10.peg.1687	2	fig|390874.10.peg.1686,fig|390874.10.peg.1687
Q9WXX1	fig|243274.1.peg.116	XylB	1	fig|243274.1.peg.1651	0	-
Q83EH5	fig|227377.1.peg.331	XylB	2	fig|227377.1.peg.2064,fig|227377.1.peg.332	2	fig|227377.1.peg.2064,fig|227377.1.peg.332
Q15PG1	fig|342610.3.peg.3714	XylB	3	fig|342610.3.peg.1752,fig|342610.3.peg.3715,fig|342610.3.peg.3716	2	fig|342610.3.peg.3715,fig|342610.3.peg.3716
Q1BG91	fig|331271.3.peg.1043	XylB	3	fig|331271.3.peg.1042,fig|331271.3.peg.1044,fig|331271.3.peg.5740	2	fig|331271.3.peg.1042,fig|331271.3.peg.1044
B6J5X8	fig|434924.4.peg.559	XylB	1	fig|434924.4.peg.558	1	fig|434924.4.peg.558
Q0I345	fig|205914.5.peg.613	XylB	2	fig|205914.5.peg.611,fig|205914.5.peg.612	2	fig|205914.5.peg.611,fig|205914.5.peg.612
Q6DB06	fig|218491.3.peg.3058	XylB	2	fig|218491.3.peg.3059,fig|218491.3.peg.3060	2	fig|218491.3.peg.3059,fig|218491.3.peg.3060
Q5PLM7	fig|554290.7.peg.3706	XylB	1	fig|554290.7.peg.3707	1	fig|554290.7.peg.3707
B7L6X7	fig|340185.3.peg.30	XylB	3	fig|340185.3.peg.1317,fig|340185.3.peg.28,fig|340185.3.peg.29	2	fig|340185.3.peg.28,fig|340185.3.peg.29
Q0SY84	fig|198215.1.peg.3578	XylB	3	fig|198215.1.peg.3051,fig|198215.1.peg.3577,fig|198215.1.peg.4072	2	fig|198215.1.peg.3577,fig|198215.1.peg.4072
C4T286	fig|349965.3.peg.2781	XylB	2	fig|349965.3.peg.2782,fig|349965.3.peg.2783	2	fig|349965.3.peg.2782,fig|349965.3.peg.2783
C4W1M0	fig|331112.3.peg.3527	XylB	3	fig|331112.3.peg.3528,fig|331112.3.peg.3529,fig|331112.3.peg.3983	2	fig|331112.3.peg.3528,fig|331112.3.peg.3529
B7NP64	fig|585057.4.peg.4219	XylB	2	fig|585057.4.peg.4220,fig|585057.4.peg.4221	2	fig|585057.4.peg.4220,fig|585057.4.peg.4221
Q8Z2B7	fig|220341.1.peg.3657	XylB	1	fig|220341.1.peg.3656	1	fig|220341.1.peg.3656
C4SC56	fig|349967.3.peg.716	XylB	2	fig|349967.3.peg.717,fig|349967.3.peg.718	2	fig|349967.3.peg.717,fig|349967.3.peg.718
Q663Y2	fig|273123.1.peg.3950	XylB	2	fig|273123.1.peg.3948,fig|273123.1.peg.3949	2	fig|273123.1.peg.3948,fig|273123.1.peg.3949
Q8CZH7	fig|187410.1.peg.4002	XylB	2	fig|187410.1.peg.4000,fig|187410.1.peg.4001	2	fig|187410.1.peg.4000,fig|187410.1.peg.4001
B7ULC5	fig|216593.1.peg.4305	XylB	2	fig|216593.1.peg.4306,fig|216593.1.peg.4307	2	fig|216593.1.peg.4306,fig|216593.1.peg.4307
C4S052	fig|349968.3.peg.182	XylB	2	fig|349968.3.peg.180,fig|349968.3.peg.181	2	fig|349968.3.peg.180,fig|349968.3.peg.181
C4X272	fig|272620.3.peg.3969	XylB	3	fig|272620.3.peg.3970,fig|272620.3.peg.3971,fig|272620.3.peg.4736	2	fig|272620.3.peg.3970,fig|272620.3.peg.3971
Q8FCE4	fig|199310.1.peg.4289	XylB	2	fig|199310.1.peg.4290,fig|199310.1.peg.4291	2	fig|199310.1.peg.4290,fig|199310.1.peg.4291
C4SM10	fig|349966.3.peg.2473	XylB	2	fig|349966.3.peg.2471,fig|349966.3.peg.2472	2	fig|349966.3.peg.2471,fig|349966.3.peg.2472
Q6LUX7	fig|298386.1.peg.2696	XylB	2	fig|298386.1.peg.2686,fig|298386.1.peg.2691	1	fig|298386.1.peg.2691
Q65PY1	fig|221988.1.peg.2230	XylB	2	fig|221988.1.peg.2231,fig|221988.1.peg.2232	2	fig|221988.1.peg.2231,fig|221988.1.peg.2232
Q2GAC0	fig|48935.1.peg.1771	XylB	2	fig|48935.1.peg.1772,fig|48935.1.peg.1867	1	fig|48935.1.peg.1772
Q3YVU9	fig|300269.11.peg.4392	XylB	2	fig|300269.11.peg.4391,fig|300269.11.peg.4836	1	fig|300269.11.peg.4391
Q31V54	fig|300268.10.peg.4268	XylB	3	fig|300268.10.peg.4269,fig|300268.10.peg.4270,fig|300268.10.peg.4803	2	fig|300268.10.peg.4269,fig|300268.10.peg.4270,fig|300268.10.peg.4269,fig|300268.10.peg.4270
Q03TX2	fig|387344.13.peg.182	XylB	3	fig|387344.13.peg.181,fig|387344.13.peg.184,fig|387344.13.peg.2090	2	fig|387344.13.peg.181,fig|387344.13.peg.184
Q739D1	fig|222523.1.peg.2195	XylB	2	fig|222523.1.peg.2194,fig|222523.1.peg.2198	2	fig|222523.1.peg.2194,fig|222523.1.peg.2198
P39211	fig|224308.1.peg.1765	XylB	1	fig|224308.1.peg.1764	1	fig|224308.1.peg.1764
Q5WKJ2	fig|66692.3.peg.574	XylB	1	fig|66692.3.peg.573	1	fig|66692.3.peg.573
Q8CX70	fig|221109.1.peg.3121	XylB	1	fig|221109.1.peg.3122	1	fig|221109.1.peg.3122
Q65DK4	fig|279010.5.peg.3168	XylB	1	fig|279010.5.peg.3169	1	fig|279010.5.peg.3169
Q9K994	fig|272558.1.peg.2756	XylB	1	fig|272558.1.peg.2757	1	fig|272558.1.peg.2757
C1UX26	fig|502025.5.peg.2240	XylB	2	fig|502025.5.peg.2239,fig|502025.5.peg.4505	1	fig|502025.5.peg.2239
B0UT20	fig|228400.4.peg.987	XylB	2	fig|228400.4.peg.985,fig|228400.4.peg.986	2	fig|228400.4.peg.985,fig|228400.4.peg.986
B0GU21	fig|349746.3.peg.3071	XylB	3	fig|349746.3.peg.3072,fig|349746.3.peg.3073,fig|349746.3.peg.3074	3	fig|349746.3.peg.3072,fig|349746.3.peg.3073,fig|349746.3.peg.3074
B1JH39	fig|502800.3.peg.55	XylB	2	fig|502800.3.peg.56,fig|502800.3.peg.57	2	fig|502800.3.peg.56,fig|502800.3.peg.57
A7ZTB1	fig|331111.3.peg.1705	XylB	3	fig|331111.3.peg.1706,fig|331111.3.peg.1707,fig|331111.3.peg.2182	2	fig|331111.3.peg.1706,fig|331111.3.peg.1707
B3AWW2	fig|83334.1.peg.4418	XylB	3	fig|83334.1.peg.4419,fig|83334.1.peg.4420,fig|83334.1.peg.4990	2	fig|83334.1.peg.4419,fig|83334.1.peg.4420
A4W567	fig|399742.4.peg.239	XylB	2	fig|399742.4.peg.237,fig|399742.4.peg.238	2	fig|399742.4.peg.237,fig|399742.4.peg.238
B1IZM8	fig|481805.3.peg.158	XylB	3	fig|481805.3.peg.156,fig|481805.3.peg.157,fig|481805.3.peg.4299	2	fig|481805.3.peg.156,fig|481805.3.peg.157

B1X8I0	fig|83333.1.peg.3498	XylB	3	fig|83333.1.peg.3499,fig|83333.1.peg.3500,fig|83333.1.peg.3940	2	fig|83333.1.peg.3499,fig|83333.1.peg.3500
B4F2W7	fig|584.1.peg.2287	XylB	1	fig|584.1.peg.2286	1	fig|584.1.peg.2286
A7FP69	fig|349747.3.peg.2621	XylB	2	fig|349747.3.peg.2622,fig|349747.3.peg.2623	2	fig|349747.3.peg.2622,fig|349747.3.peg.2623
A8G7W7	fig|399741.3.peg.128	XylB	2	fig|399741.3.peg.129,fig|399741.3.peg.130	2	fig|399741.3.peg.129,fig|399741.3.peg.130
A6VWG3	fig|400668.6.peg.1903	XylB	3	fig|400668.6.peg.1900,fig|400668.6.peg.1912,fig|400668.6.peg.2022	1	fig|400668.6.peg.1900
Q93HF4	fig|227882.1.peg.7183	XylB	1	fig|227882.1.peg.7184	1	fig|227882.1.peg.7184
A8LDF9	fig|298653.4.peg.4564	XylB	1	fig|298653.4.peg.4563	1	fig|298653.4.peg.4563
A4XAS3	fig|369723.3.peg.3537	XylB	2	fig|369723.3.peg.3538,fig|369723.3.peg.3791	1	fig|369723.3.peg.3538
C1W0X2	fig|471856.4.peg.1777	XylB	1	fig|471856.4.peg.1778	1	fig|471856.4.peg.1778
Q9RK00	fig|100226.1.peg.1137	XylB	1	fig|100226.1.peg.1136	1	fig|100226.1.peg.1136
B2HNZ2	fig|216594.1.peg.3495	XylB	1	fig|216594.1.peg.3496	1	fig|216594.1.peg.3496
C1RPJ6	fig|446466.5.peg.841	XylB	1	fig|446466.5.peg.840	1	fig|446466.5.peg.840
A8M2F3	fig|391037.3.peg.3826	XylB	2	fig|391037.3.peg.3827,fig|391037.3.peg.4081	1	fig|391037.3.peg.3827
C5C1D9	fig|471853.4.peg.3727	XylB	1	fig|471853.4.peg.3728	1	fig|471853.4.peg.3728
A0LWM7	fig|351607.5.peg.2061	XylB	2	fig|351607.5.peg.1128,fig|351607.5.peg.2060	1	fig|351607.5.peg.2060
Q6AGZ2	fig|281090.3.peg.1380	XylB	1	fig|281090.3.peg.1381	1	fig|281090.3.peg.1381
C1WJY8	fig|479435.4.peg.3778	XylB	4	fig|479435.4.peg.3776,fig|479435.4.peg.513,fig|479435.4.peg.6655,fig|479435.4.peg.6702	1	fig|479435.4.peg.3776
A4AK18	fig|312284.3.peg.1739	XylB	1	fig|312284.3.peg.1740	1	fig|312284.3.peg.1740
C4DF67	fig|446470.4.peg.2217	XylB	2	fig|446470.4.peg.2216,fig|446470.4.peg.4321	1	fig|446470.4.peg.2216
Q47PI0	fig|269800.4.peg.1887	XylB	1	fig|269800.4.peg.1886	1	fig|269800.4.peg.1886
A1TFW7	fig|350058.5.peg.5216	XylB	2	fig|350058.5.peg.5213,fig|350058.5.peg.5214	2	fig|350058.5.peg.5213,fig|350058.5.peg.5214
C4BF18	fig|446465.4.peg.1136	XylB	1	fig|446465.4.peg.1135	1	fig|446465.4.peg.1135
C0T3B0	fig|446462.5.peg.5819	XylB	3	fig|446462.5.peg.273,fig|446462.5.peg.5820,fig|446462.5.peg.6514	1	fig|446462.5.peg.5820
C1QVV7	fig|479433.4.peg.6044	XylB	3	fig|479433.4.peg.1979,fig|479433.4.peg.6043,fig|479433.4.peg.7854	1	fig|479433.4.peg.6043
Q0SCW9	fig|101510.15.peg.2837	XylB	2	fig|101510.15.peg.251,fig|101510.15.peg.2835	1	fig|101510.15.peg.2835
Q6M8P0	fig|196627.4.peg.150	XylB	1	fig|196627.4.peg.145	1	fig|196627.4.peg.145
