# Supplementary material for: The FGGY Carbohydrate Kinase Family: Insights into the Evolution of Functional Specificities
Source: PLoS Comput Biol. 2011 Dec 22;7(12):e1002318. doi: 10.1371/journal.pcbi.1002318 (PMC3245297; doi:10.1371/journal.pcbi.1002318)
Supplement: Table S3 — Prediction of FGGY kinase functions in a list of selected genomes based on genomic and functional context prediction, as well as the identity of signature residues. Taxonomy_ID: the taxonomy identification numbers of the species; Species (Strain): the name and strain number of the species; Uniprot_list: the list of FGGY proteins identified by their Uniprot Accession numbers; Taxonomy_level: the taxonomy levels chosen in Figure 5 to collapse the species tree; Species: the name of species without counting strain names (when calculating the existence of various FGGY functions in the species tree, the counts were averaged among various strains of the same species, which is represented only once on the tree); Functions: the existence of various FGGY functions (see Table 1 for the full names of different functions), a number larger than 0 indicates a function exists in the genome, “0” indicates a function does not exist in the genome. (PDF) [file pcbi.1002318.s006.pdf]

Table S3. **Prediction of FGGY kinase functions in a list of selected genomes based on genomic and functional context prediction, as well as the identity of signature residues.** Taxonomy_ID: the taxonomy identification numbers of the species; Species (Strain): the name and strain number of the species; Uniprot_list: the list of FGGY proteins identified by their Uniprot Accession numbers; Taxonomy_level: the taxonomy levels chosen in Figure 5 to collapse the species tree; Species: the name of species without counting strain names (when calculation existance of various FGGY functions in the species tree, the counts were averaged among various strains of the same species, which is represented only once on the tree); Functions: the existance of various FGGY functions (see Table 1 for the full names of different functions), a number larger than zero indicates a function exist in the genome, "0" indicates a function not exist in the genome.

Taxonomy_ID	Species (Strain)	Uniprot_list	Taxonomy_level	Species	GlpK	AraB	RbtK	GntK	XylB	LyxK	EryA	FucK	RhaB
274	Thermus thermophilus.	O66131	Thermales	Thermus thermophilus	1	0	0	0	0	0	0	0	0
300852	Thermus thermophilus (strain HB8 / ATCC 27634 / DSM 579).	Q53W24	Thermales	Thermus thermophilus	1	0	0	0	0	0	0	0	0
390874	Thermotoga petrophila (strain RKU-1 / ATCC BAA-488 / DSM 13995).	A5IKC5,A5IMF5,A5INA3,A5IJX8,A5IKV4,A5IKB0	Thermotogales	Thermotoga petrophila	1	1	0	1	1	0	0	0	1
126740	Thermotoga sp. (strain RQ2).	B1L950,B1L847,B1LBG8,B1LA35,B1L9K8	Thermotogales	Thermotoga sp RQ2	1	1	0	1	1	0	0	0	1
2336	Thermotoga maritima.	Q9WXX1,Q9X0G2,Q9WYS4,Q9X049,Q9WYC0,Q9X1E4	Thermotogales	Thermotoga maritima	1	1	0	1	1	0	0	0	1
391009	Thermosipho melanesiensis (strain BI429 / DSM 12029).	A6LLR9,A6LKN0	Thermotogales	Thermosipho melanesiensis	1	0	0	0	0	0	0	0	0
266117	Rubrobacter xylanophilus (strain DSM 9941 / NBRC 16129).	Q1AS19,Q1AXS7,Q1AX49,Q1AZ01,Q1AVE2,Q1AVD2	Rubrobacteridae	Rubrobacter xylanophilus	1	0	0	0	1	0	0	0	1
205913	Bifidobacterium longum (strain DJO10A).	B3DTJ7,B3DR48,B3DQ97	Actinobacteridae	Bifidobacterium longum	0	1	0	0	1	0	0	0	0
1718	Corynebacterium glutamicum (Brevibacterium flavum).	Q8NU35,Q8NLP9,Q8NLD4,Q6M8P0,C4B4W2	Actinobacteridae	Corynebacterium glutamicum	1	0	0	0	2	0	0	0	0
1902	Streptomyces coelicolor.	Q9RJM2,Q8CJZ5,Q9XAB1,Q9ADA7,Q9KZE9,Q9RK00	Actinobacteridae	Streptomyces coelicolor	2	0	0	0	1	0	0	0	1
33903	Streptomyces avermitilis.	Q82P07,Q79Z15,Q826J2,Q82BM1,Q93HF4,Q827G1,Q825P4,Q828K5	Actinobacteridae	Streptomyces avermitilis	3	0	0	0	2	0	0	0	1
369723	Salinispora tropica (strain ATCC BAA-916 / DSM 44818 / CNB-440).	A4X282,A4XAS3,A4X7Q9,A4X279,A4X940	Actinobacteridae	Salinispora tropica	3	0	0	0	1	0	0	0	0
391037	Salinispora arenicola (strain CNS-205).	A8M7K2,A8LUZ6,A8M7J9,A8M2F3	Actinobacteridae	Salinispora arenicola	2	0	0	0	1	0	0	0	0
101510	Rhodococcus sp. (strain RHA1).	Q0S996,Q0SCN0,Q0S345,Q0SCW9	Actinobacteridae	Rhodococcus sp RHA1	1	0	0	0	0	0	0	0	1
216594	Mycobacterium marinum (strain ATCC BAA-535 / M).	B2HNZ2,B2HCV0,B2HKB6	Actinobacteridae	Mycobacterium marinum	1	0	0	0	0	0	0	0	0
350058	Mycobacterium vanbaalenii (strain DSM 7251 / PYR-1).	A1TGD7,A1TC06,A1T8D3,A1TFW7	Actinobacteridae	Mycobacterium vanbaalenii	1	0	0	0	0	0	0	0	0
298653	Frankia sp. (strain EAN1pec).	A8LFR7,A8LEH6,A8LDF9,A8LGD5,A8KYT3	Actinobacteridae	Frankia sp EAN1pec	1	0	0	1	1	0	0	0	1
351607	Acidothermus cellulolyticus (strain ATCC 43068 / 11B).	A0LWM7,A0LST5,A0LT84	Actinobacteridae	Acidothermus cellulolyticus	0	1	0	0	1	0	0	0	0
269800	Thermobifida fusca (strain YX).	Q47RU2,Q47PK8,Q47PI0	Actinobacteridae	Thermobifida fusca	1	0	0	0	1	0	0	0	0
59736	Leifsonia xyli subsp. xyli.	Q6AGZ2,Q6AGR0	Actinobacteridae	Leifsonia xyli	1	0	0	0	1	0	0	0	0
471853	Beutenbergia cavernae (strain ATCC BAA-8 / DSM 12333 / NBRC 16432).	C5C324,C5C1D9,C5C1A7,C5C367,C5C1C4	Actinobacteridae	Beutenbergia cavernae	1	1	0	0	1	0	0	0	1
399726	Thermoanaerobacter sp. (strain X514).	B0K643,B0K2Y5,B0K1L4,B0K6A6	Thermoanaerobacterales_1	Thermoanaerobacter sp X514	1	0	0	1	1	0	0	0	0
340099	Thermoanaerobacter pseudethanolicus (strain ATCC 33223 / 39E)(Clostridium thermohydrosulfuricum).	B0K7B7,B0K7L5,B0K754	Thermoanaerobacterales_1	Thermoanaerobacter pseudethanolicus	1	0	0	1	1	0	0	0	0
290402	Clostridium beijerinckii (strain ATCC 51743 / NCIMB 8052) (Clostridiumacetobutylicum).	A6M1Y8,A6LQK3,A6LW14,A6M1T1	Clostridiales_1	Clostridium beijerinckii	1	1	0	0	1	0	0	0	1
289380	Clostridium perfringens (strain SM101 / Type A).	Q0SQ01	Clostridiales_1	Clostridium perfringens	1	0	0	0	0	0	0	0	0
1502	Clostridium perfringens.	Q8XNL6,Q8XHD3	Clostridiales_1	Clostridium perfringens	1	0	0	0	0	0	0	1	0
195103	Clostridium perfringens (strain ATCC 13124 / NCTC 8237 / Type A).	Q0TS95,Q0TMA0	Clostridiales_1	Clostridium perfringens	1	0	0	0	0	0	0	0	0
1488	Clostridium acetobutylicum.	Q97JE2,Q97FW4,Q97JG4	Clostridiales_1	Clostridium acetobutylicum	1	1	0	0	0	0	0	0	0
262543	Exiguobacterium sibiricum (strain DSM 17290 / JCM 13490 / 255-15).	B1YE68,B1YKL3,B1YG58	Bacillales_1	Exiguobacterium sibiricum	2	0	0	1	0	0	0	0	0
86665	Bacillus halodurans.	Q9K9H1,Q9KBQ3,Q9K994,Q9KCM0,Q9KDW8	Bacillales_1	Bacillus halodurans	1	1	0	1	1	0	0	0	1

66692	Bacillus clausii (strain KSM-K16).	Q5WIL1,Q5WKJ2,Q5WGH0,Q5WL39,Q5WL06,Q5WCJ3	Bacillales_1	Bacillus clausii	2	1	0	1	1	0	0	0	0
182710	Oceanobacillus iheyensis.	Q8CX70,Q8ESX1,Q8ELN3,Q8EMP2,Q8ENK7	Bacillales_1	Oceanobacillus iheyensis	1	1	0	1	1	0	0	0	1
203123	Oenococcus oeni (strain BAA-331 / PSU-1).	Q04E40,Q04H58	Lactobacillales	Oenococcus oeni	0	1	0	1	0	0	0	0	0
203120	Leuconostoc mesenteroides subsp. mesenteroides (strain ATCC 8293 /NCDO 523).	Q03YQ6,Q03XW0,Q03ZU0	Lactobacillales	Leuconostoc mesenteroides	0	1	0	1	1	0	0	0	0
334390	Lactobacillus fermentum (strain IFO 3956 / LMG 18251).	B2GAQ7,B2GE12,B2GCS5,B2GCK1	Lactobacillales	Lactobacillus fermentum	1	1	0	0	1	0	0	0	0
278197	Pediococcus pentosaceus (strain ATCC 25745 / 183-1w).	Q03HQ2,Q03DS8,Q03HN0,Q03EH2	Lactobacillales	Pediococcus pentosaceus	1	1	0	1	1	0	0	0	0
1590	Lactobacillus plantarum.	Q88XF6,Q88YD9,Q88ZF1,Q88S82,Q88S49	Lactobacillales	Lactobacillus plantarum	2	1	0	1	0	0	0	0	1
387344	Lactobacillus brevis (strain ATCC 367 / JCM 1170).	Q03U09,Q03TR8,Q03Q60,Q03PR3,Q03RY9,Q03TX2	Lactobacillales	Lactobacillus brevis	2	1	0	2	1	0	0	0	0
321967	Lactobacillus casei (strain ATCC 334).	Q034J6,Q03BB9,Q03CI1	Lactobacillales	Lactobacillus casei	2	0	0	1	0	0	0	0	0
543734	Lactobacillus casei (strain BL23).	B3W845,B3W8Q2,B3W7H9,B3WB05,B3WBB7,B3WBC7	Lactobacillales	Lactobacillus casei	2	0	0	1	0	0	0	0	0
1351	Enterococcus faecalis (Streptococcus faecalis).	O34154,Q8KU77,Q838L3,Q82Z43	Lactobacillales	Enterococcus faecalis	1	0	0	1	1	0	0	0	1
1360	Lactococcus lactis subsp. lactis (Streptococcus lactis).	Q9X423,Q9X419,Q9CDN5,Q9CFG8,Q9CG64	Lactobacillales	Lactococcus lactis	1	0	0	1	3	0	0	0	0
416870	Lactococcus lactis subsp. cremoris (strain MG1363).	A2RK83,A2RNY7	Lactobacillales	Lactococcus lactis	1	0	0	1	0	0	0	0	0
272622	Lactococcus lactis subsp. cremoris (strain SK11).	Q02YH8,Q02VU6	Lactobacillales	Lactococcus lactis	1	0	0	1	0	0	0	0	0
388919	Streptococcus sanguinis (strain SK36).	A3CPV3,A3CQJ7	Lactobacillales	Streptococcus sanguinis	1	0	0	0	0	0	0	0	0
29390	Streptococcus gordonii (strain Challis / ATCC 35105 / CH1 / DL1 /V288).	A8AVX5	Lactobacillales	Streptococcus gordonii	1	0	0	0	0	0	0	0	0
487214	Streptococcus pneumoniae (strain Hungary19A-6).	B1I9X7,B1I9Z6	Lactobacillales	Streptococcus pneumoniae	1	0	0	0	0	0	0	1	0
516950	Streptococcus pneumoniae (strain CGSP14).	B2INI6,B2INK5	Lactobacillales	Streptococcus pneumoniae	1	0	0	0	0	0	0	1	0
1313	Streptococcus pneumoniae.	Q97N88,P63742	Lactobacillales	Streptococcus pneumoniae	1	0	0	0	0	0	0	1	0
171101	Streptococcus pneumoniae (strain ATCC BAA-255 / R6).	P63743,Q8DN16	Lactobacillales	Streptococcus pneumoniae	1	0	0	0	0	0	0	1	0
373153	Streptococcus pneumoniae serotype 2 (strain D39 / NCTC 7466).	Q04I07,Q04HZ0	Lactobacillales	Streptococcus pneumoniae	1	0	0	0	0	0	0	1	0
216495	Streptococcus agalactiae serotype III.	Q8E794,Q8E3B7	Lactobacillales	Streptococcus agalactiae	1	0	0	0	0	0	0	0	0
355315	Streptococcus agalactiae serotype Ia.	Q3K3A6,Q3JZ83	Lactobacillales	Streptococcus agalactiae	1	0	0	0	0	0	0	0	0
216466	Streptococcus agalactiae serotype V.	Q8E1T0,Q8DXP7	Lactobacillales	Streptococcus agalactiae	1	0	0	0	0	0	0	0	0
319700	Streptococcus pyogenes serotype M28.	Q48RX6	Lactobacillales	Streptococcus pyogenes	1	0	0	0	0	0	0	0	0
370552	Streptococcus pyogenes serotype M2 (strain MGAS10270).	Q1JFJ5	Lactobacillales	Streptococcus pyogenes	1	0	0	0	0	0	0	0	0
370553	Streptococcus pyogenes serotype M12 (strain MGAS2096).	Q1JAF2	Lactobacillales	Streptococcus pyogenes	1	0	0	0	0	0	0	0	0
370551	Streptococcus pyogenes serotype M12 (strain MGAS9429).	Q1JKK3	Lactobacillales	Streptococcus pyogenes	1	0	0	0	0	0	0	0	0
301450	Streptococcus pyogenes serotype M6.	Q5XAJ9	Lactobacillales	Streptococcus pyogenes	1	0	0	0	0	0	0	0	0
160491	Streptococcus pyogenes serotype M5 (strain Manfredo).	A2RD27	Lactobacillales	Streptococcus pyogenes	1	0	0	0	0	0	0	0	0
370554	Streptococcus pyogenes serotype M4 (strain MGAS10750).	Q1J5E4	Lactobacillales	Streptococcus pyogenes	1	0	0	0	0	0	0	0	0
301451	Streptococcus pyogenes serotype M18.	Q8NZW9	Lactobacillales	Streptococcus pyogenes	1	0	0	0	0	0	0	0	0
301447	Streptococcus pyogenes serotype M1.	Q99YI7	Lactobacillales	Streptococcus pyogenes	1	0	0	0	0	0	0	0	0
301448	Streptococcus pyogenes serotype M3.	Q8K665	Lactobacillales	Streptococcus pyogenes	1	0	0	0	0	0	0	0	0
342451	Staphylococcus saprophyticus subsp. saprophyticus (strain ATCC 15305 /DSM 20229).	Q49V87,Q49X93,Q49ZR6,Q4A060,Q49UE8,Q4A099	Bacillales_2	Staphylococcus saprophyticus	1	0	0	1	0	0	0	0	0

279808	Staphylococcus haemolyticus (strain JCSC1435).	Q4LA08,Q4L607,Q4L8Z1,Q4L930	Bacillales_2	Staphylococcus haemolyticus	1	0	0	1	0	0	0	0	0
176279	Staphylococcus epidermidis (strain ATCC 35984 / RP62A).	Q5HLC9,Q5HLQ6,Q5HL88,Q5HPP1	Bacillales_2	Staphylococcus epidermidis	1	0	0	1	0	0	0	0	0
176280	Staphylococcus epidermidis (strain ATCC 12228).	Q8CSS0,Q8CR47,Q8CR66,Q8CRC6	Bacillales_2	Staphylococcus epidermidis	1	0	0	1	0	0	0	0	0
196620	Staphylococcus aureus (strain MW2).	Q8NXY1,Q8NUU6,Q8NWX7	Bacillales_2	Staphylococcus aureus	1	0	0	1	0	0	0	0	0
282459	Staphylococcus aureus (strain MSSA476).	Q6GBT5,Q6G9R3,Q6G6H0	Bacillales_2	Staphylococcus aureus	1	0	0	1	0	0	0	0	0
426430	Staphylococcus aureus (strain Newman).	A6QJZ2,A6QEK4,A6QGJ8	Bacillales_2	Staphylococcus aureus	1	0	0	1	0	0	0	0	0
93062	Staphylococcus aureus (strain COL).	Q5HD48,Q5HIC3,Q5HGD2	Bacillales_2	Staphylococcus aureus	1	0	0	1	0	0	0	0	0
367830	Staphylococcus aureus (strain USA300).	Q2FHD9,Q2FE01,Q2FJ88	Bacillales_2	Staphylococcus aureus	1	0	0	1	0	0	0	0	0
451516	Staphylococcus aureus (strain USA300 / TCH1516).	A8YZP9,A8Z1X0,A8YYQ5	Bacillales_2	Staphylococcus aureus	1	0	0	1	0	0	0	0	0
93061	Staphylococcus aureus (strain NCTC 8325).	Q2FYZ5,Q2G0M6,Q2G205	Bacillales_2	Staphylococcus aureus	1	0	0	1	0	0	0	0	0
273036	Staphylococcus aureus (strain bovine RF122 / ET3-1).	Q2YSA9,Q2YXR6,Q2YW59	Bacillales_2	Staphylococcus aureus	1	0	0	1	0	0	0	0	0
282458	Staphylococcus aureus (strain MRSA252).	Q6GJB6,Q6GHD5,Q6GDU2	Bacillales_2	Staphylococcus aureus	1	0	0	1	0	0	0	0	0
359787	Staphylococcus aureus (strain JH1).	A6TZ29,A6U4N6,A6U1B8	Bacillales_2	Staphylococcus aureus	1	0	0	1	0	0	0	0	0
359786	Staphylococcus aureus (strain JH9).	A5IQA6,A5ISI2,A5IVT3	Bacillales_2	Staphylococcus aureus	1	0	0	1	0	0	0	0	0
158879	Staphylococcus aureus (strain N315).	P63550,P99113,Q7A3J4	Bacillales_2	Staphylococcus aureus	1	0	0	1	0	0	0	0	0
158878	Staphylococcus aureus (strain Mu50 / ATCC 700699).	Q99RC9,P63549,P63741	Bacillales_2	Staphylococcus aureus	1	0	0	1	0	0	0	0	0
418127	Staphylococcus aureus (strain Mu3 / ATCC 700698).	A7X1U3,A7WYY2,A7X6J1	Bacillales_2	Staphylococcus aureus	1	0	0	1	0	0	0	0	0
1462	Geobacillus kaustophilus.	Q5L091,Q5KYL0,Q5KYP6,Q5KYS7	Bacillales_2	Geobacillus kaustophilus	1	1	0	0	1	0	0	0	0
279010	Bacillus licheniformis (strain DSM 13 / ATCC 14580).	Q65M11,Q65J12,Q65GC1,Q65DK4,Q65EY0,Q65CW4	Bacillales_2	Bacillus licheniformis	1	2	0	1	1	0	0	0	1
315750	Bacillus pumilus (strain SAFR-032).	A8FJ29,A8FE32,A8FBF0,A8FFH8	Bacillales_2	Bacillus pumilus	1	1	0	1	1	0	0	0	0
222523	Bacillus cereus (strain ATCC 10987).	Q739D1,Q735Y4,Q734J8,Q73CE0,Q738U1	Bacillales_2	Bacillus cereus group	1	0	0	2	1	0	0	0	0
315730	Bacillus weihenstephanensis (strain KBAB4).	A9VU02,A9VMK7,A9VJG5,A9VI58	Bacillales_2	Bacillus cereus group	1	0	0	2	0	0	0	0	0
226900	Bacillus cereus (strain ATCC 14579 / DSM 31).	Q81GZ2,Q81C19,Q81DX3,Q81B23	Bacillales_2	Bacillus cereus group	1	0	0	2	0	0	0	0	0
288681	Bacillus cereus (strain ZK / E33L).	Q63H40,Q638K2,Q639Y2,Q63EX2	Bacillales_2	Bacillus cereus group	1	0	0	2	0	0	0	0	0
1392	Bacillus anthracis.	Q6I4N1,Q81MZ1,Q81U58	Bacillales_2	Bacillus anthracis	1	0	0	1	0	0	0	0	0
412694	Bacillus thuringiensis (strain Al Hakam).	A0RAP3,A0RFF4,A0RGG3,A0R8M9	Bacillales_2	Bacillus thuringiensis	1	0	0	2	0	0	0	0	0
180856	Bacillus thuringiensis subsp. konkukian.	Q6HPK8,Q6HHD4,Q6HMD5,Q6HG42	Bacillales_2	Bacillus thuringiensis	1	0	0	2	0	0	0	0	0
246194	Carboxydothermus hydrogenoformans (strain Z-2901 / DSM 6008).	Q3AB25	Thermoanaerobacterales_2	Carboxydothermus hydrogenoformans	1	0	0	0	0	0	0	0	0
349161	Desulfotomaculum reducens (strain MI-1).	A4J8E6	Clostridiales_2	Desulfotomaculum reducens	1	0	0	0	0	0	0	0	0
76856	Fusobacterium nucleatum subsp. nucleatum.	Q8RHZ9	Fusobacteriales	Fusobacterium nucleatum	1	0	0	0	0	0	0	0	0
139	Borrelia burgdorferi (Lyme disease spirochete).	A5JJN1,A5JJM5,A5JJN5,A5JJM9,O51257,A5JJM8,O51495,A5JJN4,A5JJM7,A5JJM6	Spirochaetales	Borrelia burgdorferi	1	0	0	0	0	0	0	0	0
390236	Borrelia afzelii (strain PKo).	Q0SMW4,A5JJP0,Q0SNS0	Spirochaetales	Borrelia afzelii	1	0	0	0	0	0	0	0	0
349741	Akkermansia muciniphila (strain ATCC BAA-835).	B2UN37	Verrucomicrobiae	Akkermansia muciniphila	0	0	0	0	0	0	0	1	0
435591	Parabacteroides distasonis (strain ATCC 8503 / DSM 20701 / NCTC11152).	A6LCZ1,A6LBL9,A6LA38	Bacteroidia	Parabacteroides distasonis	1	0	0	0	1	0	0	0	1
411154	Gramella forsetii (strain KT0803).	A0LZ79	Flavobacteria	Gramella forsetii	0	1	0	0	0	0	0	0	0
376686	Flavobacterium johnsoniae (strain ATCC 17061 / DSM 2064 / UW101)(Cytophaga johnsonae).	A5FID2,A5FKW1,A5FC42	Flavobacteria	Flavobacterium johnsoniae	0	1	0	0	0	0	0	0	0
448385	Sorangium cellulosum (strain So ce56) (Polyangium cellulosum (strainSo ce56)).	A9GMQ4,A9GEB5,A9FXQ6,A9F5A4	Deltaproteobacteria	Sorangium cellulosum	1	1	0	0	1	0	0	0	0

882	Desulfovibrio vulgaris (strain Hildenborough / ATCC 29579 / NCIMB8303).	Q726H4	Deltaproteobacteria	Desulfovibrio vulgaris	1	0	0	0	0	0	0	0	0
391774	Desulfovibrio vulgaris subsp. vulgaris (strain DP4).	A1VA10	Deltaproteobacteria	Desulfovibrio vulgaris	1	0	0	0	0	0	0	0	0
269799	Geobacter metallireducens (strain GS-15 / ATCC 53774 / DSM 7210).	Q39V17	Deltaproteobacteria	Geobacter metallireducens	1	0	0	0	0	0	0	0	0
35554	Geobacter sulfurreducens.	Q749I1	Deltaproteobacteria	Geobacter sulfurreducens	1	0	0	0	0	0	0	0	0
204669	Acidobacteria bacterium (strain Ellin345).	Q1IMB2,Q1IUX3,Q1IT93	unclassifed Acidobacteria	Acidobacteria bacterium	1	1	0	0	0	0	0	0	0
279238	Novosphingobium aromaticivorans (strain DSM 12444).	Q2GAC0,Q2G5J6,Q2GC90	Sphingomonadales	Novosphingobium aromaticivorans	2	0	0	0	1	0	0	0	0
272943	Rhodobacter sphaeroides (strain ATCC 17023 / 2.4.1 / NCIB 8253 / DSM158).	Q3J317,Q3IYM3,Q3IWH5,Q3IVW9	Rhodobacterales	Rhodobacter sphaeroides	1	0	1	0	1	0	0	0	0
375451	Roseobacter denitrificans (strain ATCC 33942 / OCh 114) (Erythrobactersp. (strain OCh 114)) (Roseobacter denitrificans).	Q162B7,Q162J8,Q162I2,Q165D5,Q161W5,Q162I7	Rhodobacterales	Roseobacter denitrificans	1	0	0	0	1	0	1	0	0
375	Bradyrhizobium japonicum.	Q89UK6,Q89VC8,Q89QA4,Q89I54	Rhizobiales	Bradyrhizobium japonicum	1	0	1	0	1	0	0	0	0
288000	Bradyrhizobium sp. (strain BTAi1 / ATCC BAA-1182).	A5EGP3,A5EGN6,A5EFA9,A5ER11,A5EAI3,A5ES06	Rhizobiales	Bradyrhizobium sp.	1	0	1	0	2	0	1	0	0
114615	Bradyrhizobium sp. (strain ORS278).	A4Z243,A4YQL2,A4YN13,A4YX95,A4YLX6,A4YXA2	Rhizobiales	Bradyrhizobium sp.	1	0	1	0	2	0	1	0	0
381	Rhizobium loti (Mesorhizobium loti).	Q986P3,Q98FV3,Q98CS0,Q98M73,Q98D08	Rhizobiales	Rhizobium loti	1	0	1	0	1	0	1	0	0
359391	Brucella abortus (strain 2308).	Q2YIQ1,Q2YMQ1,Q2YJV6	Rhizobiales	Brucella abortus	0	0	1	0	1	0	1	0	0
430066	Brucella abortus (strain S19).	B2SAH1,B2SA38,B2SBW7	Rhizobiales	Brucella abortus	0	0	1	0	1	0	1	0	0
470137	Brucella suis (strain ATCC 23445 / NCTC 10510).	A9WYC6,B0CKN0,A9WZF0,A9WXX0	Rhizobiales	Brucella suis	1	0	1	0	1	0	1	0	0
29461	Brucella suis.	Q8FVH7,Q8G203,Q8FX21,Q8FWK8	Rhizobiales	Brucella suis	1	0	1	0	1	0	1	0	0
382	Rhizobium meliloti (Sinorhizobium meliloti).	Q92LW8,Q92UI5,Q92N09,Q92VV3,Q92NH0,Q92MQ2,O86033,Q92NH4,Q92MP4,Q92S06,Q92MQ1,Q92W66	Rhizobiales	Rhizobium meliloti	1	0	1	0	1	0	1	0	0
216596	Rhizobium leguminosarum bv. viciae (strain 3841).	Q1M4R1,Q1MBI2,Q1M8S5,Q1M8P9,Q1MBL7,Q1MDC1,Q1M4Q5,Q1MFR7,Q1M354	Rhizobiales	Rhizobium leguminosarum	2	0	2	0	1	0	1	0	0
536	Chromobacterium violaceum.	Q7P1G2,Q7NWW7	Neisseriales	Chromobacterium violaceum	1	0	0	0	0	0	0	0	0
420662	Methylibium petroleiphilum (strain PM1).	A2SM29	Burkholderiales	Methylibium petroleiphilum	1	0	0	0	0	0	0	0	0
320372	Burkholderia pseudomallei (strain 1710b).	Q3JVE5,Q3JJF2,Q3JSI5,Q3JVT4,Q3JVG9,Q3JM07	Burkholderiales	Burkholderia pseudomallei	1	0	0	0	2	0	0	0	0
28450	Burkholderia pseudomallei (Pseudomonas pseudomallei).	Q63WQ3,Q63KX5,Q63WS4,Q63IP3,Q63X50	Burkholderiales	Burkholderia pseudomallei	1	0	0	0	2	0	0	0	0
13373	Burkholderia mallei (Pseudomonas mallei).	Q62C36,Q62MJ6,Q62K94,Q62MA7	Burkholderiales	Burkholderia mallei	1	0	0	0	2	0	0	0	0
269482	Burkholderia vietnamiensis (strain G4 / LMG 22486) (Burkholderiacepacia (strain R1808)).	A4JHM8,A4JHF7,A4JSU4,A4JHE1	Burkholderiales	Burkholderia vietnamiensis	1	0	0	0	2	0	0	0	0
269483	Burkholderia sp. (strain 383) (Burkholderia cepacia (strain ATCC 17760/ NCIB 9086 / R18194)).	Q398V9,Q39DD6,Q390D5,Q39DF5,Q394C3,Q39D66	Burkholderiales	Burkholderia sp.	1	0	0	0	2	0	0	0	0
406425	Burkholderia cenocepacia (strain MC0-3).	B1JXW6,B1JY43,B1KB46,B1JXJ6,B1K8Z4,B1K937	Burkholderiales	Burkholderia cenocepacia	1	0	0	0	3	0	0	0	0
331272	Burkholderia cenocepacia (strain HI2424).	A0KA28,A0KA09,A0B4F1,A0KAA1,A0B1M4,A0KE55	Burkholderiales	Burkholderia cenocepacia	1	0	0	0	3	0	0	0	0
331271	Burkholderia cenocepacia (strain AU 1054).	Q1BTT7,Q1BU28,Q1BG91,Q1BU09,Q1BKB4,Q1BPL0	Burkholderiales	Burkholderia cenocepacia	1	0	0	0	3	0	0	0	0
339670	Burkholderia ambifaria (strain ATCC BAA-244 / AMMD) (Burkholderiacepacia (strain AMMD)).	Q0BCD3,Q0B1U6,Q0BCB5,Q0BC36,Q0B7X3	Burkholderiales	Burkholderia ambifaria	1	0	0	0	2	0	0	0	0

266265	Burkholderia xenovorans (strain LB400).	Q13UL7,Q13UE3,Q13T89,Q13UN4,Q13KW6	Burkholderiales	Burkholderia xenovorans	1	0	0	0	2	0	0	0	0
266264	Ralstonia metallidurans (strain CH34 / ATCC 43123 / DSM 2839).	Q1LL59,Q1LC22	Burkholderiales	Ralstonia metallidurans	1	0	0	0	0	0	0	0	0
183190	Xylella fastidiosa (strain Temecula1 / ATCC 700964).	Q87BZ2	Xanthomonadales	Xylella fastidiosa	1	0	0	0	0	0	0	0	0
405441	Xylella fastidiosa (strain M23).	B2I618	Xanthomonadales	Xylella fastidiosa	1	0	0	0	0	0	0	0	0
522373	Stenotrophomonas maltophilia (strain K279a).	B2FI02	Xanthomonadales	Stenotrophomonas maltophilia	1	0	0	0	0	0	0	0	0
316273	Xanthomonas campestris pv. vesicatoria (strain 85-10).	Q3BYR0,Q3BUM5,Q3BMD2	Xanthomonadales	Xanthomonas campestris	1	0	0	0	1	0	0	0	0
314565	Xanthomonas campestris pv. campestris (strain 8004).	Q4UZR8,Q4UTU5	Xanthomonadales	Xanthomonas campestris	1	0	0	0	1	0	0	0	0
349124	Halorhodospira halophila (strain DSM 244 / SL1) (Ectothiorhodospirahalophila (strain DSM 244 / SL1)).	A1WT61	Chromatiales	Halorhodospira halophila	1	0	0	0	0	0	0	0	0
246195	Dichelobacter nodosus (strain VCS1703A).	A5EWH1	Cardiobacteriales	Dichelobacter nodosus	1	0	0	0	0	0	0	0	0
484022	Francisella philomiragia subsp. philomiragia (strain ATCC 25017).	B0TWZ7	Thiotrichales	Francisella philomiragia	1	0	0	0	0	0	0	0	0
441952	Francisella tularensis subsp. mediasiatica (strain FSC147).	B2SF32	Thiotrichales	Francisella tularensis	1	0	0	0	0	0	0	0	0
418136	Francisella tularensis subsp. tularensis (strain WY96-3418).	A4IW85	Thiotrichales	Francisella tularensis	1	0	0	0	0	0	0	0	0
393115	Francisella tularensis subsp. tularensis (strain FSC 198).	Q14JU8	Thiotrichales	Francisella tularensis	1	0	0	0	0	0	0	0	0
119856	Francisella tularensis subsp. tularensis.	Q5NIE5	Thiotrichales	Francisella tularensis	1	0	0	0	0	0	0	0	0
434922	Coxiella burnetii (strain Dugway 5J108-111).	A9KGM0,A9KCL1	Legionellales	Coxiella burnetii	1	0	0	0	1	0	0	0	0
777	Coxiella burnetii.	Q83D14,Q83EH5	Legionellales	Coxiella burnetii	1	0	0	0	1	0	0	0	0
498211	Cellvibrio japonicus (strain Ueda107) (Pseudomonas fluorescens subsp.cellulosa).	B3PES0,B3PDA4,B3PD59	Pseudomonadales_1	Cellvibrio japonicus	0	1	0	0	0	0	0	0	0
203122	Saccharophagus degradans (strain 2-40 / ATCC 43961 / DSM 17024).	Q21HR4,Q21MP5,Q21KN7	Alteromonadales_1	Saccharophagus degradans	0	1	0	0	0	0	0	0	0
381754	Pseudomonas aeruginosa (strain PA7).	A6V5E5,A6V371,A6V1L3,A6V1L0	Pseudomonadales_2	Pseudomonas aeruginosa	2	0	0	0	1	0	0	0	0
208963	Pseudomonas aeruginosa (strain UCBPP-PA14).	Q02R51,Q02PI8,Q02R54,Q02JX0,Q02MD6	Pseudomonadales_2	Pseudomonas aeruginosa	2	0	0	0	1	0	0	0	0
287	Pseudomonas aeruginosa.	Q9HZI2,Q9HY41,Q51390,Q9I1D5,Q9I3M5	Pseudomonadales_2	Pseudomonas aeruginosa	2	0	0	0	1	0	0	0	0
379731	Pseudomonas stutzeri (strain A1501).	A4VLK7,A4VJY7	Pseudomonadales_2	Pseudomonas stutzeri	1	0	0	0	1	0	0	0	0
399739	Pseudomonas mendocina (strain ymp).	A4Y0V4,A4XSK8,A4XXN1	Pseudomonadales_2	Pseudomonas mendocina	1	0	0	0	1	0	0	0	0
351746	Pseudomonas putida (strain F1 / ATCC 700007).	A5VZG7	Pseudomonadales_2	Pseudomonas putida	1	0	0	0	0	0	0	0	0
205922	Pseudomonas fluorescens (strain Pf0-1).	Q3K7I5,Q3KCX9	Pseudomonadales_2	Pseudomonas fluorescens	1	0	0	0	1	0	0	0	0
220664	Pseudomonas fluorescens (strain Pf-5 / ATCC BAA-477).	Q4KD08,Q4K734,Q4KC52,Q4KJE0	Pseudomonadales_2	Pseudomonas fluorescens	1	0	0	0	1	0	0	0	0
264730	Pseudomonas syringae pv. phaseolicola (strain 1448A / Race 6).	Q48F01,Q48IJ4,Q48I56	Pseudomonadales_2	Pseudomonas syringae	1	0	0	0	1	0	0	0	0
205918	Pseudomonas syringae pv. syringae (strain B728a).	Q4ZTP8,Q4ZPI7	Pseudomonadales_2	Pseudomonas syringae	1	0	0	0	1	0	0	0	0
349521	Hahella chejuensis (strain KCTC 2396).	Q2SJ96,Q2SHL3,Q2S6Z4,Q2SDF1	Oceanospirillales	Hahella chejuensis	1	0	0	0	0	0	0	0	0
400668	Marinomonas sp. (strain MWYL1).	A6VWT3,A6W2C1,A6VU90,A6VWG3,A6VWT2	Oceanospirillales	Marinomonas sp MWYL1	1	0	0	0	3	0	0	0	0
290398	Chromohalobacter salexigens (strain DSM 3043 / ATCC BAA-138 / NCIMB13768).	Q1QZV2,Q1QVQ3	Oceanospirillales	Chromohalobacter salexigens (strain DSM 3043 / ATCC BAA-138 / NCIMB13768).	1	0	0	0	1	0	0	0	0
326442	Pseudoalteromonas haloplanktis (strain TAC 125).	Q3ICL7,Q3IK59	Alteromonadales_2	Pseudoalteromonas haloplanktis	2	0	0	0	0	0	0	0	0
342610	Pseudoalteromonas atlantica (strain T6c / BAA-1087).	Q15PG1,Q15Q03	Alteromonadales_2	Pseudoalteromonas atlantica	1	0	0	0	1	0	0	0	0
357804	Psychromonas ingrahamii (strain 37).	A1SZE1,A1SRH6	Alteromonadales_2	Psychromonas ingrahamii	1	0	0	0	1	0	0	0	0

319224	Shewanella putrefaciens (strain CN-32 / ATCC BAA-453).	A4Y756,A4Y2M5	Alteromonadales_2	Shewanella putrefaciens	1	1	0	0	0	0	0	0	0
351745	Shewanella sp. (strain W3-18-1).	A1RJD4,A1REY5	Alteromonadales_2	Shewanella sp.	1	1	0	0	0	0	0	0	0
60481	Shewanella sp. (strain MR-7).	Q0HV69,Q0HZS9	Alteromonadales_2	Shewanella sp.	1	1	0	0	0	0	0	0	0
60480	Shewanella sp. (strain MR-4).	Q0HE70,Q0HIR7	Alteromonadales_2	Shewanella sp.	1	1	0	0	0	0	0	0	0
94122	Shewanella sp. (strain ANA-3).	A0L1Q5,A0KWX5	Alteromonadales_2	Shewanella sp.	1	1	0	0	0	0	0	0	0
382245	Aeromonas salmonicida (strain A449).	A4SPA7	Aeromonadales	Aeromonas salmonicida	1	0	0	0	0	0	0	0	0
74109	Photobacterium profundum (Photobacterium sp. (strain SS9)).	Q6LJD6,Q6LVI8,Q6LUX7	Vibrionales	Photobacterium profundum	1	0	0	0	1	0	0	0	0
312309	Vibrio fischeri (strain ATCC 700601 / ES114).	Q5E0Z0	Vibrionales	Vibrio fischeri	1	0	0	0	0	0	0	0	0
338187	Vibrio harveyi (strain ATCC BAA-1116 / BB120).	A7N1R1	Vibrionales	Vibrio harveyi	1	0	0	0	0	0	0	0	0
670	Vibrio parahaemolyticus.	Q87M72,Q87FK5	Vibrionales	Vibrio parahaemolyticus	1	1	0	0	0	0	0	0	0
196600	Vibrio vulnificus (strain YJ016).	Q7MI93,Q7MJI3	Vibrionales	Vibrio vulnificus	1	0	0	0	0	0	0	1	0
672	Vibrio vulnificus.	Q8DBM6	Vibrionales	Vibrio vulnificus	1	0	0	0	0	0	0	0	0
345073	Vibrio cholerae serotype O1 (strain ATCC 39541 / Ogawa 395 / O395).	C3M5J0,A5EZR2	Vibrionales	Vibrio cholerae	2	0	0	0	0	0	0	0	0
434271	Actinobacillus pleuropneumoniae serotype 3 (strain JL03).	B0BSB2,B0BTI8,B0BRN7,B0BTM9	Pasteurellales	Actinobacillus pleuropneumoniae	1	0	0	0	1	0	0	1	0
537457	Actinobacillus pleuropneumoniae serotype 7 (strain AP76).	B3GYP7,B3H2X8,B3H2T1,B3H0N9	Pasteurellales	Actinobacillus pleuropneumoniae	1	0	0	0	1	0	0	1	0
416269	Actinobacillus pleuropneumoniae serotype 5b (strain L20).	A3MZ93,A3N2L2,A3N3K1,A3N2Y3	Pasteurellales	Actinobacillus pleuropneumoniae	1	0	0	0	1	0	0	1	0
374930	Haemophilus influenzae (strain PittEE).	A5UDU8,A5UAS7,A5UCZ1,A5UE44	Pasteurellales	Haemophilus influenzae	1	0	0	0	0	0	0	1	0
374931	Haemophilus influenzae (strain PittGG).	A5UIN8,A5UIF7,A5UHH7	Pasteurellales	Haemophilus influenzae	1	0	0	0	1	1	0	0	0
727	Haemophilus influenzae.	P44991,P44401,P44399,P44400	Pasteurellales	Haemophilus influenzae	1	0	0	0	1	1	0	1	0
339671	Actinobacillus succinogenes (strain ATCC 55618 / 130Z).	A6VKG8,A6VLM9,A6VKQ2,A6VKL2,A6VLM1,A6VL05,A6VPR1	Pasteurellales	Actinobacillus succinogenes	1	1	0	0	1	2	0	0	0
221988	Mannheimia succiniciproducens (strain MBEL55E).	Q65S44,Q65R15,Q65WJ4,Q65SP2,Q65Q24,Q65WK5,Q65PY1	Pasteurellales	Mannheimia succiniciproducens	1	1	0	0	1	2	0	0	1
228400	Haemophilus somnus (strain 2336) (Histophilus somni (strain 2336)).	B0UT20,B0UWY8,B0US23,B0UTW2,B0UX03	Pasteurellales	Haemophilus somnus	0	1	0	0	1	1	0	1	0
205914	Haemophilus somnus (strain 129Pt) (Histophilus somni (strain 129Pt)).	Q0I173,Q0I2L5,Q0I5M6,Q0I345,Q0I188	Pasteurellales	Haemophilus somnus	0	1	0	0	1	1	0	1	0
343509	Sodalis glossinidius (strain morsitans).	Q2NQX8,Q2NVD9,Q2NWM0	Enterobacteriales	Sodalis glossinidius	1	0	0	0	1	0	0	1	0
393305	Yersinia enterocolitica serotype O:8 / biotype 1B (strain 8081).	A1JI03,A1JTE5,A1JJZ5,A1JJ57,A1JT12,A1JMC1	Enterobacteriales	Yersinia enterocolitica	1	1	0	0	1	0	0	0	0
349746	Yersinia pestis bv. Antiqua (strain Angola).	A9R6B5,A9QYS1,A9R0W5,A9R208,A9R8V2,A9R5Q2,A9R0Y7,A9R072,A9R4Q6	Enterobacteriales	Yersinia pestis/pseudotuberculosis	1	1	0	0	1	0	0	0	1
360102	Yersinia pestis bv. Antiqua (strain Antiqua).	Q1C428,Q1C2A9,Q1C0V9,Q1C996,Q1C1R0,Q1C0D2,Q1C142,Q1C450,Q1C7J2	Enterobacteriales	Yersinia pestis/pseudotuberculosis	1	1	0	0	1	0	0	0	1
377628	Yersinia pestis bv. Antiqua (strain Nepal516).	Q1CEB3,Q1CG65,C4GNI4,Q1CDS0,Q1CDB7,C4GT19,Q1CLL7,Q1CD45,C4GY58,Q1CIX8,Q1CN11,Q1CLN9	Enterobacteriales	Yersinia pestis/pseudotuberculosis	1	2	0	0	1	0	0	0	2
502800	Yersinia pseudotuberculosis serotype O:3 (strain YPIII).	B1JQ85,B1JH39,B1JKM6,B1JNC8,B1JJD0,B1JLP8,B1JJA8,B1JKD3,B1JS20	Enterobacteriales	Yersinia pestis/pseudotuberculosis	1	1	0	0	1	0	0	0	1
386656	Yersinia pestis (strain Pestoides F).	A4TJ18,A4TQL2,A4TNB8,A4TS96,A4TRT0,A4TS64,A4TQ23,A4THA1,A4TQ44	Enterobacteriales	Yersinia pestis/pseudotuberculosis	1	1	0	0	1	0	0	0	1
502801	Yersinia pseudotuberculosis serotype IB (strain PB1/+).	B2K4K8,B2K3G3,B2JZB6,B2K1W2,B2K5A5,B2K5X1,B2K7D3,B2K4T3,B2K9H7	Enterobacteriales	Yersinia pestis/pseudotuberculosis	1	1	0	0	1	0	0	0	1

633	Yersinia pseudotuberculosis.	Q66AF7,Q66EY7,Q66G96,Q665C6,Q66FF3,Q66E75,Q663Y2,Q66CS9,Q66E96	Enterobacteriales	Yersinia pestis/pseudotuberculosis	1	1	0	0	1	0	0	0	1
349747	Yersinia pseudotuberculosis serotype O:1b (strain IP 31758).	A7FMJ5,A7FK78,A7FHY3,A7FLU9,A7FCX3,A7FLS6,A7FN81,A7FP69,A7FDN3	Enterobacteriales	Yersinia pestis/pseudotuberculosis	1	1	0	0	1	0	0	0	1
632	Yersinia pestis.	P58543,Q7CHE4,Q7CKB4,Q7CKA0,Q0WB16,Q0WJP7,Q8ZJ02,Q8D1N4,Q8D1T6,Q8CZH7,Q74VK5,Q74QR1	Enterobacteriales	Yersinia pestis/pseudotuberculosis	1	1	0	0	2	0	0	0	1
29471	Erwinia carotovora subsp. atroseptica (Pectobacterium atrosepticum).	Q6CZ86,Q6D5T8,Q6DB06,Q6DA23	Enterobacteriales	Erwinia carotovora	1	0	0	0	1	0	0	0	1
399741	Serratia proteamaculans (strain 568).	A8G7W7,A8GIU1,A8GL00,A8GBJ6,A8GLA1,A8GEM5,A8GFA3	Enterobacteriales	Serratia proteamaculans	1	0	0	0	1	1	0	0	0
399742	Enterobacter sp. (strain 638).	A4WG72,A4W567,A4WER6,A4WG92,A4W6G7	Enterobacteriales	Enterobacter sp.	1	1	0	0	1	0	0	0	1
272620	Klebsiella pneumoniae subsp. pneumoniae (strain ATCC 700721 / MGH78578).	A6TBI9,A6TFR2,A6TD84,A6T620,A6TEC0,A6T664,A6T963,A6TFI0,A6TGA8,A6TBJ4	Enterobacteriales	Klebsiella pneumoniae	1	0	1	0	2	0	0	1	1
290338	Citrobacter koseri (strain ATCC BAA-895 / CDC 4225-83 / SGSC4696).	A8ARG8,A8AL00,A8AED4,A8AEC9,A8AP16,A8AL29,A8ANY5,A8ARF0	Enterobacteriales	Citrobacter koseri	1	0	0	0	2	1	0	1	1
41514	Salmonella arizonae (strain ATCC BAA-731 / CDC346-86 / RSK2980).	A9MLF2,A9MI66,A9MI40,A9MSA5	Enterobacteriales	Salmonella arizonae	1	0	0	0	1	0	0	1	1
272994	Salmonella paratyphi B (strain ATCC BAA-1250 / SPB7).	A9MUU9,A9MWF3,A9MYN9,A9MZH4,A9N2J3,A9MZC6,A9MZF8,A9MUW8	Enterobacteriales	Salmonella paratyphi	1	1	0	0	1	1	0	1	1
54388	Salmonella paratyphi A.	Q5PLM7,Q5PLP8,Q5PEK8,Q5PJE9,Q5PIS3,Q5PKG3,Q5PDF1,Q5PC71	Enterobacteriales	Salmonella paratyphi	1	1	0	0	1	1	0	1	1
90370	Salmonella typhi.	Q8Z2Y6,Q8Z2C9,Q8Z2B7,Q8Z428,P58542,Q8Z2X3,Q8Z2V4,Q8Z2K0,Q83ST8	Enterobacteriales	Salmonella typhi	1	1	0	0	2	1	0	1	1
300269	Shigella sonnei (strain Ss046).	Q3YY54,Q3YVU9,Q3YV72,Q3YV52	Enterobacteriales	Escherichia coli/Shigella	1	0	0	0	1	0	0	1	1
300267	Shigella dysenteriae serotype 1 (strain Sd197).	Q32A92,Q32CF4,Q32A69,Q32CB5	Enterobacteriales	Escherichia coli/Shigella	1	0	0	0	0	0	0	1	1
331111	Escherichia coli O139:H28 (strain E24377A / ETEC).	A7ZUE0,A7ZTB1,A7ZHF4,A7ZUB5,A7ZQL8,A7ZLW9,A7ZQP7,A7ZTC7	Enterobacteriales	Escherichia coli/Shigella	1	1	0	0	1	1	0	1	1
364106	Escherichia coli (strain UTI89 / UPEC).	Q1RGD6,Q1R512,Q1R415,Q1R7S3,Q1R531,Q1R3Z2,Q1R7N7,Q1R5D2	Enterobacteriales	Escherichia coli/Shigella	1	1	0	0	1	1	0	1	1
562	Escherichia coli.	Q8GQN5,C3SWV3,C3SX82,Q9F4L6,C3SN42,C3T9Z2,C3SX83,C3TR07,C3SJ97,C3SWV2,C3SJ02	Enterobacteriales	Escherichia coli/Shigella	1	1	0	0	1	0	0	2	1
439855	Escherichia coli (strain SMS-3-5 / SECEC).	B1LIE9,B1LFZ7,B1LJE2,B1LJC6,B1LMU4,B1LFA4,B1LQ99,B1LQZ7,B1LNM9	Enterobacteriales	Escherichia coli/Shigella	1	1	0	0	1	1	0	1	1
300268	Shigella boydii serotype 4 (strain Sb227).	Q31U64,Q31V54,Q326H2,Q31U85	Enterobacteriales	Escherichia coli/Shigella	1	1	0	0	1	0	0	0	1
344609	Shigella boydii serotype 18 (strain CDC 3083-94 / BS512).	B2TWC2,B2TVP5,B2U269	Enterobacteriales	Escherichia coli/Shigella	1	1	0	0	0	0	0	0	0
373384	Shigella flexneri serotype 5b (strain 8401).	Q0SY84,Q0SY98,Q0SZ94,Q0SY63,Q0T8D4	Enterobacteriales	Escherichia coli/Shigella	1	1	0	0	1	1	0	0	1
405955	Escherichia coli O1:K1 / APEC.	A1A7B0,A1AHA6,A1AEW1,A1AHC2,A1AEZ0,A1AIA2,A1AI81,A1AH22	Enterobacteriales	Escherichia coli/Shigella	1	1	0	0	1	1	0	1	1
362663	Escherichia coli O6:K15:H31 (strain 536 / UPEC).	Q0TE91,Q0TBN8,Q0TAD8,Q0TAF9,Q0TBM0,Q0TE56,Q0TLS7	Enterobacteriales	Escherichia coli/Shigella	1	1	0	0	1	1	0	1	1

331112	Escherichia coli O9:H4 (strain HS).	A8A3Q6,A7ZW13,A8A639,A8A3T8,A8A707,A8A622,A8A064,A8A731	Enterobacteriales	Escherichia coli/Shigella	1	1	0	0	1	1	0	1	1
481805	Escherichia coli (strain ATCC 8739 / DSM 1576 / Crooks).	B1IRU9,B1IZL3,B1IZM8,B1IU38,B1IU66,B1IRB5,B1IVH4,B1IVF3	Enterobacteriales	Escherichia coli/Shigella	1	1	0	0	1	1	0	1	1
316385	Escherichia coli (strain K12 / DH10B).	B1XB71,B1X8I0,B1XDI7,B1XE99,B1XB92,B1XDL2,B1X8J6	Enterobacteriales	Escherichia coli/Shigella	1	0	0	0	1	1	0	1	1
83333	Escherichia coli (strain K12).	P11553,P55138,P37677,P0A6F3,P08204,P09099,P32171,P77432	Enterobacteriales	Escherichia coli/Shigella	1	1	0	0	1	1	0	1	1
83334	Escherichia coli O157:H7.	P58541,Q8X5T0,P0A6F4,Q8X6R3,Q8X899,Q8X7R6,Q8XDM4,Q8XAY5	Enterobacteriales	Escherichia coli/Shigella	1	1	0	0	1	0	0	1	1
