# Supplementary material for: The FGGY Carbohydrate Kinase Family: Insights into the Evolution of Functional Specificities
Source: PLoS Comput Biol. 2011 Dec 22;7(12):e1002318. doi: 10.1371/journal.pcbi.1002318 (PMC3245297; doi:10.1371/journal.pcbi.1002318)
Supplement: Text S1 — The prediction of SDPs, combining sequence and structural information. (DOC) [file pcbi.1002318.s007.doc]

Text S1. The prediction of SDPs combining sequence and structural information

The specificity determining positions (SDPs) in our analyses were identified based on both the sequence and the structural information of FGGY kinases.

The sequence-based SDP prediction has been implemented in many tools that use a variety of methods based on information theory, entropy optimization, or machine-learning approaches [1,2,3,4]. Here we used the SDPpred server [1], which provides a web interface for the automated prediction and probability-based ranking of alignment columns in the large dataset we have. In an ideal case, the input of SDPpred should contain a definition of groups that are comparable in size and whose internal members are very well conserved in their signature positions. However, in the CARS dataset of FGGY kinases, the size of isofunctional groups vary greatly, from more than 150 proteins in the GlpK group to less than 10 proteins in the RbtK group. Additionally, the sequence similarities among some members of the large isofunctional groups (e.g. GlpK, AraB, XylB) are very low, suggesting a potential divergence of their signature positions. In order to minimize the differences of sizes among different isofunctional groups and the divergence of proteins within a group, we used the following procedure to perform the sequence-based prediction of SDPs in CARS (Figure S1): (i) we built a multiple sequence alignment (MSA) of all proteins in CARS using the MUSCLE program with default parameters [5]. This alignment serves as a “master” alignment that we used to prepare inputs for SDPpred and map SDPs onto different proteins, and it is divided into subsections based on the function and the sequence similarity clustering of proteins; (ii) we selected the largest clusters from each isofunctional group and built the input MSA for SDPpred by simply take out the relevant lines from the “master” alignment; (iii) we mapped the predicted SDPs from the selected clusters to the rest of proteins in CARS based on their positions in the “master” MSA; (iv) the signature (amino acids distribution) of SDPs in various clusters within a single isofunctional group can be determined and compared with each other. As a result, the sequence-based SDP prediction provided a list of rankings for each individual alignment position in the “master” alignment, and the ranking indicates the significance of the position in distinguishing different isofunctional groups.

The structure-based SDP prediction was made based on the average distance from a residue position to the substrate in three-dimensional protein structures. The residues were mapped from structures to the “master” MSA so that an average distance could be calculated for each alignment position. Here we analyzed 19 PDB structures that correspond to proteins included in the CARS dataset and are co-crystallized with their natural ligands. The threshold we used to select SDPs is an average distance of closer than 4 Å.

Figure S2 plots the SDPpred ranking of residue positions against their average distances to natural substrates. Five positions were selected from the “master” MSA based on a combination of the sequence- and structure-based SDP predictions. The false-positive predictions using the sequence-based SDPpred algorithm alone were indicated with filled black dots in the lower right, and those using structure-based information alone were shown in a dashed square in the upper left of Figure S2. The consensus of both helped to identify SDPs that are functionally relevant.

REFERENCES

1. Kalinina OV, Mironov AA, Gelfand MS, Rakhmaninova AB (2004) Automated selection of positions determining functional specificity of proteins by comparative analysis of orthologous groups in protein families. Protein Sci 13: 443-456.

2. Reva B, Antipin Y, Sander C (2007) Determinants of protein function revealed by combinatorial entropy optimization. Genome Biol 8: R232.

3. Capra JA, Singh M (2008) Characterization and prediction of residues determining protein functional specificity. Bioinformatics 24: 1473-1480.

4. Ye K, Feenstra KA, Heringa J, Ijzerman AP, Marchiori E (2008) Multi-RELIEF: a method to recognize specificity determining residues from multiple sequence alignments using a Machine-Learning approach for feature weighting. Bioinformatics 24: 18-25.

5. Edgar RC (2004) MUSCLE: multiple sequence alignment with high accuracy and high throughput. Nucleic Acids Res 32: 1792-1797.
