# Supplementary material for: The FGGY Carbohydrate Kinase Family: Insights into the Evolution of Functional Specificities
Source: PLoS Comput Biol. 2011 Dec 22;7(12):e1002318. doi: 10.1371/journal.pcbi.1002318 (PMC3245297; doi:10.1371/journal.pcbi.1002318)
Supplement: Text S2 — The hierarchical clustering of SDP signatures. (DOC) [file pcbi.1002318.s008.doc]

Text S2. The hierarchical clustering of SDP signatures

In order to mathematically evaluate the similarity and differences of the SDP signatures of various protein clusters, we performed a hierarchical clustering on the signatures using the hclust function provided by the R software package. The clustering was done on a similarity matrix showing the identity of pairs of SDP signatures, and the identity in turn was based on the correlation coefficients (CCs) between the position weight matrices (PWMs) of SDP signatures. The PWMs, which were built for each individual protein cluster in CARS, contain 20 rows and 5 columns that indicate the distribution of 20 amino acids on the 5 signature positions. The CCs between pairs of PWMs were calculated with the corr2 function in MATLAB, which returns a 2-D CCs value for matrices pairs.

Figure S3 shows a heatmap of the similarity matrix (Figure S3, A) and the result of hierarchical clustering (Figure S3, B). The protein cluster names and the names of their corresponding branches in the protein phylogenetic tree (Figure 2) were indicated in both the heatmap and the hierarchical SDP signature clusters (indicated with red boxes).
