# Supplementary material for: The FGGY Carbohydrate Kinase Family: Insights into the Evolution of Functional Specificities
Source: PLoS Comput Biol. 2011 Dec 22;7(12):e1002318. doi: 10.1371/journal.pcbi.1002318 (PMC3245297; doi:10.1371/journal.pcbi.1002318)
Supplement: Text S3 — Reconstruction and analysis of protein similarity networks. (DOC) [file pcbi.1002318.s009.doc]

Text S3. Reconstruction and analysis of protein similarity networks

In order to evaluate the functional relevance of our predicted SDPs and the signature residues derived from SDPs, we built protein similarity networks (PSN) for proteins in CARS based on their concatenated sequences of signature residues. As a comparison, we also built PSN based on the entire sequences of proteins. Here is a detailed description of the approach we adapted from [1] and used to build PSNs, and the same approach was used to build PSNs for both signatures and entire sequences: first, alignment were made with each pair of sequences using the “blast2seq” program from the NCBI toolkit [2] with a parameter of word-size equals to 1. The program returns an E-value for each pairwise alignment indicating the significance of an alignment (the lower the E-value, the more significant an alignment is); second, a threshold is chosen for the selection of sequence pairs that are significantly similar; finally, a network is built based on the pairwise E-values and the selected threshold. Each node in the network indicates a protein in CARS, and each edge in the network indicates that the pair of nodes linked by this edge has an alignment with E-value more significant than the selected threshold. The network was visualized using the Cytoscape software version 2.7 [3], and the nodes in the network were arranged using the yFiles organic layout method.

Here we tested a number of different thresholds for the PSNs of both the signatures and entire sequences. When using relatively high thresholds (Figure 4, E-value no more than 1e-4 for entire sequences and no more than 1 for signatures), the PSN of entire sequence formed a big hairball that cannot help distinguishing isofunctional groups, whereas the PSN of signatures showed some functional-based clustering that splits the entire FGGY protein space into tightly clustered and largely mono-functional groups. When we increase the stringency of our thresholds (E-value no more than 1e-90 for entire sequences and no more than 1e-3 for signatures), however, the PSN of both entire sequences and signatures showed similar level of functional-based protein clustering that further splits the FGGY protein space into isolated islands of mono-functional groups. The fact that signatures, which are only five amino acids in length, could reproduce the clustering based on entire sequences, which are on average more than 500 amino acids in length, is significant. Considering that the clustering correspond with the functional classification annotated in CARS, our result strongly support the functional relevance of the signature residues that is derived from SDP prediction.

REFERENCES

1. Nguyen TT, Brown S, Fedorov AA, Fedorov EV, Babbitt PC, et al. (2008) At the periphery of the amidohydrolase superfamily: Bh0493 from Bacillus halodurans catalyzes the isomerization of D-galacturonate to D-tagaturonate. Biochemistry 47: 1194-1206.

2. Tatusova TA, Madden TL (1999) BLAST 2 Sequences, a new tool for comparing protein and nucleotide sequences. FEMS Microbiol Lett 174: 247-250.

3. Shannon P, Markiel A, Ozier O, Baliga NS, Wang JT, et al. (2003) Cytoscape: a software environment for integrated models of biomolecular interaction networks. Genome Res 13: 2498-2504.
